# Dynamics and impact of homologous recombination on the evolution of *Legionella pneumophila*

**DOI:** 10.1371/journal.pgen.1006855

**Published:** 2017-06-26

**Authors:** Sophia David, Leonor Sánchez-Busó, Simon R. Harris, Pekka Marttinen, Christophe Rusniok, Carmen Buchrieser, Timothy G. Harrison, Julian Parkhill

**Affiliations:** 1 Pathogen Genomics, Wellcome Trust Sanger Institute, Cambridge, United Kingdom; 2 Respiratory and Vaccine Preventable Bacteria Reference Unit, Public Health England, London, United Kingdom; 3 Helsinki Institute for Information Technology HIIT, Department of Computer Science, Aalto University, Aalto, Espoo, Finland; 4 Institut Pasteur, Biologie des Bactéries Intracellulaires, Paris, France; 5 CNRS UMR 3525, Paris, France; Imperial College London, UNITED KINGDOM

## Abstract

*Legionella pneumophila* is an environmental bacterium and the causative agent of Legionnaires’ disease. Previous genomic studies have shown that recombination accounts for a high proportion (>96%) of diversity within several major disease-associated sequence types (STs) of *L*. *pneumophila*. This suggests that recombination represents a potentially important force shaping adaptation and virulence. Despite this, little is known about the biological effects of recombination in *L*. *pneumophila*, particularly with regards to homologous recombination (whereby genes are replaced with alternative allelic variants). Using newly available population genomic data, we have disentangled events arising from homologous and non-homologous recombination in six major disease-associated STs of *L*. *pneumophila (subsp*. *pneumophila)*, and subsequently performed a detailed characterisation of the dynamics and impact of homologous recombination. We identified genomic “hotspots” of homologous recombination that include regions containing outer membrane proteins, the lipopolysaccharide (LPS) region and Dot/Icm effectors, which provide interesting clues to the selection pressures faced by *L*. *pneumophila*. Inference of the origin of the recombined regions showed that isolates have most frequently imported DNA from isolates belonging to their own clade, but also occasionally from other major clades of the same subspecies. This supports the hypothesis that the possibility for horizontal exchange of new adaptations between major clades of the subspecies may have been a critical factor in the recent emergence of several clinically important STs from diverse genomic backgrounds. However, acquisition of recombined regions from another subspecies, *L*. *pneumophila subsp*. *fraseri*, was rarely observed, suggesting the existence of a recombination barrier and/or the possibility of ongoing speciation between the two subspecies. Finally, we suggest that multi-fragment recombination may occur in *L*. *pneumophila*, whereby multiple non-contiguous segments that originate from the same molecule of donor DNA are imported into a recipient genome during a single episode of recombination.

## Author summary

*Legionella pneumophila* is an environmental bacterium that causes Legionnaires’ disease, a serious and potentially fatal pneumonia. Previous studies have shown that members of this species undergo a process called recombination, whereby DNA is imported from another bacterial cell into the recipient genome. The imported DNA can either replace an equivalent segment of the genome (homologous recombination) or can comprise novel genes that are new to the recipient genome (non-homologous recombination). Whilst recombination plays an undoubtedly important role in *L*. *pneumophila* evolution, accounting for more than 96% of the diversity observed within some lineages, little is known about its biological impact. In this study, we performed a detailed characterisation of the dynamics and effect of homologous recombination on *L*. *pneumophila* evolution in six clinically important lineages of *L*. *pneumophila*. We identified “hotspot” regions of the genome in which an excess of homologous recombination events was observed, which provided important clues to the selection pressures faced by *L*. *pneumophila*. By determining the donors of the recombined regions, we also revealed that recombination has occurred most frequently between isolates from the same clade, but also occurred between isolates from different major clades. This demonstrates the possibility of new adaptations arising in one lineage and being transferred to another distantly related lineage, which we predict has been an important factor in the emergence of several major disease-causing strains from diverse genomic backgrounds.

## Introduction

While all bacteria reproduce clonally, some also import DNA from other organisms into their chromosomes through processes such as recombination or horizontal gene transfer. The imported DNA can either replace a homologous segment of the genome (homologous recombination) or comprise novel genes that are new to the recipient genome (non-homologous recombination). The former results in the replacement of genes with alternative allelic variants and requires the DNA to be highly similar, and possibly identical, at both ends of the fragment [1]. For this reason, homologous recombination usually occurs between closely related bacteria.

The importance of recombination in bacterial evolution first became clear through the analysis of multi-locus sequence typing (MLST) data, which showed that phylogenetic trees constructed from individual MLST genes were often incongruent [2]. These analyses also predicted that the rate of homologous recombination varies considerably between different species [3]. There are a number of hypotheses regarding why bacteria engage in homologous recombination [4]. One explanation is that recombination is used as a mechanism by which DNA damage can be repaired using foreign DNA as a template [5]. Another is that it is a side effect of DNA uptake for use as an energy source or for DNA synthesis from nucleotide precursors [6]. Third, the ability of recombination events to remove deleterious mutations and rapidly introduce combinations of advantageous mutations could mean it increases the efficiency of natural selection and is selectively maintained [7]. Finally, a recent study has also suggested that bacteria use recombination to delete selfish mobile genetic elements from their genomes [8].

In recent years, the availability of whole genome sequence (WGS) data from multiple closely related bacterial isolates has enabled homologous recombination to be studied in great detail in species such as *Streptococcus pneumoniae* [9, 10], *Chlamydia trachomatis* [11] and *Neisseria meningitidis* [12]. These studies have confirmed that homologous recombination plays an important role in the evolution and adaptation of important bacterial pathogens, for example by facilitating vaccine escape [9] and antibiotic resistance [10] in *S*. *pneumoniae*.

*Legionella pneumophila* is an environmental bacterium that parasitizes and replicates inside protozoa in freshwater and soil habitats [13]. It also now colonises man-made water systems from which humans can become infected via inhalation of contaminated aerosols [14]. Infection can cause Legionnaires’ disease, a serious and potentially fatal pneumonia [15]. *L*. *pneumophila* was first reported to have a clonal population structure based on multi-locus enzyme electrophoresis (MLEE) analysis [16]. However, the three primary mechanisms of bacterial recombination (conjugation, transduction and transformation) have since all been described in *L*. *pneumophila* [17–19], and thus it was unsurprising when later studies reported its occurrence. Indeed, an early genomic study of the first sequenced genomes of *L*. *pneumophila* showed that recombination events are frequent and suggested that it can involve large chromosomal fragments of over 200kb [20]. More recently, larger genomic studies have demonstrated that >95% of single nucleotide polymorphisms (SNPs) detected within some lineages have been imported via recombination [21, 22]. The occurrence of recombination within *L*. *pneumophila* populations has also led to the existence of multiple genetic subtypes within single outbreaks [21, 23]. However, despite its major role in *L*. *pneumophila* evolution, the relative frequency and biological effects of recombination, such as its impact on virulence or adaptation of *L*. *pneumophila* to new niches, remain poorly understood.

Here, we disentangled events arising from homologous and non-homologous recombination in six major disease-associated sequence types (STs) of *L*. *pneumophila*, and subsequently performed a detailed characterisation of the dynamics and biological impact of homologous recombination on *L*. *pneumophila* evolution. Our findings provide novel insights into the selection pressures of *L*. *pneumophila* and the dynamics of genomic flux within the species.

## Results & discussion

### The contribution of homologous recombination to *L*. *pneumophila* diversity

To investigate the relative contribution of homologous recombination to diversity in each of six major disease-associated lineages of *L*. *pneumophila* (STs 1, 23, 37, 42, 62 and 578), sequence reads from isolates (*n* = 291) (**S1 Table**) were first mapped to a reference genome of the same ST (**Table 1**). Isolates belonging to STs that have previously been shown to be derived from the ST1 lineage were also included with ST1 isolates [22]. Gubbins was used to detect recombined regions in each of the six genome alignments [24]. This tool uses increased SNP density on branches of a phylogenetic tree as a marker, and is well suited to these six lineages that contain low background diversity. However, it should be noted that recombination between highly similar isolates may be missed, potentially leading to an underestimation of the recombination rate. In our previous study using four of these alignments with the same, or largely the same, isolates [22], this programme showed high concordance with another recombination detection tool, BRATNextGen [25]. Detection of recombined regions using BRATNextGen is based on sequence similarity rather than SNP density, and thus the high concordance between the two different approaches provides confidence in our predicted regions. As previously reported [21, 22], over 96% of SNPs in STs 1, 23, 37, 62 and 578 were predicted to be derived from recombination events. Furthermore, 99% of SNPs in the ST42 lineage were found in recombined regions in the present study. Thus, in all six lineages the proportion of SNPs derived from recombination is higher than that reported for the highly recombinogenic *S*. *pneumoniae* PMEN1 lineage (88%) [9] and between *N*. *meningitidis* ST60 strains (94.25%) [12]. The number of vertically inherited SNPs that remained after the removal of recombined regions in each of the six *L*. *pneumophila* lineages ranged from 94 (ST42) to 1,006 (ST1) (**Table 1**).

**Table 1:** Number of SNPs detected before and after the removal of recombined regions within six major disease-associated STs.

ST	Number of isolates	Mapping reference	Total number of SNPs*	Number of vertically inherited SNPs only (% of total)*
ST1 (and ST1-derived)	81	Paris [26]	73,044	1,006 (1.4%)
ST23	42	EUL 28 [27]	44,886	166 (0.4%)
ST37	72	EUL 165 [27]	17,776	476 (2.7%)
ST42	15	EUL 120 [27]	9,256	94 (1.0%)
ST62	35	H044120014 [27]	47,684	312 (0.7%)
ST578	46	Alcoy [28]	3,678	119 (3.2%)

*The number of total and vertically inherited SNPs reported in the ST37 and ST62 lineages deviate slightly from those previously reported [22], despite the same isolates and sequence data being used, which can be explained by the use of different reference genomes.

**Fig 1:**
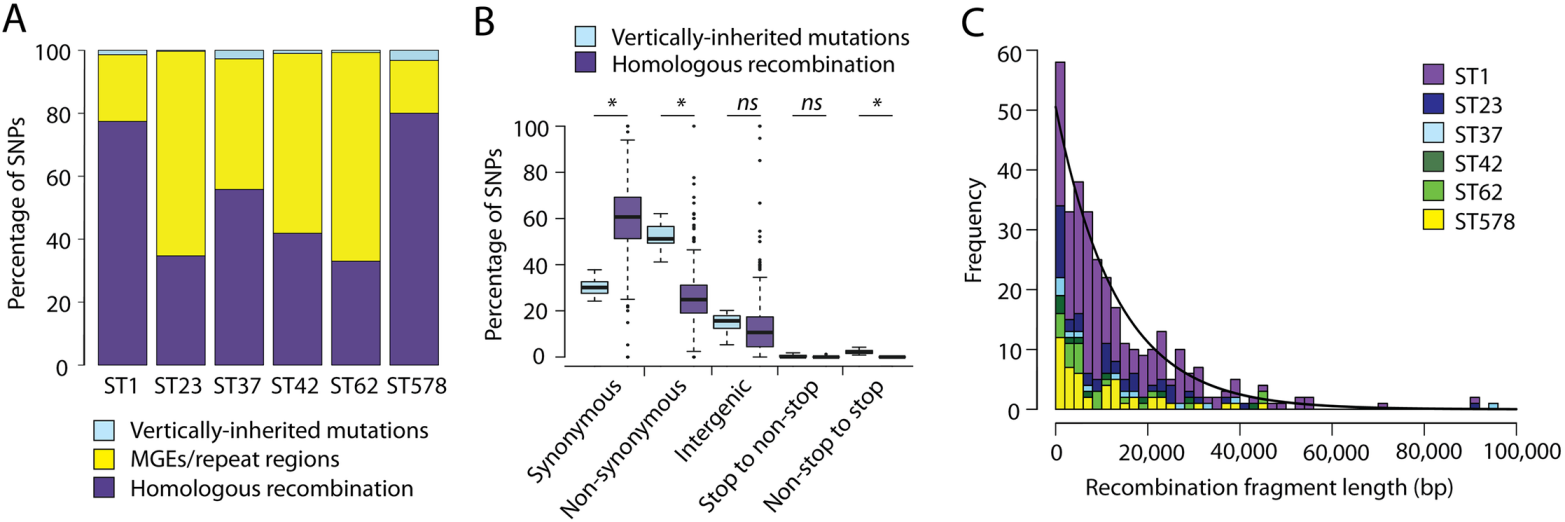
A) The percentage of SNPs in each of six major disease-associated STs that are derived from vertically-inherited mutations, homologous recombination events, or found within regions comprising mobile genetic elements (MGEs) (i.e. non-homologous recombination) or repeat regions. B) Boxplots showing the percentage of SNPs per branch, derived from either vertically-inherited mutations or homologous recombination that are synonymous, non-synonymous, intergenic, or result in a change from a stop to non-stop codon or a non-stop to stop codon. C) Distribution showing the size of detected homologous recombination regions in the six STs. An exponential decay curve (black line) is fitted and the rate of decay is 7.52 x 10^−5^ bp^-1^.

Any regions detected by Gubbins that overlapped with either predicted mobile genetic elements (MGEs) or repeat regions (**S2 Table**) were subsequently excluded, in order to determine the sole contribution of homologous recombination to *L*. *pneumophila* diversity (**Table 2**). We found that between 33% (ST62) and 80% (ST578) of all SNPs were predicted to be in regions derived from homologous recombination events (**Fig 1A**). However, the average length of each individual genome affected by this process varied between just 1.2% (ST42/578) and 3.9% (ST1) (**Table 2**). It should be noted that the number of SNPs predicted to be from homologous recombination might be slightly over-estimated (and the number of *de novo* mutations slightly under-estimated) since *de novo* mutations may have occurred on top of recombined regions. However, the error should be no more than 1.2–3.9%, in proportion with the average length of genome affected by homologous recombination events. Furthermore, detectability of homologous recombination events could also be affected by lineage diversity (i.e. events may be more difficult to detect on longer tree branches where background SNP density is higher). However, because the number of SNPs associated with recombining regions is much higher compared with the background vertically inherited SNPs in all lineages, we think that any effect will be minimal.

**Table 2:** Contribution of homologous recombination to the diversity of six major disease-associated STs.

ST	Number of homologous recombination events	Number of SNPs in homologous recombination regions per vertically inherited SNP (r/m ratio)	Number of homologous recombination events per vertically inherited SNP (ρ/θ ratio)	Mean length (and %) of each individual genome affected by homologous recombination (bp)	Total length (and %) of the reference genome affected by homologous recombination across all isolates (bp)
ST1 (and ST1-derived)	198	56.2	0.20	135,208 (3.9%)	1,430,288 (40.8%)
ST23	44	93.8	0.27	51,242 (1.5%)	520,584 (14.8%)
ST37	13	20.8	0.03	105,051 (3.0%)	251,988 (7.3%)
ST42	11	41.3	0.12	41,747 (1.2%)	120,545 (3.5%)
ST62	48	50.5	0.15	66,559 (1.9%)	456,451 (12.9%)
ST578	23	24.6	0.19	42,138 (1.2%)	204,114 (5.8%)

In each of the six lineages, the relative number of homologous recombination events to vertically inherited mutations (ρ/θ ratio) was calculated per branch for each phylogenetic tree (**S1 Fig**) and also for each lineage as a whole. The overall ρ/θ ratio for each lineage ranged from 0.03 (ST37) to 0.27 (ST23), indicating that recombination events have occurred less frequently than vertically inherited mutations in all six lineages, despite bringing in between 20.8 (ST37) and 93.8 (ST23) times as many SNPs (**Table 2**). A similar ρ/θ ratio of 0.124 was reported in previous analysis of 25 diverse *L*. *pneumophila* genomes as inferred by an alternative recombination detection algorithm, ClonalFrame [29]. The distribution of per-branch ρ/θ ratios also differ significantly between lineages (Kruskal-Wallis test, p<0.05), highlighting different rates of recombination in the six major disease-associated STs. These differences could indicate variation in the biological niches of these different lineages, about which very little is currently understood, and/or the availability of recombination opportunities.

To determine the relative impact of vertically inherited mutations and homologous recombination events on the coding sequence, the types of changes caused by the two processes were analysed (**Fig 1B**). Vertically inherited mutations have resulted in approximately twice as many non-synonymous SNPs than synonymous SNPs, a result that is expected by chance when mutations occur at random in the genome and before selection has time to act on all but the most deleterious mutations. Interestingly though, the results are reversed for homologous recombination events, which resulted mostly in synonymous mutations. However, this observation is not unexpected given that variants in sequences that are horizontally transferred between different lineages will have been subjected to a longer period of evolution and selection, which has purged harmful, non-synonymous mutations. The same phenomenon has also been observed in a previous study by Castillo-Ramirez *et al*. (2011) [30]. Furthermore, fewer SNPs that result in a stop codon were brought in by homologous recombination events than by vertically inherited mutations, which can also be explained by this process.

The lengths of the recombined regions have an approximately exponential distribution (rate of decay = 7.52 x 10^−5^ bp^-1^), with the majority of events being small (<10,000bp) and large events occurring relatively infrequently (**Fig 1C**). The median recombination fragment length in each of the six lineages ranged from 5,613bp (ST578) to 12,757bp (ST37), while the largest predicted region is 94,790bp (ST37). Large recombination segments have also been found in other species, such as *Clostridium difficile* [31], *Streptococcus agalactiae* [32] and *Streptococcus pneumoniae* [33]. In the latter, a similar distribution of fragment sizes as the one described here for *L*. *pneumophila* was also reported, suggesting that transformation is optimised for exchanging short sequences rather than large features such as complete operons [33]. This scenario could be favoured as it allows for larger numbers of potentially advantageous allele combinations to be tested.

### Hotspots of homologous recombination in *L*. *pneumophila*

**Fig 2:**
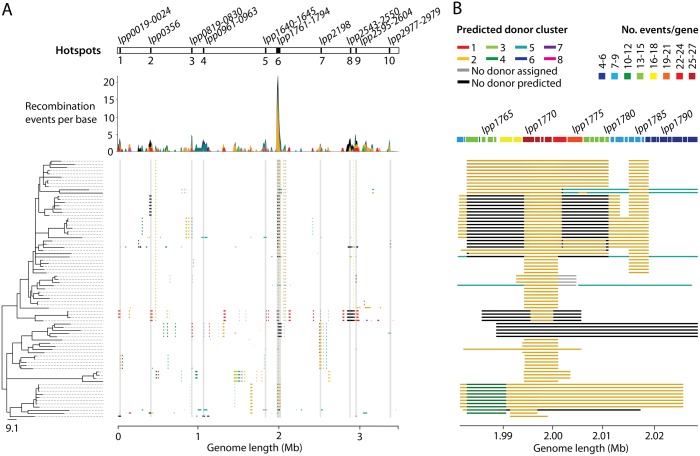
A) Homologous recombination events detected in the ST1 lineage. A phylogenetic tree, constructed using only vertically inherited mutations, is shown on the left and the scale indicates the number of SNPs. Bootstrap values are provided in **S2 Fig**. Homologous recombination events are shown by blocks adjacent to the tree, which are coloured according to the BAPS cluster from which they are predicted to have been derived (see key at the top left of panel B). The plot above shows the number of recombination events that have affected each base in the genome using a stacked visualisation to also indicate the number of events derived from different clusters. The ten genomic regions identified as recombination hotspots are marked at the top of the plot. B) A zoomed-in illustration of hotspot 6 in the ST1 lineage, which ranges from *lpp1761* to *lpp1794*. As in A, the homologous recombination events are displayed as blocks and coloured according to the BAPS cluster from which they are predicted to be derived. The genes shown at the top of the figure that make up hotspot 6 are coloured by the number of times that they have been affected by a homologous recombination event (see key at the top right).

Next, we determined whether there are any genomic regions that are associated with a higher number of homologous recombination events, which could reveal genes that are under diversifying selection pressure. We thus calculated the number of events predicted by Gubbins that overlap with each gene with respect to the reference genomes of the six disease-associated STs. A total of 32 hotspot regions were defined (see Materials & Methods), including at least one in all six disease-associated STs (**S3 Table**). A total of 10 hotspot regions were identified in the ST1 lineage and, remarkably, one region contained genes that are predicted to have been involved in up to 27 recombination events (**Fig 2A**). By contrast, in the other five STs, the highest number of events affecting genes ranged from 2 (ST37/ST578) to 4 (ST42/ST62). We acknowledge that the number of recombination events detected per gene, particularly in hotspot regions, could be slightly underestimated due to the possible occurrence of overlapping or nested recombined regions imported on the same branch of the phylogenetic tree. Gubbins is likely to predict these as single rather than multiple events, and genomic regions with a higher number of recombination events could be disproportionately affected. Nevertheless, the identification of hotspot regions provides good evidence that the effect of recombination in *L*. *pneumophila* is to increase the genetic diversity available for natural selection to work on, and that this diversifying selection acts non-randomly on the genome.

#### The prominent ST1 recombination hotspot

The most prominent hotspot identified in the ST1 lineage that contains genes involved in up to 27 recombination events is a 47,174bp region that ranges from *lpp1761* to *lpp1794* in the Paris (ST1) genome (**Fig 2B**). The gene in this region that is predicted to have been involved in 27 events is *hemB*/*lpp1771*, a porphobilinogen synthase (delta-aminolevulinic acid dehydratase), which is an enzyme involved in the biosynthesis of tetrapyrroles. The surrounding genes, *lpp1770*, *lpp1772* and *lpp1773*, are also predicted to have been involved in 25 recombination events each. To further confirm the highly recombinogenic nature of this region, we also analysed it with a recently published recombination detection tool, fastGEAR [34], which estimates lineages present in a given alignment and recombinations between the lineages or from external origins. The fastGEAR algorithm was run separately on all 34 genes in the hotspot (*lpp1761*-*lpp1794*), as well as 10 genes upstream and downstream, using a complete alignment of all 536 *L*. *pneumophila* genomes used in this study (rather than ST-specific alignments as used with Gubbins). In concordance with Gubbins, it predicted that the genes with the highest numbers of recombination events imported into the ST1 lineage in this region are *lpp1770* and *lpp1771* (**S3 Fig** & **S4 Table**). Furthermore, of the 46 recombination events in the entire hotspot region (*lpp1761*-*lpp1794*) that fastGEAR predicts to have been imported into the six lineages of interest, 29 (63.0%) have affected ST1 isolates, further confirming the prominence of this hotspot in the ST1 lineage with respect to the other STs (**S4 Fig** & **S4 Table**).

Intriguingly, there is no obvious reason why the metabolic gene *hemB/lpp1771* (predicted by Gubbins to be the most recombinogenic) should be under strong diversifying selection. However, while the two immediate flanking genes (*lpp1770* and *lpp1772*) both encode “hypothetical proteins”, *lpp1773*, which has been involved in 25 recombination events, has been shown to encode an outer membrane protein of *L*. *pneumophila* in a previous study [35] and has high similarity to the *fadL* gene, conserved across many bacterial species. In *Escherichia coli*, the FadL protein is essential for uptake of long-chain fatty acids and also acts as a phage receptor [36] while in *Salmonella paratyphi*, it has been demonstrated to be highly immunogenic [37]. In *L*. *pneumophila*, FadL could be under a high selection pressure to vary in order to either escape protozoan predation (different protozoan species may have different specificity to outer membrane structures), to adapt to different hosts or to cope with an immune response during infection of host cells. However, since protozoa do not have an adaptive immune response, the latter possibility is unlikely unless more complex organisms (e.g. humans) are also part of the infection cycle. While human-to-human transmission of *L*. *pneumophila* has been demonstrated only once [38], the recent and independent emergence of several major disease-associated STs has also raised the possibility of human infection being part of the propagation cycle [22]. However, another possible explanation for the high recombination frequency could be that genes within this region have been frequently gained and lost through evolutionary time.

Interestingly, a *fadL*-like gene (*ST62_00760*; *lpp0762*) is also found within a recombination hotspot in the ST62 lineage, where it is involved in two recombination events (as predicted by Gubbins), although it is found in a different part of the genome to the ST1 hotspot region. Furthermore, a smaller 6,778bp hotspot region in the ST23 lineage (*ST23_01779-ST23_01781; lpp1768*-*lpp1770*) overlaps with the ST1 hotspot region. However, the region in the ST23 lineage centres on the gene, *ST23_01780*/*lpp1769*, which is involved in three recombination events and encodes the outer membrane protein assembly factor, BamA. The same result was also found using fastGEAR, which detected recombination in the ST23 lineage in only the *ST23_01778*/*lpp1767*, *ST23_01780*/*lpp1769* and *ST23_01781*/*lpp1770* genes (of the 54 genes tested) (**S4 Table**). Interestingly, *lpp1769* is involved in “just” 18 recombination events in the ST1 lineage, compared with *lpp1771* that is involved in 27 (as predicted by Gubbins). Further studies, perhaps involving a larger number of isolates, would be useful to confirm the gene(s) that are driving these hotspots and to determine whether the prominent hotspot region in the ST1 lineage is also an important hotspot region in other lineages, or whether it represents a unique selection pressure in ST1 isolates.

#### The LPS locus

The second most prominent hotspot in the ST1 lineage is a 13,607bp region that ranges from *lpp0819* to *lpp0830* in the Paris genome, and which contains genes affected by up to 7 recombination events (**S5 Fig**). This hotspot is fully contained within the lipopolysaccharide (LPS) locus, which spans a region from *lpp0814* to *lpp0843*. Many of the genes in this hotspot region have been implicated in LPS core oligosaccharide biosynthesis, including those belonging to the *rml* family, and O-antigen biosynthesis, including *neuA*, *neuB*, *neuC*, *wecA*, *wzt* and *wzm* [39]. Interestingly, the genes affected by the highest number of recombination events are *wecA* but also *lpp0829a-c*, which are annotated as pseudogenes in the original annotation of the Paris genome [26]. All three genes encode “hypothetical proteins” although *lpp0829a* has a signal peptide and thus may be secreted, while *lpp0829b* has a pectin lyase fold, a motif which has also been found in genes belonging to *Legionella longbeachae* and is thought to degrade the pectic components of plant cell walls. Furthermore, the ST62 lineage also has two genes from the LPS locus that are in hotspot regions. It is unsurprising that the LPS locus was found as a recombination hotspot since LPS has previously been shown to be the major immunodominant antigen of *L*. *pneumophila* in the laboratory [40, 41]. However, the specific reasons that variability in the LPS is being selected for could be any of those already described for FadL. Horizontal exchange of the LPS locus also explains a previous observation that serogroup 1 isolates can have diverse genomic backgrounds, and that serogroups often do not correlate with overall genomic relatedness [42].

#### Outer membrane proteins

Across all six disease-associated STs, outer membrane proteins are commonly found within recombination hotspot regions. Excluding those mentioned already (i.e. FadL and BamA), these include TolC or TolC-like proteins, involved in two recombination events in the ST23 lineage (*ST23_00709*/*lpp0754*) and also in two events in the ST578 lineage (*lpa_01256*/*lpp0889)*, and which have been implicated in the virulence of *L*. *pneumophila* [43]. Other outer membrane proteins found within recombination hotspots include *ST23_00628*/*lpp0671* in ST23 and *ST37_01207*/*lpp1191* in ST37 [35]. Furthermore, the *lpp0961* gene, involved in four recombination events in the ST1 lineage, encodes a protein homologous to AsmA in *E*. *coli*, which is involved in the assembly of outer membrane proteins. Thus, of the many outer membrane proteins likely expressed on the surface of *L*. *pneumophila*, these results provide clues as to which are being selected for variation and are therefore part of dynamic environmental interactions.

#### Dot/Icm effectors

A number of genes encoding putative or confirmed Dot/Icm effectors are also found within recombination hotspots across the different lineages. Dot/Icm effectors, which are secreted by a type IVB secretion system of *L*. *pneumophila* and of which there are over 300 described, manipulate a wide range of host cell processes and are essential to *L*. *pneumophila* pathogenesis [44]. Those found in hotspots include *lpp0356* and *lpp2546* in ST1, which encode an ankyrin repeat-containing protein originally found only in the Paris genome [26] and the SdbB effector, respectively. A further three ankyrin repeat-containing effector genes were identified within ST23 hotspots including *ST23_02606/lpp2517* (encoding LegA14), *ST23_00705/lpp0750* (encoding LegA8) and *ST23_00415/lpp0469* (encoding LegA7). The first described Dot/Icm effector, RalF, encoded by *ST23_01938*/*lpp1932*, was also found within a ST23 hotspot and predicted to have been involved in two recombination events. It will be intriguing to decipher whether variation is being selected for within these effectors in order to take advantage of a wide variety of host cells, or to counter defence strategies by protozoan hosts. Larger sets of genomic data would be useful to confirm the existence of these hotspots and further explore differences between lineages, which could suggest differences in hosts and infection strategies.

#### Enhanced entry proteins

Finally, while only 11 homologous recombination events were detected within the ST42 lineage, genes within one 14,572bp region have been affected by up to four recombination events. The hotspot region is centred on *ST42_02565*/*lpp2693*, which encodes the enhanced entry protein EnhB, but also includes the other enhanced entry proteins EnhA and EnhC. While little is known about EnhB, EnhC has been shown to be important for entry into host cells [45] and to facilitate intracellular growth of *L*. *pneumophila* by evading immune recognition by the pattern recognition receptor (PRR), Nod1, in macrophages [46]. Further studies are required to understand why variability within the enhanced entry proteins might be advantageous, and also why these genes were found in a hotspot in the ST42 lineage and not others.

### Inference of recombination donors

**Fig 3:**
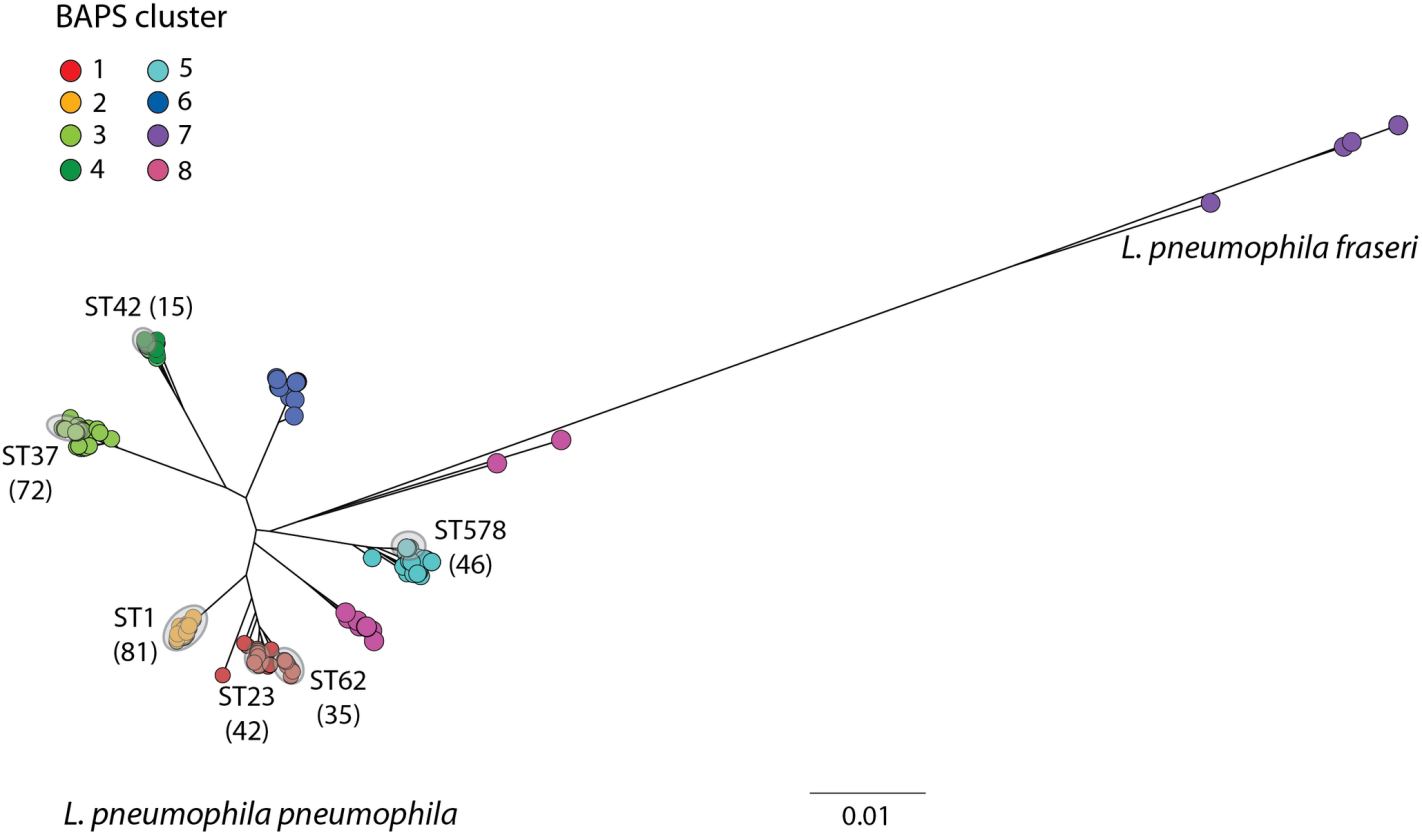
Maximum likelihood tree of 536 *L*. *pneumophila* isolates generated by mapping sequence reads to the Paris (ST1) reference genome. Isolates are coloured by BAPS cluster. Grey circles also highlight the position of the six major disease-associated STs and the number of isolates belonging to each ST is indicated in brackets (ST1-derived isolates are here considered as ST1). The scale shows the number of SNPs per site. Bootstrap values, based on 100 resamples, are shown for the major nodes of the tree.

To predict the origin of the homologous recombination regions, 536 *L*. *pneumophila* genomes were first divided into clusters using hierBAPS [47], which were mapped onto a phylogenetic tree (**Fig 3**). The genomes comprise those belonging to isolates from the six major disease-associated STs (*n* = 291) (**S1 Table**) and others from a large, species-wide collection (*n* = 245) (**S5 Table**). Eight BAPS clusters were identified, seven of which comprised isolates from the *L*. *pneumophila pneumophila* subspecies (BAPS clusters 1–6, 8), and one with isolates from *L*. *pneumophila fraseri* (BAPS cluster 7). Of the 318 homologous recombination events greater than 500bp predicted in the six major disease-associated lineages, potential donors were predicted for 292 (91.8%) (see Materials & Methods). Many of the hits were almost perfect matches with 122 (41.8%) of the fragments having over 99.9% nucleotide identity, and 155 (53.1%) having hits that covered the full length of the recombination fragment (**S6 Fig**).

**Fig 4:**
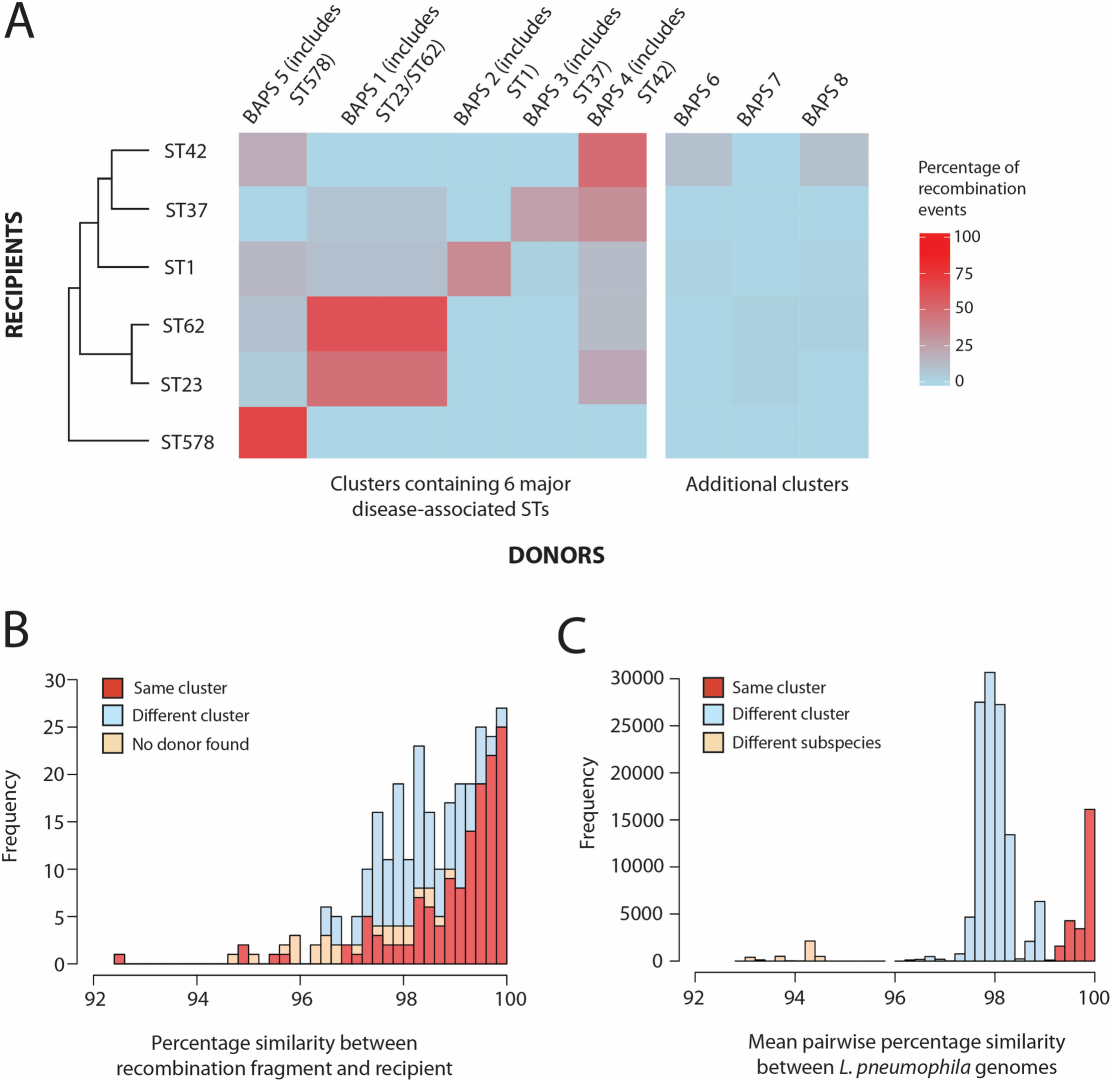
A) Heat-map showing the percentage of recombination events detected in each of the six lineages (STs 1, 23, 37, 42, 62 and 578) that are derived from each of the eight BAPS clusters. The six STs are shown in the left dendrogram constructed using hierarchical clustering and based on the similarity of the predicted recombination donor lineages. The BAPS clusters are ordered from left to right based on the ordering of the six STs in the dendrogram. The column representing BAPS cluster 1, which contains both ST23 and ST62, is given twice the width as the other columns. The three BAPS clusters (6–8) that do not contain one of the six major disease-associated STs are shown on the right. B) Distribution of the percentage nucleotide similarities between the imported recombination fragments and the recipient sequence in all of the six STs, with the events categorised as derived from the same or different BAPS clusters or with no donor lineage identified. C) Distribution of pairwise nucleotide similarities across the genome amongst the 536 *L*. *pneumophila* isolates used in this study.

The number of homologous recombination events in each of the six major disease-associated lineages that were predicted to be derived from each of the eight BAPS clusters were calculated and visualised in a heat plot (**Fig 4A**). Any events with equally good hits (i.e. with the same nucleotide similarity and fragment length covered) to isolates in more than one BAPS cluster were discarded for this analysis (“No donor assigned”). The heat plot illustrates that, in five of the six STs, recombination donors most often belonged to the same BAPS cluster as the recipient. This is an expected finding since homologous recombination requires high, or even perfect, sequence similarity between the donor and recipient at both ends of the recombination fragment [1], a scenario which is more likely between closely-related bacteria. The exception is ST37 in which the highest number of recombination fragments is derived from BAPS cluster 4, although its own cluster (BAPS cluster 3) accounted for the second highest number. However, all STs, with the exception of ST578, are also predicted to have acquired recombination fragments from clusters other than their own, demonstrating the occurrence of homologous recombination between major clusters of the *L*. *pneumophila pneumophila* subspecies. This result is confirmed by the fastGEAR analysis of the prominent ST1 hotspot region, which demonstrates the sharing of alleles between different BAPS clusters (**S4 Fig**). Overall, the finding suggests that different clades have at least partially shared the same ecological niche and perhaps even the same individual host cells in which recombination may have occurred. Importantly, this freedom of genomic exchange has provided potential opportunities for new adaptations to be shared freely amongst different clusters, which we hypothesise has been an important factor in the recent emergence of multiple major disease-associated STs from diverse genomic backgrounds [22]. Interestingly, some BAPS clusters act frequently as donors to other clusters (e.g. BAPS clusters 4 and 5), while others hardly donate except to isolates of their own cluster (e.g. BAPS clusters 2 and 3) (**Fig 4A**). Similar patterns whereby different lineages donate and receive DNA at different rates have also been observed in other species such as *S*. *pneumoniae* [10], *C*. *trachomatis* [11] and *E*. *coli* [48].

Furthermore, just two events (one each in ST23 and ST62) are derived from the *L*. *pneumophila fraseri* subspecies (BAPS cluster 7). Given that this lineage shares less than 95% nucleotide identity with the *L*. *p*. *pneumophila* subspecies, this was not an unexpected finding, given the high level of similarity required for homologous recombination. It could be that these two subspecies have gradually diverged due to differing ecologies, and that eventually they may become different species that are fully incapable of exchange *via* homologous recombination.

For all homologous recombination events detected in the six STs, the nucleotide identity between the imported fragment and the recipient genome that was replaced by the fragment was calculated (**Fig 4B**). This was to investigate the divergence levels between recombining bacteria, but it also provided a means of verifying our predictions of the recombination donors. This analysis showed that 70% of homologous recombination events occurred between closely related isolates with >98% nucleotide similarity in the affected region, which agrees with our previous finding that most fragments are derived from the same BAPS cluster as the recipient. Interestingly, two peaks can be observed at ~98% identity and ~99.5–100% identity. These levels of divergence correspond to the nucleotide similarity observed between isolates belonging to different clusters or the same cluster, respectively (**Fig 4C**), and thus they represent recombination between and within clusters. It is also interesting to note that the distribution of pairwise nucleotide similarities of genomes from different clusters has a major peak around ~98% (**Fig 4C**), which aligns with previous findings that homologous recombination tends make clusters equidistant from each other [49, 50].

Recombination hotspot regions were next re-analysed to investigate whether the hotspots were driven by recombination events from the same or different BAPS clusters. The analysis focused on the ST1 lineage, which was previously found to contain the highest number of recombination events and the most prominent hotspots. The most notable hotspot region (hotspot 6), which was found to contain genes involved in up to 27 recombination events, was found to be driven mostly by recombination regions derived from the same BAPS cluster to which ST1 belongs (BAPS cluster 2) (**S7 Fig**). However, a small number of recombination events that are predicted to be from BAPS cluster 5 were also observed in this region. While the analysis of this region using fastGEAR is not directly comparable to the results using Gubbins, it does also suggest that the recombined regions have been imported from both the same and different BAPS clusters (**S4 Fig**). Meanwhile, while some of the recombination events affecting the LPS locus (hotspot 3) could not be assigned a donor, others were derived from BAPS clusters 1, 2 and 5, suggesting that high diversity in this region may be especially important. Hotspot 4 appears to be driven by recombination events from BAPS clusters 5, 6 and 8 and contains no events derived from BAPS cluster 2 (to which ST1 belongs). However, the small number of events with predicted donors in most of these hotspots limits the conclusions that can be made.

**Fig 5:**
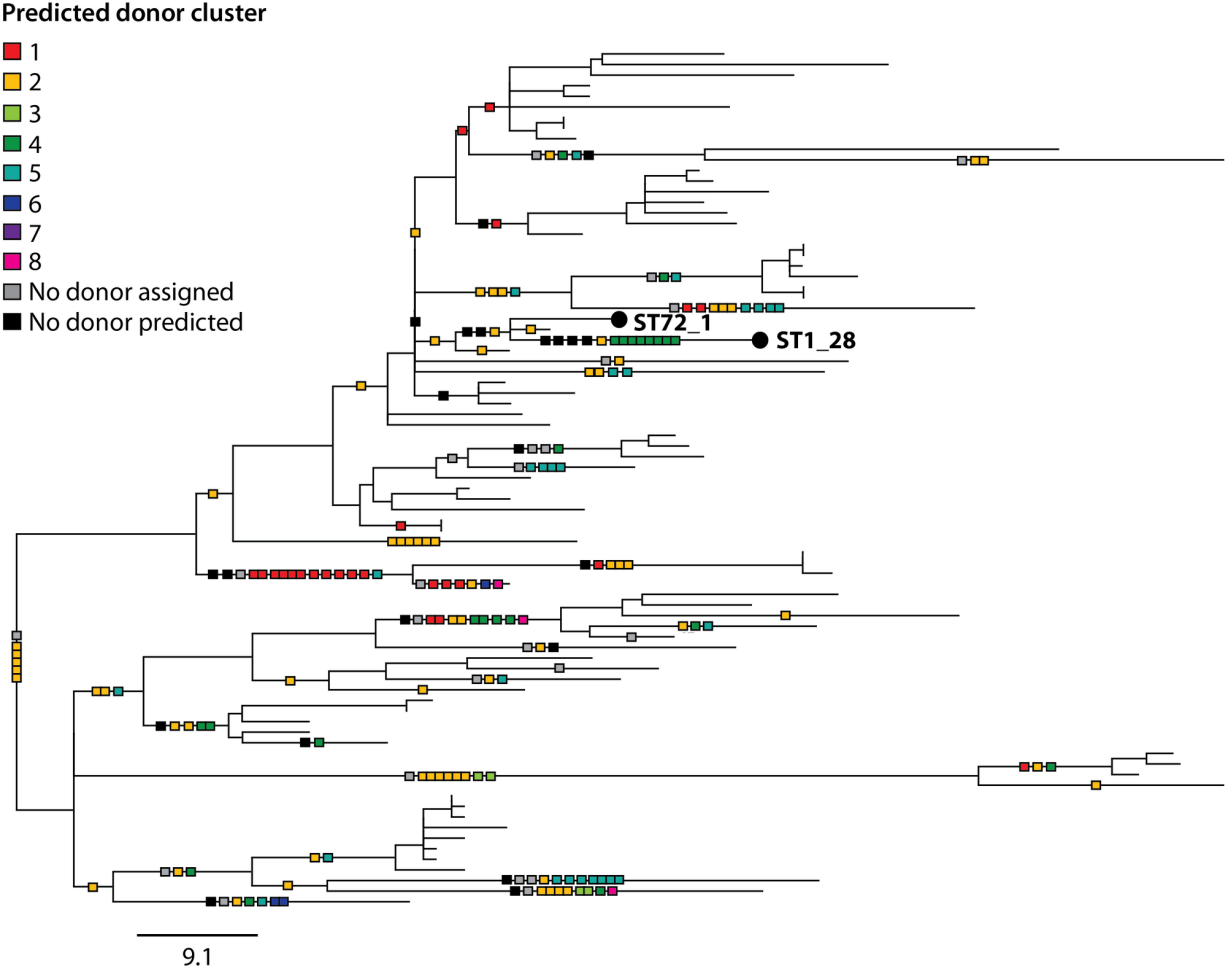
Maximum likelihood tree of 81 ST1 (or ST1-derived) isolates constructed using only vertically-inherited SNPs. Predicted homologous recombination events are mapped onto the phylogeny (shown by squares on the branches) and coloured according to the BAPS cluster from which they are predicted to have been derived. Squares representing events with the same predicted donor at the isolate level and that have occurred on the same branches are joined together, and possibly represent multi-fragment recombination. ST1_28 and ST72_1, which are referred to in the text and in **S8 Fig**, are labelled. The scale bar indicates the number of SNPs.

Finally, the homologous recombination events that were predicted within the ST1 lineage were mapped onto the phylogenetic tree together with information regarding their predicted origin (**Fig 5**). This was to search for evidence of multi-fragment recombination, a process in which multiple non-contiguous segments that originate from the same molecule of DNA are imported into a recipient genome in a single episode of recombination. This process is well documented in *S*. *pneumoniae* [33, 51, 52]. Since the recombining fragments are non-contiguous, Gubbins will detect these as separate events although the events should be predicted to have occurred on the same branch and have the same predicted donor. Indeed, we found some evidence for the occurrence of this process in *L*. *pneumophila*, since many events with the same predicted donor, down to the BAPS cluster level and even the individual isolate level, are co-localised on branches (**Fig 5**). For example, 8 recombinant regions distributed throughout the chromosome that occurred on the terminal branch leading to ST1_28 are predicted to have originated from BAPS cluster 4, and more specifically, a strain (or multiple strains) closely related to EUL 25 (ST44) (**S8 Fig**). Furthermore, some of these imported regions also share very similar SNP densities with respect to EUL 25 (i.e. 5 events have SNP densities from 0–0.06% and 3 events have SNP densities from 0.28–0.33%), reinforcing the possibility that some of these recombining fragments could have been acquired from the same donor in the same event. However, it could also be that the recombining isolates have shared a common niche for a prolonged period of time, and that multiple independent recombination events have occurred during this time. Thus, further experimental studies will be required to confirm the occurrence of this process in *L*. *pneumophila*.

In summary, this study has demonstrated a major role for homologous recombination in shaping the population structure and evolution of *L*. *pneumophila*, and provided detailed insights into recombination dynamics within the species. We predict that homologous recombination has played a critical role in the emergence of this environmental bacterium as an important human pathogen and suggest that future studies are required to further delineate the role of homologous recombination in the virulence and adaptation of *L*. *pneumophila* to modern, man-made environments.

## Materials & methods

### Bacterial isolates

*L*. *pneumophila* isolates belonging to six major disease-associated lineages are primarily used in this study (*n* = 291), all of which have been previously sequenced [21, 22, 27, 53–55]. These include 81 ST1 (or ST1-derived), 42 ST23, 72 ST37, 15 ST42, 35 ST62, and 46 ST578 isolates (**S1 Table**). A further 245 *L*. *pneumophila* isolates, which belong to a range of STs, were also used in the inference of recombination donors (**S5 Table**). WGS data from all but five of these isolates have been published [20–22, 26–28, 55–60]. Importantly, these include a set of genomes that were selected for sequencing using sequence-based typing (SBT) data, analogous to MLST, with the aim of encompassing as much of the species diversity as possible [55]. Accession numbers or references for all genomes are provided in **S1 Table and S5 Table**.

### Reference genomes

Sequence reads from isolates belonging to each of the six disease-associated STs (1, 23, 37, 42, 62 and 578) were mapped to a reference genome of the same ST to enable each lineage to be studied at a high resolution. The complete genomes of Paris [26] and Alcoy [28] were used for ST1 and ST578, and reference genomes previously generated using a Pacific Biosciences (PacBio) RSII sequencer were used for STs 23 (EUL 28), 37 (EUL 165), 42 (EUL 120) and 62 (H044120014) [27]. All six reference genomes were annotated using an in-house pipeline at the Wellcome Trust Sanger Institute (WTSI), which uses Prokka [61]. The four annotated reference genomes obtained using PacBio sequencing are available from the European Nucleotide Archive under the accession numbers GCA_900119755.1 (EUL 28), GCA_900119775.1 (EUL 165), GCA_900119785.1 (EUL 120) and GCA_900119765.1 (H044120014). Repetitive regions over 100bp were detected in the six reference genomes using repeat-match from MUMmer v3.0 [62] (**S2 Table**).

### Whole genome sequencing, mapping and SNP calling

All processing and sequencing of genomic DNA from the five newly sequenced isolates was performed by the core sequencing facility at the WTSI. Paired end libraries were created as described previously [63] and samples were sequenced using the Illumina HiSeq platform and paired-end reads of 100 bases. Sequence reads of all isolates belonging to the six major disease-associated STs under study were mapped to the appropriate reference genome of the same ST using SMALT v0.7.4 (available at: http://www.sanger.ac.uk/science/tools/smalt-0). All isolates used in the study (*n* = 536) were also mapped to the Paris (ST1) reference genome [26] in order to study the species-wide phylogenetic structure. An in-house pipeline at the WTSI was used to call bases and identify SNPs as previously described [64].

### *De novo* assembly

All assemblies were produced from the Illumina data using a pipeline developed by the Pathogen Informatics team at the WTSI. This firstly uses Velvet Optimiser (https://github.com/tseemann/VelvetOptimiser) to determine the optimal kmer size before using Velvet to produce the assembly [65]. The assembly was further improved using SSPACE [66] to scaffold the contigs of the assembly and GapFiller [67] to close gaps of 1 or more nucleotides.

### Recombination detection, phylogenetic analysis and BAPS clustering

Recombined regions were detected in the alignments of the six disease-associated STs using Gubbins [24]. Phylogenetic trees of these lineages were generated using RAxML v7.0.4 [68], firstly using all SNPs to later allow ancestral sequence reconstruction, and secondly using only the vertically inherited SNPs (i.e. excluding SNPs in recombined regions). A phylogenetic tree of the total 536 isolates was constructed using all the detected SNPs, as the high diversity of the whole collection renders recombination detection very difficult. In all cases, the GTR+GAMMA method for among site rate variation was used and 100 bootstrap replicates were performed to assess support for nodes. The alignment of all 536 genomes against the Paris reference genome was also used to group the isolates into clusters using hierBAPS [47].

### Detection of mobile elements and genomic islands

The annotation files from each of the six reference genomes were parsed to detect regions annotated as “integrase”, “transposase”, “recombinase”, “phage”, “lvrA”, “csrA”, “HTX”, “helix-turn-helix”, “xre”, “conjugal”, “conjugation”, “tra”, “trb”, “vir” and “mobile”. Both the published annotation files of the Paris (ST1) and Alcoy (ST578) complete genomes and those generated using the in-house pipeline at the WTSI were used. However, the new annotations were only considered when the original one was a “hypothetical protein” in order to respect experimentally proven annotations. Plots showing the mapping coverage of each isolate against the corresponding reference genome were also evaluated. Regions over 8kb with no coverage and that did not match repetitive regions were considered as potential mobile regions. Other software to detect mobile genetic elements (MGEs) was also used including AlienHunter [69] and Island Viewer, the latter of which incorporates IslandPick, IslandPath-DIMOB and SIGI-HMM [70]. However, these results were discarded due to major incongruences between them. Finally, manual curation of all predicted MGEs was performed using Artemis v15.0.0 [71] (**S2 Table**).

### Determination of homologous recombination hotspots

In each of the six lineages, any recombined regions predicted by Gubbins that overlap with either repetitive regions or putative MGEs in the reference genome were discarded for the majority of the analysis in this study, leaving only putative homologous recombination regions. An in-house script was used to calculate the number of times each gene and each base had been involved in a homologous recombination event. Recombination “hotspots” were defined as genes with a recombination frequency above the 95^th^ percentile observed in that particular ST and that have been involved in at least two events. Based on these criteria, the minimum number of recombination events that a gene must have been involved in to be considered within a hotspot region was four events in the ST1 lineage and two events in the remaining five STs.

### Analysis of the prominent ST1 hotspot with fastGEAR

FastGEAR [34] was run on 54 individual gene alignments, comprising all 536 strains included in the study, which were extracted from the alignment of all genomes against the Paris reference. These genes span the prominent ST1 hotspot (*lpp1761*-*lpp1794*) and also include 10 flanking loci on either side. The software infers the population structure of individual alignments, allowing detection of lineages in an alignment and “ancestral” and “recent” recombinations between them. The results were compared to those from Gubbins in terms of the number of recombination events predicted in each gene and the sharing of alleles among the different predicted lineages. Notably, if a recombination spans the entire length of an alignment, fastGEAR will detect this as another lineage in the alignment, rather than a recombination. Therefore, to make recombination counts between fastGEAR and Gubbins comparable, we used the estimated phylogeny and post-processed fastGEAR output by identifying branches in the tree where the population structure changed, and interpreted these as recombinations (these can be seen as “blocks” with a colour different from yellow in **S3B Fig** and **S4 Fig**). The scripts used to make this calculation and to produce **S3B Fig** and **S4 Fig** can be found in https://users.ics.aalto.fi/~pemartti/fastGEAR/.

### Inference of recombination donors

Homologous recombination regions were extracted from the ancestral sequences inferred from the nodes of the six phylogenetic trees, constructed prior to recombination removal, using PAML 4 [72]. Specifically, ancestral recombination sequences were extracted from the node downstream of the phylogenetic tree branch on which the recombination event was predicted to have occurred. A custom genome BLAST database (BLAST v2.2.30+) [73] was constructed using *de novo* assemblies and/or complete genomes from all 536 *L*. *pneumophila* isolates used in this study. The reconstructed recombined regions were used as query sequences in BLAST searches against the custom genome database and the NCBI non-redundant nucleotide database. The resulting hits were filtered to remove those against isolates that are descended from the branch in which the recombination event was detected. Of the remaining hits, the BAPS cluster containing the isolate with the highest bit score was considered as the potential donor, provided that the hit covered at least 50% of the recombination fragment length and had a minimum of 99% nucleotide identity. Recombination fragments with no hits that met these thresholds were not assigned a donor cluster (“No donor predicted”). Only recombined regions greater than 500bp were used in this analysis, firstly because they were deemed more likely to be a “true” event, and secondly because small regions would likely have high similarity to many genomes.

## Supporting information

S1 Table *L*. *pneumophila* isolates (*n* = 291) belonging to six major disease-associated lineages. These include 81 ST1 (or ST1-derived), 42 ST23, 72 ST37, 15 ST42, 35 ST62 and 46 ST578 isolates. (1) in the “ST” column indicates ST1-derived isolates. ST: sequence type; Sg: serogroup; clin: clinical; env: environmental; U/k: unknown; TA: travel-associated.
(DOCX)

S2 Table Genomic positions of repetitive regions and predicted mobile genetic elements (MGEs) in the six reference genomes (Paris/ST1; EUL 28/ST23; EUL 165/ST37; EUL 120/ST42; H044120014/ST62; Alcoy/ST578). (DOCX)

S3 Table Genes in recombination hotspots in the six major disease-associated STs. (DOCX)

S4 Table The number of “recent” recombination events predicted by fastGEAR in each of the genes from the prominent ST1 hotspot (*lpp1761*-*1794*), as well as 10 genes upstream and downstream of this region. The number of events that have affected all 536 isolates used in the study are shown, as well as the numbers that have affected isolates belonging to the 6 STs of interest only. An extra column (ST1_blocks) was included with the number of recombinations obtained by post-processing the fastGEAR output by detecting recombinations using the phylogeny (see Methods), corresponding to the coloured blocks different from the background in **S3B Fig**, to make the results comparable with Gubbins. The script used to get these recombination counts can be found in https://users.ics.aalto.fi/~pemartti/fastGEAR/.
(DOCX)

S5 Table Additional *L*. *pneumophila* isolates (*n* = 245) used in the inference of the recombination donors. ST: sequence type; Sg: serogroup; U/k: unknown; TA: travel-associated; NA: not applicable.
(DOCX)

S1 Fig Boxplots showing the number of homologous recombination events detected per vertically inherited SNP (ρ/θ ratio) on each of the branches of the phylogenetic trees belonging to the six major disease-associated STs. (TIF)

S2 Fig Phylogenetic tree of the ST1 lineage with bootstrap values based on 100 replicates. The scale shows the number of SNPs.
(TIF)

S3 Fig Recombination events predicted in the prominent ST1 hotspot as inferred by Gubbins (A) and fastGEAR (B). (A) was extracted from **Fig 2** and shows the zoomed-in illustration of hotspot 6 in the ST1 lineage (*lpp1761*-*lpp1794*). The homologous recombination events are displayed as blocks and coloured according to the BAPS cluster from which they are predicted to be derived. The genes shown at the top of the figure are coloured by the number of times that they have been affected by a homologous recombination event, as predicted by Gubbins (see key at the top right). (B) shows regions of shared ancestry for this hotspot in the ST1 lineage, as predicted by fastGEAR. The genes at the top are coloured by the number of recombinations, corresponding to blocks of segments differing from the yellow background detected in this subset (see key at the top right).
(TIF)

S4 Fig FastGEAR population structure results of the gene-by-gene analysis performed on 54 loci including the most prominent ST1 hotspot (34 genes, *lpp1761-lpp1794*) plus ten flanking genes on each side. The left panel shows the maximum likelihood tree of the core genome alignment of the 536 *L*. *pneumophila* genomes included in the study. The main 6 STs are highlighted in the tree with the background colour representing their BAPS cluster (see **Fig 3**). FastGEAR output is shown per gene, with colours representing the donor lineages of both “recent” and “ancestral” recombination events. Lineage colours were reordered at different genes to optimize visualization as in [34].
(TIF)

S5 Fig The lipopolysaccharide (LPS) locus, comprising hotspot 3 (*lpp0819/neuC* to *lpp0830*), in the ST1 lineage. The recombination events are displayed as blocks, coloured according to the BAPS cluster from which they are predicted to be derived. The genes are shown at the top of the figure and coloured by the number of overlapping recombination regions. A maximum likelihood tree, constructed using only vertically inherited SNPs, is also shown on the left and the scale indicates the number of SNPs.
(TIF)

S6 Fig The percentage nucleotide identity of the recombination fragments to the highest-scoring BLAST hit (A) and the percentage length of the recombination fragment covered by the highest-scoring BLAST hit (B).
(TIF)

S7 Fig The number of recombination events per base detected in the ST1 lineage that are derived from the different BAPS clusters (excluding BAPS cluster 7 from which no events were predicted to be derived). The vertical grey bars correspond to the recombination hotspots.
(TIF)

S8 Fig The percentage nucleotide identity of 8 recombination fragments identified in ST1_28 (BAPS 2) to an isolate, EUL 25 (ST44), from the predicted donor BAPS cluster (BAPS 4), and to a clonally related isolate, ST72_1, from the same ST1 lineage. (TIF)
