# Correction: Dynamics and impact of homologous recombination on the evolution of *Legionella pneumophila*

**DOI:** 10.1371/journal.pgen.1007116

**Published:** 2017-12-01

**Authors:** Sophia David, Leonor Sánchez-Busó, Simon R. Harris, Pekka Marttinen, Christophe Rusniok, Carmen Buchrieser, Timothy G. Harrison, Julian Parkhill

In the Data Availability statement, the accession number ERR35215 is incorrect. This accession number should be ERR352157.
