# Supplementary material for: Dynamics and impact of homologous recombination on the evolution of Legionella pneumophila
Source: PLoS Genet. 2017 Jun 26;13(6):e1006855. doi: 10.1371/journal.pgen.1006855 (PMC5507463; doi:10.1371/journal.pgen.1006855)
Supplement: S1 Table — These include 81 ST1 (or ST1-derived), 42 ST23, 72 ST37, 15 ST42, 35 ST62 and 46 ST578 isolates. (1) in the “ST” column indicates ST1-derived isolates. ST: sequence type; Sg: serogroup; clin: clinical; env: environmental; U/k: unknown; TA: travel-associated. (DOCX) [file pgen.1006855.s001.docx]

**S1 Table**. *L. pneumophila* isolates (*n*=291) belonging to six major disease-associated lineages. These include 81 ST1 (or ST1-derived), 42 ST23, 72 ST37, 15 ST42, 35 ST62 and 46 ST578 isolates. (1) in the “ST” column indicates ST1-derived isolates. ST: sequence type; Sg: serogroup; clin: clinical; env: environmental; U/k: unknown; TA: travel-associated.

| **Isolate name** | **Other name** | **ST** | **Source** | **Country** | **Year** | **Known epidemiologic relatedness** | **Reference** |
| --- | --- | --- | --- | --- | --- | --- | --- |
| Paris | ST1_1 | 1 | clin | France | 2002 | None | Cazalet *et al.* (2004) |
| H034800423 | ST1_2 | 1 | env | UK | 2003 | None | Reuter *et al.* (2013) |
| EUL 55 | ST1_3 | 1 | clin | Spain | 1994 | Related to ST1_15 | David *et al.* (2016) |
| EUL 88 | ST1_4 | 1 | clin | Denmark | 1995 | None | David *et al.* (2016) |
| EUL 93 | ST1_5 | 1 | clin | Denmark | 1992 | Related to ST1_24 and ST1_25 | David *et al.* (2016) |
| EUL 10 | ST1_6 | 1 | env | Switzerland | 1989 | Related to ST1_9 and ST1_20 | David *et al.* (2016) |
| EUL 1 | ST1_7 | 1 | clin | Switzerland | 1998 | None | David *et al.* (2016) |
| EUL 21 | ST1_8 | 1 | env | UK | 1999 | None | David *et al.* (2016) |
| EUL 3 | ST1_9 | 1 | clin | Switzerland | 1989 | Related to ST1_6 and ST1_20 | David *et al.* (2016) |
| EUL 109 | ST1_10 | 1 | env | Sweden | 1992 | None | David *et al.* (2016) |
| EUL 42 | ST1_11 | 1 | clin | Italy | 1999 | None | David *et al.* (2016) |
| EUL 43 | ST1_12 | 1 | clin | Italy | 1999 | None | David *et al.* (2016) |
| EUL 44 | ST1_13 | 1 | env | Italy | 1999 | Related to ST1_28 | David *et al.* (2016) |
| EUL 46 | ST1_14 | 1 | env | Italy | 1999 | None | David *et al.* (2016) |
| EUL 58 | ST1_15 | 1 | env | Spain | 1994 | Related to ST1_3 | David *et al.* (2016) |
| EUL 60 | ST1_16 | 1 | clin | Greece | 1992 | None | David *et al.* (2016) |
| EUL 62 | ST1_17 | 1 | env | Greece | 1989 | None | David *et al.* (2016) |
| EUL 67 | ST1_18 | 1 | clin | Greece | 1995 | None | David *et al.* (2016) |
| EUL 85 | ST1_19 | 1 | clin | Denmark | 1995 | None | David *et al.* (2016) |
| EUL 9 | ST1_20 | 1 | env | Switzerland | 1989 | Related to ST1_6 and ST1_9 | David *et al.* (2016) |
| EUL 82 | ST1_21 | 1 | clin | Denmark | 1994 | None | David *et al.* (2016) |
| EUL 84 | ST1_22 | 1 | clin | Denmark | 1995 | None | David *et al.* (2016) |
| EUL 90 | ST1_23 | 1 | clin | Denmark | U/k | None | David *et al.* (2016) |
| EUL 94 | ST1_24 | 1 | clin | Denmark | 1992 | Related to ST1_5 and ST1_25 | David *et al.* (2016) |
| EUL 95 | ST1_25 | 1 | env | Denmark | 1993 | Related to ST1_5 and ST1_24 | David *et al.* (2016) |
| EUL 104 | ST1_26 | 1 | clin | Sweden | 1992 | None | David *et al.* (2016) |
| EUL 108 | ST1_27 | 1 | clin | Sweden | 1992 | None | David *et al.* (2016) |
| EUL 37 | ST1_28 | 1 | clin | Italy | 1999 | Related to ST1_13 | David *et al.* (2016) |
| EUL 119 | ST1_29 | 1 | clin | Germany | 2005 | None | David *et al.* (2016) |
| EUL 53 | ST1_30 | 1 | clin | Spain | 1995 | None | David *et al.* (2016) |
| OLDA1 (NCTC1208) | ST1_31 | 1 | clin | USA | 1947 | None | David *et al.* (2016) |
| HL 0036 4001 | ST1_32 | 1 | clin | France | 2000 | None | David *et al.* (2016) |
| HL 0230 4015 | ST1_33 | 1 | clin | France | 2002 | None | David *et al.* (2016) |
| HL 0311 1005 | ST1_34 | 1 | env | France | 2003 | None | David *et al.* (2016) |
| HL 0337 3012 | ST1_35 | 1 | env | France | 2003 | None | David *et al.* (2016) |
| HL 0416 3014 | ST1_36 | 1 | clin | France | 2004 | None | David *et al.* (2016) |
| HL 0701 3004 | ST1_37 | 1 | env | France | 2007 | None | David *et al.* (2016) |
| LG 0919 2006 | ST1_38 | 1 | clin | France | 2009 | None | David *et al.* (2016) |
| LG 0940 4015 | ST1_39 | 1 | clin | France | 2009 | None | David *et al.* (2016) |
| LG 1019 1002 | ST1_40 | 1 | clin | France | 2010 | Related to ST1_41 | David *et al.* (2016) |
| LG 1020 3012 | ST1_41 | 1 | env | France | 2010 | Related to ST1_40 | David *et al.* (2016) |
| LG 1101 1012 | ST1_42 | 1 | env | France | 2010 | None | David *et al.* (2016) |
| LG 1105 4025 | ST1_43 | 1 | env | France | 2011 | None | David *et al.* (2016) |
| LG 1118 1044 | ST1_44 | 1 | env | Morocco | 2009 | None | David *et al.* (2016) |
| LG 1139 1124 | ST1_45 | 1 | env | France | 2011 | None | David *et al.* (2016) |
| LP21 | ST1_46 | 1 | clin | Sweden | 1996-1999 | U/k | David *et al.* (2016) |
| LP23 | ST1_47 | 1 | clin | Sweden | 1996-2000 | None | David *et al.* (2016) |
| LT 40/04 | ST1_48 | 1 | clin | Austria | 2004 | None | David *et al.* (2016) |
| NIIB223 | ST1_49 | 1 | env | Japan | 1986 | U/k | David *et al.* (2016) |
| NIIB225 | ST1_50 | 1 | env | Japan | 1986 | U/k | David *et al.* (2016) |
| L 3386/03 | ST1_51 | 1 | env | Austria | 2003 | None | David *et al.* (2016) |
| L 3415/03 | ST1_52 | 1 | env | Austria | 2003 | None | David *et al.* (2016) |
| LG 1014 3009 | ST1_53 | 1 | clin | France | 2010 | None | David *et al.* (2016) |
| NIIB80 | ST1_54 | 1 | clin | Japan | 1981 | None | David *et al.* (2016) |
| LP22 | ST1_55 | 1 | clin | Sweden | 1996-1999 | U/k | David *et al.* (2016) |
| L00-549 | ST1_56 | 1 | clin | Germany | 2000 | None | David *et al.* (2016) |
| E21203 | ST1_57 | 1 | clin | France | 2004 | None | David *et al.* (2016) |
| 2735 | ST1_58 | 1 | env | USA | 2002 | None | David *et al.* (2016) |
| Wien 47-14 | ST1_59 | 1 | env | Austria | 1996 | None | David *et al.* (2016) |
| ID_1688 | ST1_60 | 1 | env | Spain | 2004 | U/k | Sanchez-Buso *et al.* (2014) |
| ID_1690 | ST1_61 | 1 | env | Spain | 2004 | U/k | Sanchez-Buso *et al.* (2014) |
| ID_1828 | ST1_62 | 1 | env | Spain | 2004 | U/k | Sanchez-Buso *et al.* (2014) |
| ID_2041 | ST1_63 | 1 | env | Spain | 2005 | U/k | Sanchez-Buso *et al.* (2014) |
| ID_2947 | ST1_64 | 1 | env | Spain | 2000 | U/k | Sanchez-Buso *et al.* (2014) |
| ID_2948 | ST1_65 | 1 | env | Spain | 2000 | U/k | Sanchez-Buso *et al.* (2014) |
| ID_598 | ST1_66 | 1 | env | Spain | 2002 | U/k | Sanchez-Buso *et al.* (2014) |
| ID_6885 | ST1_67 | 1 | env | Spain | 2011 | U/k | Sanchez-Buso *et al.* (2014) |
| ID_747970 | ST1_68 | 1 | env | Spain | 2009 | U/k | Sanchez-Buso *et al.* (2014) |
| ID_891 | ST1_69 | 1 | env | Spain | 2002 | U/k | Sanchez-Buso *et al.* (2014) |
| H074360702 | ST152_1 | 152 (1) | env | UK | 2007 | None | Underwood *et al.* (2013) |
| EUL 13 | ST5_1 | 5 | clin | UK | 1983 | None | David *et al.* (2016) |
| EUL 14 | ST5_2 | 5 (1) | clin | UK | 1984 | None | David *et al.* (2016) |
| EUL 16 | ST5_3 | 5 (1) | clin | UK | 1984 | None | David *et al.* (2016) |
| EUL 17 | ST7_1 | 7 (1) | clin | UK | 1993 | None | David *et al.* (2016) |
| EUL 113 | ST7_2 | 7 (1) | env | Germany | 1995 | None | David *et al.* (2016) |
| EUL 114 | ST7_3 | 7 (1) | env | Germany | 1995 | None | David *et al.* (2016) |
| EUL 45 | ST72_1 | 72 (1) | clin | Italy | 1999 | None | David *et al.* (2016) |
| EUL 110 | ST10_1 | 10 (1) | clin | Germany | 1993 | None | David *et al.* (2016) |
| EUL 117 | ST6_1 | 6 (1) | clin | Germany | 2005 | None | David *et al.* (2016) |
| EUL 157 | ST8_1 | 8 (1) | env | UK | 2004 | None | David *et al.* (2016) |
| IN-23-G1-C2 (ATCC 35289) | ST390_1 | 390 (1) | env | Netherlands | 1988 | None | David *et al.* (2016) |
| H063280001 | ST23_1 | 23 | clin | UK | 2006 | None | Underwood *et al.* (2013) |
| EUL 8 | ST23_2 | 23 | clin | Switzerland | 1993 | Related to ST23_3 and ST23_4 | David *et al.* (2016) |
| EUL 11 | ST23_3 | 23 | env | Switzerland | 1993 | Related to ST23_2 and ST23_4 | David *et al.* (2016) |
| EUL 12 | ST23_4 | 23 | env | Switzerland | 1993 | Related to ST23_2 and ST23_3 | David *et al.* (2016) |
| EUL 41 | ST23_5 | 23 | clin | Italy | 1999 | None | David *et al.* (2016) |
| EUL 130 | ST23_6 | 23 | clin | Croatia | 1987 | Related to ST23_7 | David *et al.* (2016) |
| EUL 129 | ST23_7 | 23 | clin | Croatia | 1987 | Related to ST23_6 | David *et al.* (2016) |
| EUL 4 | ST23_8 | 23 | clin | Switzerland | 1991 | None | David *et al.* (2016) |
| EUL 28 | ST23_9 | 23 | clin | France | 1994 | None | David *et al.* (2016) |
| HL 0127 3027 | ST23_ 10 | 23 | clin | France | 2001 | None | David *et al.* (2016) |
| HL 0236 5014 | ST23_ 11 | 23 | clin | France | 2002 | None | David *et al.* (2016) |
| HL 0236 5015 | ST23_ 12 | 23 | clin | France | 2002 | None | David *et al.* (2016) |
| HL 0307 1012 | ST23_ 13 | 23 | clin | France | 2003 | None | David *et al.* (2016) |
| HL 0339 3028 | ST23_ 14 | 23 | clin | France | 2003 | None | David *et al.* (2016) |
| HL 0437 1017 | ST23_ 15 | 23 | clin | France | 2004 | None | David *et al.* (2016) |
| HL 0443 3031 | ST23_ 16 | 23 | clin | France | 2004 | None | David *et al.* (2016) |
| HL 0506 3005 | ST23_ 17 | 23 | clin | France | 2005 | None | David *et al.* (2016) |
| HL 0532 2037 | ST23_ 18 | 23 | env | France | 2005 | None | David *et al.* (2016) |
| HL 0541 5018 | ST23_ 19 | 23 | clin | France | 2005 | None | David *et al.* (2016) |
| HL 0604 3045 | ST23_ 20 | 23 | clin | France | 2006 | None | David *et al.* (2016) |
| HL 0637 3021 | ST23_ 21 | 23 | clin | France | 2006 | None | David *et al.* (2016) |
| HL 0709 3017 | ST23_ 22 | 23 | clin | France | 2007 | None | David *et al.* (2016) |
| LG 0751 2008 | ST23_ 23 | 23 | clin | France | 2007 | None | David *et al.* (2016) |
| LG 0834 5006 | ST23_ 24 | 23 | clin | France | 2008 | None | David *et al.* (2016) |
| LG 0839 2025 | ST23_ 25 | 23 | clin | France | 2008 | None | David *et al.* (2016) |
| LG 0915 3012 | ST23_ 26 | 23 | env | France | 2009 | None | David *et al.* (2016) |
| LG 0935 3013 | ST23_ 27 | 23 | env | France | 2009 | None | David *et al.* (2016) |
| LG 0940 3015 | ST23_ 28 | 23 | clin | France | 2009 | None | David *et al.* (2016) |
| LG 0945 4021 | ST23_ 29 | 23 | clin | France | 2009 | None | David *et al.* (2016) |
| LG 1025 5002 | ST23_ 30 | 23 | clin | France | 2010 | None | David *et al.* (2016) |
| LG 1036 3013 | ST23_ 31 | 23 | env | France | 2010 | None | David *et al.* (2016) |
| LG 1048 1020 | ST23_ 32 | 23 | clin | France | 2010 | None | David *et al.* (2016) |
| LG 1127 2006 | ST23_ 33 | 23 | clin | France | 2011 | None | David *et al.* (2016) |
| LG 1136 3009 | ST23_ 34 | 23 | clin | France | 2011 | None | David *et al.* (2016) |
| LG 1140 2026 | ST23_ 35 | 23 | clin | France | 2011 | None | David *et al.* (2016) |
| LG 1224 2012 | ST23_ 36 | 23 | clin | France | 2012 | None | David *et al.* (2016) |
| LG 1246 5006 | ST23_ 37 | 23 | clin | France | 2012 | None | David *et al.* (2016) |
| ID_125_BC1 | ST23_ 38 | 23 | clin | Spain | 2012 | U/k | Sanchez-Buso *et al.* (2015) |
| ID_192091_BC52 | ST23_ 39 | 23 | clin | Spain | 2012 | U/k | Sanchez-Buso *et al.* (2015) |
| ID_4029_BC37 | ST23_ 40 | 23 | env | Spain | 2012 | U/k | Sanchez-Buso *et al.* (2015) |
| ID_50291_BC50 | ST23_ 41 | 23 | clin | Spain | 2012 | U/k | Sanchez-Buso *et al.* (2015) |
| ID_50726_BC51 | ST23_ 42 | 23 | clin | Spain | 2012 | U/k | Sanchez-Buso *et al.* (2015) |
| EUL 165 | ST37_1 | 37 | clin | UK | 2003 | None | Underwood *et al.* (2013) |
| H064240448 | ST37_2 | 37 | env | UK | 2006 | None | David *et al.* (2016) |
| LC0731 | ST37_3 | 37 | clin | UK | 1989 | Related to ST37_4, ST37_5, ST37_59, ST37_61 and ST37_63 | David *et al.* (2016) |
| LC0732 | ST37_4 | 37 | clin | UK | 1989 | Related to ST37_3, ST37_5, ST37_59, ST37_61 and ST37_63 | David *et al.* (2016) |
| LC0763 | ST37_5 | 37 | env | UK | 1989 | Related to ST37_3, ST37_4, ST37_59, ST37_61 and ST37_63 | David *et al.* (2016) |
| LC5694 | ST37_6 | 37 | clin | UK | 2000 | None | David *et al.* (2016) |
| LC5722 | ST37_7 | 37 | clin | UK | 2000 | None | David *et al.* (2016) |
| LC5738 | ST37_8 | 37 | clin | UK | 2000 | None | David *et al.* (2016) |
| LC5755 | ST37_9 | 37 | clin | UK | 2000 | None | David *et al.* (2016) |
| LC5908 | ST37_ 10 | 37 | clin | UK | 2001 | None | David *et al.* (2016) |
| LC6163 | ST37_ 11 | 37 | clin | UK | 2002 | None | David *et al.* (2016) |
| LC6267 | ST37_ 12 | 37 | clin | UK | 2002 | None | David *et al.* (2016) |
| LC6268 | ST37_ 13 | 37 | clin | UK | 2002 | None | David *et al.* (2016) |
| LC6228 | ST37_ 14 | 37 | clin | UK | 2002 | None | David *et al.* (2016) |
| H041380048 | ST37_ 15 | 37 | clin | UK | 2004 | Related to ST37_23 | David *et al.* (2016) |
| H042960010 | ST37_ 16 | 37 | clin | UK | 2004 | None | David *et al.* (2016) |
| H061140013 | ST37_ 17 | 37 | clin | UK | 2006 | None | David *et al.* (2016) |
| H071880001 | ST37_ 18 | 37 | clin | UK | 2007 | None | David *et al.* (2016) |
| H073060003 | ST37_ 19 | 37 | clin | UK | 2007 | None | David *et al.* (2016) |
| H080820009 | ST37_ 20 | 37 | clin | UK | 2008 | None | David *et al.* (2016) |
| LC6058 | ST37_ 21 | 37 | clin | U/k (TA) | 2001 | None | David *et al.* (2016) |
| LC6293 | ST37_ 22 | 37 | clin | U/k (TA) | 2002 | None | David *et al.* (2016) |
| H041640791 | ST37_ 23 | 37 | env | UK | 2004 | Related to ST37_15 | David *et al.* (2016) |
| LC6788 | ST37_ 24 | 37 | clin | U/k (TA) | 2003 | None | David *et al.* (2016) |
| H062660463 | ST37_ 25 | 37 | clin | U/k (TA) | 2006 | None | David *et al.* (2016) |
| H073900557 | ST37_ 26 | 37 | clin | U/k (TA) | 2007 | None | David *et al.* (2016) |
| LC1127 | ST37_ 27 | 37 | clin | UK | 1989 | None | David *et al.* (2016) |
| H084760449 | ST37_ 28 | 37 | clin | UK | 2008 | None | David *et al.* (2016) |
| H085020185 | ST37_ 29 | 37 | clin | UK | 2008 | None | David *et al.* (2016) |
| H090320386 | ST37_ 30 | 37 | clin | UK | 2009 | None | David *et al.* (2016) |
| H044260061 | ST37_ 31 | 37 | env | UK | 2004 | None | David *et al.* (2016) |
| H093140322 | ST37_ 32 | 37 | clin | UK | 2009 | Related to ST37_33 | David *et al.* (2016) |
| H093160422 | ST37_ 33 | 37 | env | UK | 2009 | Related to ST37_32 | David *et al.* (2016) |
| H092760433 | ST37_ 34 | 37 | clin | U/k (TA) | 2009 | None | David *et al.* (2016) |
| H100940111 | ST37_ 35 | 37 | clin | UK | 2010 | None | David *et al.* (2016) |
| H101760092 | ST37_ 36 | 37 | clin | UK | 2010 | None | David *et al.* (2016) |
| H101820190 | ST37_ 37 | 37 | clin | UK | 2010 | None | David *et al.* (2016) |
| H102020414 | ST37_ 38 | 37 | clin | UK | 2010 | None | David *et al.* (2016) |
| H101980130 | ST37_ 39 | 37 | clin | U/k (TA) | 2010 | None | David *et al.* (2016) |
| H103820081 | ST37_ 40 | 37 | clin | UK | 2010 | None | David *et al.* (2016) |
| H120240685 | ST37_ 41 | 37 | clin | Slovenia | 2010 | None | David *et al.* (2016) |
| H104320293 | ST37_ 42 | 37 | env | UK | 2010 | None | David *et al.* (2016) |
| H113180118 | ST37_ 43 | 37 | clin | UK | 2011 | Related to ST37_44 | David *et al.* (2016) |
| H113340664 | ST37_ 44 | 37 | env | UK | 2011 | Related to ST37_43 | David *et al.* (2016) |
| H113280076 | ST37_ 45 | 37 | clin | UK | 2011 | None | David *et al.* (2016) |
| H113660550 | ST37_ 46 | 37 | clin | UK | 2011 | None | David *et al.* (2016) |
| H114740454 | ST37_ 47 | 37 | clin | UK | 2011 | None | David *et al.* (2016) |
| H115040456 | ST37_ 48 | 37 | clin | UK | 2011 | None | David *et al.* (2016) |
| H111580389 | ST37_ 49 | 37 | clin | UK | 2011 | None | David *et al.* (2016) |
| H113780240 | ST37_ 50 | 37 | clin | U/k (TA) | 2011 | None | David *et al.* (2016) |
| H083920177 | ST37_ 51 | 37 | clin | UK | 2008 | Related to ST37_52 | David *et al.* (2016) |
| H084140691 | ST37_ 52 | 37 | env | UK | 2008 | Related to ST37_51 | David *et al.* (2016) |
| H081180019 | ST37_ 53 | 37 | env | UK | 2008 | None | David *et al.* (2016) |
| H103260667 | ST37_ 54 | 37 | env | Greece | 2010 | None | David *et al.* (2016) |
| LC464 | ST37_ 55 | 37 | clin | UK | 1987 | None | David *et al.* (2016) |
| LC0512 | ST37_ 56 | 37 | clin | U/k (TA) | 1988 | None | David *et al.* (2016) |
| LC0565 | ST37_ 57 | 37 | clin | UK | 1988 | Related to ST37_58, ST37_69, ST37_ST37_70 and ST37_71 | David *et al.* (2016) |
| LC0583 | ST37_ 58 | 37 | clin | UK | 1988 | Related to ST37_57, ST37_69, ST37_ST37_70 and ST37_71 | David *et al.* (2016) |
| LC0782 | ST37_ 59 | 37 | clin | UK | 1989 | Related to ST37_3, ST37_4, ST37_5, ST37_61 and ST37_63 | David *et al.* (2016) |
| LC0794 | ST37_ 60 | 37 | clin | UK | 1989 | Related to ST37_62 | David *et al.* (2016) |
| LC0795 | ST37_ 61 | 37 | clin | UK | 1989 | Related to ST37_3, ST37_4, ST37_5, ST37_59 and ST37_63 | David *et al.* (2016) |
| LC0798 | ST37_ 62 | 37 | clin | UK | 1989 | Related to ST37_60 | David *et al.* (2016) |
| LC0801 | ST37_ 63 | 37 | clin | UK | 1989 | Related to ST37_3, ST37_4, ST37_5, ST37_59 and ST37_61 | David *et al.* (2016) |
| EUL 166  /LP056 | ST37_ 64 | 37 | env | UK | 2003 | Related to ST37_1 | David *et al.* (2016) |
| EUL 69 | ST37_ 65 | 37 | clin | UK | 1995 | None | David *et al.* (2016) |
| EUL 73 | ST37_ 66 | 37 | clin | UK | 1996 | Related to ST37_67 and ST37_68 | David *et al.* (2016) |
| EUL 78 | ST37_ 67 | 37 | clin | UK | 1996 | Related to ST37_66 and ST37_68 | David *et al.* (2016) |
| EUL 79 | ST37_ 68 | 37 | clin | UK | 1996 | Related to ST37_66 and ST37_67 | David *et al.* (2016) |
| EUL 132 | ST37_ 69 | 37 | clin | UK | 1988 | Related to ST37_57, ST37_58, ST37_70 and ST37_71 | David *et al.* (2016) |
| EUL 133 | ST37_ 70 | 37 | clin | UK | 1988 | Related to ST37_57, ST37_58, ST37_69 and ST37_71 | David *et al.* (2016) |
| EUL 134 | ST37_ 71 | 37 | clin | UK | 1988 | Related to ST37_57, ST37_58, ST37_69 and ST37_70 | David *et al.* (2016) |
| EUL 131 | ST37_ 72 | 37 | clin | UK | 1988 | None | David *et al.* (2016) |
| EUL 6 | ST42_1 | 42 | clin | Switzerland | 1999 | None | David *et al.* (2016) |
| EUL 27 | ST42_2 | 42 | clin | France | 2005 | None | David *et al.* 2016 |
| EUL 39 | ST42_3 | 42 | clin | Italy | 1999 | None | David *et al.* 2016 |
| EUL 50 | ST42_4 | 42 | clin | Spain | 1996 | None | David *et al.* 2016 |
| EUL 75 | ST42_5 | 42 | clin | UK | 1995 | None | David *et al.* 2016 |
| EUL 105 | ST42_6 | 42 | clin | Sweden | 1991 | None | David *et al.* 2016 |
| EUL 116 | ST42_7 | 42 | clin | Germany | 1996 | None | David *et al.* 2016 |
| EUL 120 | ST42_8 | 42 | clin | Germany | 1999 | Related to EUL 121 | David *et al.* 2016 |
| EUL 121 | ST42_9 | 42 | clin | Germany | 1999 | Related to EUL 120 | David *et al.* 2016 |
| EUL 122 | ST42_ 10 | 42 | clin | Unknown | 1987 | Related to EUL 123 | David *et al.* 2016 |
| EUL 123 | ST42_ 11 | 42 | clin | Unknown | 1987 | Related to EUL 122 | David *et al.* 2016 |
| EUL 124 | ST42_ 12 | 42 | clin | UK | 1987 | Related to EUL 125 | David *et al.* 2016 |
| EUL 125 | ST42_ 13 | 42 | clin | UK | 1987 | Related to EUL 124 | David *et al.* 2016 |
| Wadsworth/130b | ST42_ 14 | 42 | clin | USA | U/k | None | Schroeder *et al.* (2010) |
| H044540088 | ST42_ 15 | 42 | clin | UK | 2004 | None | Underwood *et al.* (2013) |
| H064180002 | ST62_1 | 62 | clin | UK | 2006 | Related to ST62_19 | Underwood *et al.* (2013) |
| H043540106 | ST62_2 | 62 | clin | U/k (TA) | 2004 | None | David *et al.* (2016) |
| H044120014 | ST62_3 | 62 | clin | Bulgaria | 2004 | None | David *et al.* (2016) |
| H052780022 | ST62_4 | 62 | clin | UK | 2005 | None | David *et al.* (2016) |
| H054280040 | ST62_5 | 62 | clin | UK | 2005 | None | David *et al.* (2016) |
| H063680003 | ST62_6 | 62 | clin | UK | 2006 | None | David *et al.* (2016) |
| H063840008 | ST62_7 | 62 | clin | UK | 2006 | None | David *et al.* (2016) |
| H073660582 | ST62_8 | 62 | clin | UK | 2007 | None | David *et al.* (2016) |
| LC5804 | ST62_9 | 62 | clin | UK | 2000 | None | David *et al.* (2016) |
| H063760005 | ST62_ 10 | 62 | clin | UK | 2006 | None | David *et al.* (2016) |
| H064240003 | ST62_ 11 | 62 | clin | UK | 2006 | None | David *et al.* (2016) |
| H065040012 | ST62_ 12 | 62 | clin | UK | 2007 | None | David *et al.* (2016) |
| H070140635 | ST62_ 13 | 62 | clin | UK | 2007 | None | David *et al.* (2016) |
| H073020039 | ST62_ 14 | 62 | clin | UK | 2007 | None | David *et al.* (2016) |
| H073320399 | ST62_ 15 | 62 | clin | UK | 2007 | None | David *et al.* (2016) |
| H073440003 | ST62_ 16 | 62 | clin | UK | 2007 | None | David *et al.* (2016) |
| LC6009 | ST62_ 17 | 62 | clin | U/k (TA) | 2001 | None | David *et al.* (2016) |
| H083140015 | ST62_ 18 | 62 | clin | UK | 2008 | None | David *et al.* (2016) |
| H064180019 | ST62_ 19 | 62 | env | UK | 2006 | Related to ST62_1 | David *et al.* (2016) |
| H093400182 | ST62_ 20 | 62 | clin | UK | 2009 | None | David *et al.* (2016) |
| H094760070 | ST62_ 21 | 62 | clin | UK | 2009 | None | David *et al.* (2016) |
| H094800237 | ST62_ 22 | 62 | clin | UK | 2009 | None | David *et al.* (2016) |
| H110480715 | ST62_ 23 | 62 | clin | UK | 2011 | None | David *et al.* (2016) |
| H112840293 | ST62_ 24 | 62 | clin | UK | 2011 | None | David *et al.* (2016) |
| H114100406 | ST62_ 25 | 62 | clin | Greece | 2011 | None | David *et al.* (2016) |
| H120240362 | ST62_ 26 | 62 | clin | UK | 2012 | None | David *et al.* (2016) |
| H104640262 | ST62_ 27 | 62 | clin | U/k (TA) | 2010 | None | David *et al.* (2016) |
| H123140428 | ST62_ 28 | 62 | env | UK | 2012 | None | David *et al.* (2016) |
| H123460520 | ST62_ 29 | 62 | clin | UK | 2012 | None | David *et al.* (2016) |
| H124360642 | ST62_ 30 | 62 | clin | UK | 2012 | None | David *et al.* (2016) |
| EUL 54 | ST62_ 31 | 62 | clin | Spain | 1994 | Related to ST62_32 | David *et al.* (2016) |
| EUL 57 | ST62_ 32 | 62 | env | Spain | 1995 | Related to ST62_31 | David *et al.* (2016) |
| EUL 71 | ST62_ 33 | 62 | clin | UK | 1996 | Related to ST62_34 and ST62_35 | David *et al.* (2016) |
| EUL 76 | ST62_ 34 | 62 | clin | UK | 1996 | Related to ST62_33 and ST62_35 | David *et al.* (2016) |
| EUL 77 | ST62_ 35 | 62 | clin | UK | 1996 | Related to ST62_33 and ST62_34 | David *et al.* (2016) |
| ID_2680_BC17 | ST578_1 | 578 | clin | Spain | 2000 | U/k | Sanchez-Buso *et al.* (2014) |
| ID_2301_BC14 | ST578_2 | 578 | clin | Spain | 1999 | U/k | Sanchez-Buso *et al.* (2014) |
| ID_2376_BC15 | ST578_3 | 578 | clin | Spain | 1999 | U/k | Sanchez-Buso *et al.* (2014) |
| ID_3009_BC21 | ST578_4 | 578 | clin | Spain | 2000 | U/k | Sanchez-Buso *et al.* (2014) |
| ID_3108_BC23 | ST578_5 | 578 | clin | Spain | 2000 | U/k | Sanchez-Buso *et al.* (2014) |
| ID_3109_BC24 | ST578_6 | 578 | clin | Spain | 2000 | U/k | Sanchez-Buso *et al.* (2014) |
| ID_3110_BC25 | ST578_7 | 578 | clin | Spain | 2000 | U/k | Sanchez-Buso *et al.* (2014) |
| ID_3355_BC32 | ST578_8 | 578 | clin | Spain | 2000 | U/k | Sanchez-Buso *et al.* (2014) |
| ID_3785_BC34 | ST578_9 | 578 | clin | Spain | 2001 | U/k | Sanchez-Buso *et al.* (2014) |
| ID_3908_BC36 | ST578_10 | 578 | clin | Spain | 2001 | U/k | Sanchez-Buso *et al.* (2014) |
| ID_5856_BC39 | ST578_11 | 578 | clin | Spain | 2002 | U/k | Sanchez-Buso *et al.* (2014) |
| ID_6536_BC40 | ST578_12 | 578 | clin | Spain | 2002 | U/k | Sanchez-Buso *et al.* (2014) |
| ID_7147_BC42 | ST578_13 | 578 | clin | Spain | 2003 | U/k | Sanchez-Buso *et al.* (2014) |
| ID_8141_BC45 | ST578_14 | 578 | clin | Spain | 2003 | U/k | Sanchez-Buso *et al.* (2014) |
| ID_8189_BC46 | ST578_15 | 578 | clin | Spain | 2003 | U/k | Sanchez-Buso *et al.* (2014) |
| ID_8190_BC47 | ST578_16 | 578 | clin | Spain | 2003 | U/k | Sanchez-Buso *et al.* (2014) |
| ID_8227_BC48 | ST578_17 | 578 | clin | Spain | 2003 | U/k | Sanchez-Buso *et al.* (2014) |
| ID_8228_BC49 | ST578_18 | 578 | clin | Spain | 2003 | U/k | Sanchez-Buso *et al.* (2014) |
| ID_480203_BC53 | ST578_19 | 578 | clin | Spain | 2009 | U/k | Sanchez-Buso *et al.* (2014) |
| ID_480295_BC55 | ST578_20 | 578 | clin | Spain | 2009 | U/k | Sanchez-Buso *et al.* (2014) |
| ID_480372_BC56 | ST578_21 | 578 | clin | Spain | 2009 | U/k | Sanchez-Buso *et al.* (2014) |
| ID_480392_BC57 | ST578_22 | 578 | clin | Spain | 2009 | U/k | Sanchez-Buso *et al.* (2014) |
| ID_747968_BC72 | ST578_23 | 578 | env | Spain | 2009 | U/k | Sanchez-Buso *et al.* (2014) |
| ID_747969_BC73 | ST578_24 | 578 | env | Spain | 2009 | U/k | Sanchez-Buso *et al.* (2014) |
| ID_747973_BC75 | ST578_25 | 578 | env | Spain | 2009 | U/k | Sanchez-Buso *et al.* (2014) |
| ID_481107_BC58 | ST578_26 | 578 | clin | Spain | 2009 | U/k | Sanchez-Buso *et al.* (2014) |
| ID_481441_BC59 | ST578_27 | 578 | clin | Spain | 2009 | U/k | Sanchez-Buso *et al.* (2014) |
| ID_481707_BC60 | ST578_28 | 578 | clin | Spain | 2009 | U/k | Sanchez-Buso *et al.* (2014) |
| ID_481710_BC61 | ST578_29 | 578 | clin | Spain | 2009 | U/k | Sanchez-Buso *et al.* (2014) |
| ID_1190176_BC76 | ST578_30 | 578 | env | Spain | 2010 | U/k | Sanchez-Buso *et al.* (2014) |
| ID_489571_BC65 | ST578_31 | 578 | clin | Spain | 2010 | U/k | Sanchez-Buso *et al.* (2014) |
| ID_489956_BC66 | ST578_32 | 578 | clin | Spain | 2010 | U/k | Sanchez-Buso *et al.* (2014) |
| ID_490679_BC68 | ST578_33 | 578 | clin | Spain | 2010 | U/k | Sanchez-Buso *et al.* (2014) |
| ID_490738_BC69 | ST578_34 | 578 | clin | Spain | 2010 | U/k | Sanchez-Buso *et al.* (2014) |
| ID_1925_BC12 | ST578_35 | 578 | env | Spain | 2004 | U/k | Sanchez-Buso *et al.* (2014) |
| ID_3499_BC33 | ST578_36 | 578 | clin | Spain | 2001 | U/k | Sanchez-Buso *et al.* (2014) |
| ID_3786_BC35 | ST578_37 | 578 | clin | Spain | 2001 | U/k | Sanchez-Buso *et al.* (2014) |
| ID_480263_BC54 | ST578_38 | 578 | clin | Spain | 2009 | U/k | Sanchez-Buso *et al.* (2014) |
| ID_481898_BC62 | ST578_39 | 578 | clin | Spain | 2009 | U/k | Sanchez-Buso *et al.* (2014) |
| ID_481944_BC63 | ST578_40 | 578 | clin | Spain | 2009 | U/k | Sanchez-Buso *et al.* (2014) |
| ID_489154_BC64 | ST578_41 | 578 | clin | Spain | 2010 | U/k | Sanchez-Buso *et al.* (2014) |
| ID_490456_BC67 | ST578_42 | 578 | clin | Spain | 2010 | U/k | Sanchez-Buso *et al.* (2014) |
| ID_5228_BC38 | ST578_43 | 578 | clin | Spain | 2002 | U/k | Sanchez-Buso *et al.* (2014) |
| ID_7371_BC43 | ST578_44 | 578 | clin | Spain | 2003 | U/k | Sanchez-Buso *et al.* (2014) |
| ID_8004_BC44 | ST578_45 | 578 | clin | Spain | 2003 | U/k | Sanchez-Buso *et al.* (2014) |
| Alcoy | ST578_46 | 578 | clin | Spain | 1999 | U/k | D’Auria *et al.* (2010) |
