# Supplementary material for: Dynamics and impact of homologous recombination on the evolution of Legionella pneumophila
Source: PLoS Genet. 2017 Jun 26;13(6):e1006855. doi: 10.1371/journal.pgen.1006855 (PMC5507463; doi:10.1371/journal.pgen.1006855)
Supplement: S2 Table — (DOCX) [file pgen.1006855.s002.docx]

**S2 Table**. Genomic positions of repetitive regions and predicted mobile genetic elements (MGEs) in the six reference genomes (Paris/ST1; EUL 28/ST23; EUL 165/ST37; EUL 120/ST42; H044120014/ST62; Alcoy/ST578).

| **Reference genome** | **MGEs/repetitive regions** | **Start (bp)** | **End (bp)** |
| --- | --- | --- | --- |
| Paris (ST1) | MGEs | 46159 | 47563 |
|  |  | 66766 | 85124 |
|  |  | 135178 | 136374 |
|  |  | 183008 | 234034 |
|  |  | 237675 | 238718 |
|  |  | 324918 | 332469 |
|  |  | 376713 | 377346 |
|  |  | 793632 | 795036 |
|  |  | 862645 | 871886 |
|  |  | 954348 | 954884 |
|  |  | 961746 | 964139 |
|  |  | 991147 | 992551 |
|  |  | 1160427 | 1219613 |
|  |  | 1733202 | 1746135 |
|  |  | 1757883 | 1760206 |
|  |  | 2046691 | 2048095 |
|  |  | 2096462 | 2097866 |
|  |  | 2112357 | 2114389 |
|  |  | 2154197 | 2159177 |
|  |  | 2205207 | 2206611 |
|  |  | 2302584 | 2306608 |
|  |  | 2311680 | 2312429 |
|  |  | 2315629 | 2318863 |
|  |  | 2322799 | 2323173 |
|  |  | 2408503 | 2419758 |
|  |  | 2581017 | 2582223 |
|  |  | 2654264 | 2776774 |
|  |  | 3280794 | 3299038 |
|  |  | 3307993 | 3309397 |
|  |  | 3370307 | 3374926 |
|  | Repetitive regions | 46017 | 47645 |
|  |  | 84055 | 84184 |
|  |  | 84186 | 84417 |
|  |  | 84432 | 84549 |
|  |  | 84551 | 84784 |
|  |  | 84786 | 84925 |
|  |  | 135135 | 135551 |
|  |  | 135553 | 136387 |
|  |  | 136389 | 136532 |
|  |  | 192590 | 192762 |
|  |  | 225557 | 225727 |
|  |  | 225729 | 225960 |
|  |  | 225977 | 226094 |
|  |  | 226189 | 226420 |
|  |  | 226580 | 226757 |
|  |  | 226759 | 227116 |
|  |  | 227118 | 228316 |
|  |  | 228318 | 228573 |
|  |  | 229902 | 230631 |
|  |  | 230659 | 230888 |
|  |  | 233038 | 233618 |
|  |  | 233620 | 233790 |
|  |  | 233858 | 234049 |
|  |  | 237451 | 238747 |
|  |  | 376427 | 376528 |
|  |  | 376530 | 376754 |
|  |  | 376931 | 377236 |
|  |  | 377238 | 377379 |
|  |  | 432728 | 437887 |
|  |  | 438476 | 439233 |
|  |  | 439235 | 439668 |
|  |  | 454759 | 455516 |
|  |  | 455518 | 455951 |
|  |  | 672085 | 672248 |
|  |  | 675276 | 678229 |
|  |  | 678231 | 678473 |
|  |  | 678475 | 680279 |
|  |  | 680305 | 680448 |
|  |  | 753773 | 776812 |
|  |  | 793490 | 795118 |
|  |  | 866982 | 867892 |
|  |  | 867894 | 869122 |
|  |  | 908097 | 908302 |
|  |  | 908696 | 909294 |
|  |  | 941258 | 941606 |
|  |  | 941708 | 941966 |
|  |  | 944115 | 944463 |
|  |  | 944565 | 944823 |
|  |  | 991005 | 992637 |
|  |  | 1160691 | 1162835 |
|  |  | 1214103 | 1214280 |
|  |  | 1214282 | 1214639 |
|  |  | 1214641 | 1215839 |
|  |  | 1215841 | 1216096 |
|  |  | 1217551 | 1217819 |
|  |  | 1400722 | 1400944 |
|  |  | 1612592 | 1612744 |
|  |  | 1612856 | 1613008 |
|  |  | 1741749 | 1745218 |
|  |  | 1758907 | 1759195 |
|  |  | 1759197 | 1759527 |
|  |  | 1759529 | 1759705 |
|  |  | 1759745 | 1759864 |
|  |  | 1759896 | 1760229 |
|  |  | 1798998 | 1799103 |
|  |  | 2046607 | 2049869 |
|  |  | 2096347 | 2098442 |
|  |  | 2205123 | 2208381 |
|  |  | 2302555 | 2304718 |
|  |  | 2311797 | 2311936 |
|  |  | 2312032 | 2312268 |
|  |  | 2312397 | 2312657 |
|  |  | 2317698 | 2319146 |
|  |  | 2373166 | 2373308 |
|  |  | 2375190 | 2375335 |
|  |  | 2375455 | 2375567 |
|  |  | 2382059 | 2382201 |
|  |  | 2384080 | 2384225 |
|  |  | 2384345 | 2384457 |
|  |  | 2400599 | 2400704 |
|  |  | 2565424 | 2565773 |
|  |  | 2580974 | 2581390 |
|  |  | 2581392 | 2582226 |
|  |  | 2582228 | 2582371 |
|  |  | 2654235 | 2656381 |
|  |  | 2664412 | 2664542 |
|  |  | 2746607 | 2748753 |
|  |  | 2754267 | 2755340 |
|  |  | 2755342 | 2756858 |
|  |  | 3020965 | 3021084 |
|  |  | 3184953 | 3185096 |
|  |  | 3186783 | 3191125 |
|  |  | 3198943 | 3199106 |
|  |  | 3290982 | 3292977 |
|  |  | 3307851 | 3310581 |
|  |  | 3315696 | 3315883 |
|  |  | 3370184 | 3370472 |
|  |  | 3370474 | 3370804 |
|  |  | 3370806 | 3370982 |
|  |  | 3371018 | 3371137 |
|  |  | 3371168 | 3371501 |
|  |  | 3453430 | 3453635 |
|  |  | 3453637 | 3454166 |
|  |  | 3454465 | 3454670 |
|  |  | 3454672 | 3455201 |
| EUL 28 (ST23) | MGEs | 68459 | 87081 |
|  |  | 184525 | 201357 |
|  |  | 540430 | 541401 |
|  |  | 889104 | 889640 |
|  |  | 1090127 | 1209082 |
|  |  | 1262908 | 1264312 |
|  |  | 1293776 | 1296768 |
|  |  | 1779836 | 1781240 |
|  |  | 2265319 | 2390833 |
|  |  | 2628381 | 2629577 |
|  |  | 2701634 | 2766320 |
|  |  | 2990966 | 2999528 |
|  |  | 3374539 | 3378950 |
|  |  | 3388959 | 3390363 |
|  | Repetitive regions | 43294 | 43620 |
|  |  | 43680 | 44257 |
|  |  | 44259 | 44373 |
|  |  | 280639 | 280870 |
|  |  | 280920 | 281161 |
|  |  | 326366 | 327641 |
|  |  | 343575 | 344233 |
|  |  | 344239 | 344583 |
|  |  | 381793 | 383676 |
|  |  | 383726 | 386952 |
|  |  | 387541 | 387740 |
|  |  | 387841 | 388241 |
|  |  | 388243 | 388637 |
|  |  | 403824 | 404023 |
|  |  | 404124 | 404524 |
|  |  | 404526 | 404920 |
|  |  | 540176 | 540312 |
|  |  | 540446 | 540581 |
|  |  | 540583 | 540762 |
|  |  | 540764 | 541546 |
|  |  | 622714 | 622879 |
|  |  | 625907 | 626170 |
|  |  | 626172 | 627790 |
|  |  | 627805 | 631091 |
|  |  | 704322 | 721519 |
|  |  | 846937 | 847044 |
|  |  | 847251 | 847358 |
|  |  | 897056 | 897212 |
|  |  | 948671 | 948995 |
|  |  | 1262765 | 1264598 |
|  |  | 1397268 | 1397598 |
|  |  | 1604321 | 1604454 |
|  |  | 1730876 | 1730989 |
|  |  | 1779800 | 1781421 |
|  |  | 1822149 | 1823203 |
|  |  | 2065597 | 2066568 |
|  |  | 2085857 | 2087011 |
|  |  | 2119394 | 2119507 |
|  |  | 2165562 | 2166726 |
|  |  | 2222017 | 2223151 |
|  |  | 2382957 | 2384245 |
|  |  | 2403947 | 2404150 |
|  |  | 2427445 | 2428388 |
|  |  | 2434402 | 2434604 |
|  |  | 2435853 | 2436257 |
|  |  | 2436737 | 2436898 |
|  |  | 2437165 | 2437295 |
|  |  | 2443299 | 2443501 |
|  |  | 2444750 | 2445154 |
|  |  | 2445631 | 2445792 |
|  |  | 2446059 | 2446189 |
|  |  | 2449041 | 2449151 |
|  |  | 2451145 | 2451257 |
|  |  | 2451978 | 2452086 |
|  |  | 2452088 | 2452421 |
|  |  | 2452430 | 2453097 |
|  |  | 2453206 | 2453549 |
|  |  | 2453847 | 2453957 |
|  |  | 2455957 | 2456069 |
|  |  | 2456790 | 2456898 |
|  |  | 2456900 | 2457233 |
|  |  | 2457242 | 2457909 |
|  |  | 2458018 | 2458361 |
|  |  | 2466814 | 2466913 |
|  |  | 2508190 | 2508297 |
|  |  | 2629018 | 2629800 |
|  |  | 2695209 | 2695485 |
|  |  | 2701604 | 2702892 |
|  |  | 2757054 | 2758339 |
|  |  | 2800215 | 2800322 |
|  |  | 2827913 | 2828229 |
|  |  | 2971986 | 2972085 |
|  |  | 3015972 | 3017357 |
|  |  | 3029597 | 3029929 |
|  |  | 3196343 | 3196590 |
|  |  | 3197873 | 3200048 |
|  |  | 3200972 | 3202414 |
|  |  | 3210094 | 3210419 |
|  |  | 3388818 | 3390719 |
|  |  | 3459212 | 3459417 |
|  |  | 3459435 | 3459762 |
|  |  | 3459764 | 3460020 |
|  |  | 3460247 | 3460452 |
|  |  | 3460470 | 3460797 |
|  |  | 3460799 | 3461055 |
| EUL 165 (ST37) | MGEs | 172790 | 183163 |
|  |  | 527549 | 528334 |
|  |  | 740653 | 742059 |
|  |  | 867654 | 868190 |
|  |  | 904453 | 905857 |
|  |  | 1073685 | 1221017 |
|  |  | 1407332 | 1417368 |
|  |  | 1969813 | 1972066 |
|  |  | 2263685 | 2443144 |
|  |  | 2515121 | 2516296 |
|  |  | 2534145 | 2535551 |
|  |  | 2744776 | 2770703 |
|  |  | 2836001 | 2836438 |
|  |  | 2967699 | 2980324 |
|  | Repetitive regions | 172706 | 175499 |
|  |  | 308419 | 308619 |
|  |  | 359051 | 364205 |
|  |  | 364794 | 365353 |
|  |  | 365355 | 365984 |
|  |  | 381076 | 381635 |
|  |  | 381637 | 382266 |
|  |  | 527360 | 527563 |
|  |  | 527580 | 527776 |
|  |  | 528165 | 528537 |
|  |  | 607410 | 607520 |
|  |  | 607525 | 612590 |
|  |  | 683377 | 699638 |
|  |  | 740593 | 740875 |
|  |  | 740908 | 742082 |
|  |  | 750489 | 750597 |
|  |  | 821439 | 821644 |
|  |  | 822038 | 822636 |
|  |  | 854601 | 854949 |
|  |  | 855051 | 855309 |
|  |  | 857459 | 857807 |
|  |  | 857909 | 858167 |
|  |  | 1182049 | 1182400 |
|  |  | 1182436 | 1183287 |
|  |  | 1183299 | 1183410 |
|  |  | 1196434 | 1197949 |
|  |  | 1206656 | 1206795 |
|  |  | 1206896 | 1206999 |
|  |  | 1207289 | 1207407 |
|  |  | 1207571 | 1207704 |
|  |  | 2344092 | 2344225 |
|  |  | 2344374 | 2344492 |
|  |  | 2344767 | 2344870 |
|  |  | 2345007 | 2345146 |
|  |  | 2437777 | 2440572 |
|  |  | 2442713 | 2442928 |
|  |  | 2442930 | 2443156 |
|  |  | 2478740 | 2478953 |
|  |  | 2479018 | 2480344 |
|  |  | 2480418 | 2480866 |
|  |  | 2480907 | 2481070 |
|  |  | 2481072 | 2481184 |
|  |  | 2481792 | 2481895 |
|  |  | 2487631 | 2487844 |
|  |  | 2487909 | 2489235 |
|  |  | 2489309 | 2489757 |
|  |  | 2489798 | 2489961 |
|  |  | 2489963 | 2490075 |
|  |  | 2490683 | 2490786 |
|  |  | 2515149 | 2515306 |
|  |  | 2516012 | 2517460 |
|  |  | 2535296 | 2536470 |
|  |  | 2625664 | 2625772 |
|  |  | 2835783 | 2836158 |
|  |  | 2836407 | 2836603 |
|  |  | 2836627 | 2836830 |
|  |  | 3173105 | 3173327 |
|  |  | 3173899 | 3174666 |
|  |  | 3175676 | 3177451 |
|  |  | 3177962 | 3179836 |
|  |  | 3426189 | 3426989 |
|  |  | 3427221 | 3428021 |
| EUL 120 (ST42) | MGEs | 1 | 60757 |
|  |  | 355812 | 358377 |
|  |  | 457092 | 457967 |
|  |  | 697482 | 698935 |
|  |  | 730314 | 742107 |
|  |  | 759142 | 759678 |
|  |  | 827201 | 828769 |
|  |  | 964006 | 1053111 |
|  |  | 1456188 | 1457399 |
|  |  | 1654156 | 1655267 |
|  |  | 1798125 | 1800378 |
|  |  | 1952654 | 1965442 |
|  |  | 1987708 | 1990686 |
|  |  | 2113816 | 2267685 |
|  |  | 2492938 | 2514904 |
|  |  | 2690115 | 2691186 |
|  |  | 2733011 | 2760276 |
|  |  | 3013090 | 3013479 |
|  |  | 3060467 | 3061538 |
|  |  | 3426014 | 3429537 |
|  | Repetitive regions | 1 | 409 |
|  |  | 615 | 823 |
|  |  | 871 | 990 |
|  |  | 1054 | 1187 |
|  |  | 1259 | 14010 |
|  |  | 35494 | 35902 |
|  |  | 36104 | 36312 |
|  |  | 36358 | 36477 |
|  |  | 36539 | 36672 |
|  |  | 36742 | 44913 |
|  |  | 53337 | 53572 |
|  |  | 53574 | 53754 |
|  |  | 53756 | 54701 |
|  |  | 239400 | 242626 |
|  |  | 242628 | 242906 |
|  |  | 242908 | 244560 |
|  |  | 245149 | 246339 |
|  |  | 261431 | 262621 |
|  |  | 355783 | 358499 |
|  |  | 456965 | 457991 |
|  |  | 487118 | 492291 |
|  |  | 565861 | 585285 |
|  |  | 731316 | 732342 |
|  |  | 742215 | 742439 |
|  |  | 1456166 | 1457514 |
|  |  | 1654158 | 1654306 |
|  |  | 1654308 | 1654654 |
|  |  | 1654700 | 1654940 |
|  |  | 1654942 | 1655077 |
|  |  | 1655091 | 1655225 |
|  |  | 1655227 | 1655419 |
|  |  | 1800387 | 1800490 |
|  |  | 1883141 | 1883286 |
|  |  | 1957099 | 1958044 |
|  |  | 1987686 | 1989034 |
|  |  | 1989067 | 1990799 |
|  |  | 2495762 | 2497487 |
|  |  | 2498696 | 2498799 |
|  |  | 2500430 | 2502167 |
|  |  | 2690022 | 2690375 |
|  |  | 2690377 | 2691336 |
|  |  | 2736620 | 2736756 |
|  |  | 2736920 | 2737052 |
|  |  | 2748283 | 2748419 |
|  |  | 2748583 | 2748715 |
|  |  | 2776718 | 2776823 |
|  |  | 2777025 | 2777130 |
|  |  | 2953422 | 2953578 |
|  |  | 2954540 | 2955659 |
|  |  | 2956539 | 2960108 |
|  |  | 3060374 | 3061058 |
|  |  | 3061060 | 3061690 |
|  |  | 3076741 | 3076995 |
|  |  | 3151745 | 3151934 |
|  |  | 3155573 | 3155762 |
|  |  | 3167974 | 3168119 |
|  |  | 3210132 | 3210940 |
|  |  | 3211167 | 3211975 |
|  |  | 3425957 | 3430562 |
| H044120014 (ST62) | MGEs | 171309 | 236885 |
|  |  | 500116 | 501210 |
|  |  | 578399 | 579595 |
|  |  | 936356 | 936892 |
|  |  | 1139075 | 1265997 |
|  |  | 1306592 | 1307845 |
|  |  | 1326070 | 1327474 |
|  |  | 1356934 | 1359926 |
|  |  | 1368895 | 1369989 |
|  |  | 1840061 | 1841465 |
|  |  | 2031174 | 2032575 |
|  |  | 2181330 | 2187802 |
|  |  | 2337326 | 2420261 |
|  |  | 2531094 | 2532498 |
|  |  | 2714961 | 2800869 |
|  |  | 3025389 | 3030927 |
|  |  | 3412308 | 3413712 |
|  | Repetitive regions | 43308 | 43659 |
|  |  | 43749 | 44192 |
|  |  | 44263 | 44377 |
|  |  | 221637 | 221748 |
|  |  | 316178 | 316285 |
|  |  | 316299 | 316409 |
|  |  | 316459 | 316631 |
|  |  | 361237 | 362492 |
|  |  | 378427 | 379527 |
|  |  | 418745 | 420510 |
|  |  | 420645 | 423918 |
|  |  | 424507 | 424726 |
|  |  | 424728 | 425039 |
|  |  | 425041 | 425207 |
|  |  | 425209 | 425375 |
|  |  | 425377 | 425699 |
|  |  | 440790 | 441009 |
|  |  | 441011 | 441322 |
|  |  | 441324 | 441490 |
|  |  | 441492 | 441658 |
|  |  | 441660 | 441982 |
|  |  | 500096 | 501314 |
|  |  | 663932 | 669104 |
|  |  | 742331 | 765181 |
|  |  | 891347 | 891461 |
|  |  | 891844 | 892246 |
|  |  | 945355 | 945466 |
|  |  | 946062 | 946177 |
|  |  | 997717 | 997943 |
|  |  | 1297304 | 1298479 |
|  |  | 1306451 | 1306753 |
|  |  | 1306756 | 1308131 |
|  |  | 1325927 | 1326704 |
|  |  | 1326758 | 1327813 |
|  |  | 1368875 | 1370093 |
|  |  | 1458408 | 1458635 |
|  |  | 1661021 | 1661157 |
|  |  | 1730085 | 1731315 |
|  |  | 1839975 | 1842085 |
|  |  | 1882338 | 1883397 |
|  |  | 2031141 | 2032825 |
|  |  | 2127264 | 2128547 |
|  |  | 2147695 | 2149152 |
|  |  | 2181298 | 2181558 |
|  |  | 2181560 | 2181712 |
|  |  | 2181714 | 2181929 |
|  |  | 2181931 | 2182589 |
|  |  | 2237462 | 2238859 |
|  |  | 2293902 | 2294918 |
|  |  | 2405818 | 2405952 |
|  |  | 2406054 | 2406314 |
|  |  | 2406316 | 2406468 |
|  |  | 2406470 | 2406685 |
|  |  | 2406687 | 2407345 |
|  |  | 2442174 | 2443374 |
|  |  | 2443469 | 2444469 |
|  |  | 2450488 | 2450654 |
|  |  | 2452057 | 2452189 |
|  |  | 2452787 | 2452948 |
|  |  | 2453215 | 2453345 |
|  |  | 2459385 | 2459551 |
|  |  | 2460954 | 2461086 |
|  |  | 2461681 | 2461842 |
|  |  | 2462109 | 2462239 |
|  |  | 2468028 | 2468136 |
|  |  | 2468138 | 2468471 |
|  |  | 2468480 | 2469302 |
|  |  | 2469304 | 2469410 |
|  |  | 2469412 | 2469566 |
|  |  | 2472840 | 2472948 |
|  |  | 2472950 | 2473283 |
|  |  | 2473292 | 2474114 |
|  |  | 2474116 | 2474222 |
|  |  | 2474224 | 2474378 |
|  |  | 2482888 | 2482987 |
|  |  | 2530991 | 2532845 |
|  |  | 2714931 | 2715044 |
|  |  | 2766175 | 2766288 |
|  |  | 2787145 | 2787279 |
|  |  | 2790313 | 2791513 |
|  |  | 2861756 | 2862031 |
|  |  | 3006312 | 3006411 |
|  |  | 3027839 | 3027952 |
|  |  | 3028949 | 3029064 |
|  |  | 3030402 | 3031601 |
|  |  | 3046532 | 3047812 |
|  |  | 3060384 | 3060509 |
|  |  | 3117702 | 3117917 |
|  |  | 3224584 | 3224819 |
|  |  | 3227635 | 3234158 |
|  |  | 3412167 | 3413037 |
|  |  | 3413067 | 3414198 |
|  |  | 3482481 | 3482698 |
|  |  | 3482700 | 3483289 |
|  |  | 3483516 | 3483733 |
|  |  | 3483735 | 3484324 |
| Alcoy (ST578) | MGEs | 68252 | 87883 |
|  |  | 181578 | 230836 |
|  |  | 609680 | 648761 |
|  |  | 713383 | 714258 |
|  |  | 957527 | 958980 |
|  |  | 1163221 | 1331608 |
|  |  | 1366871 | 1369020 |
|  |  | 1404437 | 1405972 |
|  |  | 1566257 | 1567378 |
|  |  | 1951210 | 1953250 |
|  |  | 1975809 | 1976930 |
|  |  | 2077930 | 2080183 |
|  |  | 2206613 | 2207192 |
|  |  | 2240194 | 2253489 |
|  |  | 2275132 | 2276259 |
|  |  | 2401979 | 2404670 |
|  |  | 2415705 | 2416739 |
|  |  | 2421247 | 2421714 |
|  |  | 2446243 | 2447118 |
|  |  | 2486005 | 2509743 |
|  |  | 2684076 | 2685217 |
|  |  | 2756697 | 2786025 |
|  |  | 3003084 | 3016236 |
|  |  | 3166939 | 3168060 |
|  |  | 3196856 | 3197731 |
|  |  | 3327199 | 3328339 |
|  |  | 3380374 | 3387940 |
|  |  | 3411607 | 3416013 |
|  |  | 3441670 | 3442791 |
|  | Repetitive regions | 408773 | 413725 |
|  |  | 413782 | 413927 |
|  |  | 414516 | 414676 |
|  |  | 414678 | 415612 |
|  |  | 430799 | 430959 |
|  |  | 430961 | 431895 |
|  |  | 690387 | 690497 |
|  |  | 690500 | 692117 |
|  |  | 692252 | 693057 |
|  |  | 693059 | 695557 |
|  |  | 713359 | 714383 |
|  |  | 772045 | 786054 |
|  |  | 774047 | 774161 |
|  |  | 774214 | 780482 |
|  |  | 780487 | 782171 |
|  |  | 966340 | 966496 |
|  |  | 1299798 | 1301846 |
|  |  | 1315695 | 1315938 |
|  |  | 1316687 | 1316792 |
|  |  | 1566162 | 1567389 |
|  |  | 1919285 | 1919391 |
|  |  | 1975799 | 1978249 |
|  |  | 1992976 | 1993850 |
|  |  | 1997483 | 1998357 |
|  |  | 2249330 | 2250620 |
|  |  | 2262945 | 2263070 |
|  |  | 2275108 | 2277151 |
|  |  | 2446116 | 2448162 |
|  |  | 2470939 | 2471621 |
|  |  | 2471623 | 2472333 |
|  |  | 2472335 | 2472529 |
|  |  | 2472531 | 2473605 |
|  |  | 2473607 | 2473902 |
|  |  | 2473904 | 2474091 |
|  |  | 2474290 | 2474511 |
|  |  | 2479839 | 2480521 |
|  |  | 2480523 | 2481233 |
|  |  | 2481235 | 2481429 |
|  |  | 2481431 | 2482505 |
|  |  | 2482507 | 2482802 |
|  |  | 2482804 | 2482991 |
|  |  | 2483190 | 2483411 |
|  |  | 2510167 | 2510274 |
|  |  | 2668380 | 2668580 |
|  |  | 2668631 | 2668831 |
|  |  | 2683982 | 2684989 |
|  |  | 2684991 | 2685177 |
|  |  | 2756963 | 2757078 |
|  |  | 2757687 | 2757812 |
|  |  | 2757814 | 2758321 |
|  |  | 2758323 | 2758704 |
|  |  | 2781518 | 2781623 |
|  |  | 2781795 | 2782038 |
|  |  | 2975051 | 2975175 |
|  |  | 2985013 | 2985112 |
|  |  | 3014335 | 3014448 |
|  |  | 3015445 | 3016793 |
|  |  | 3044504 | 3044660 |
|  |  | 3100292 | 3100400 |
|  |  | 3166844 | 3169295 |
|  |  | 3196730 | 3198777 |
|  |  | 3211309 | 3211454 |
|  |  | 3212855 | 3217033 |
|  |  | 3327105 | 3328111 |
|  |  | 3328113 | 3328299 |
|  |  | 3441576 | 3444026 |
|  |  | 3466052 | 3466794 |
|  |  | 3467087 | 3467829 |
