# Supplementary material for: Dynamics and impact of homologous recombination on the evolution of Legionella pneumophila
Source: PLoS Genet. 2017 Jun 26;13(6):e1006855. doi: 10.1371/journal.pgen.1006855 (PMC5507463; doi:10.1371/journal.pgen.1006855)
Supplement: S3 Table — (DOCX) [file pgen.1006855.s003.docx]

**S3 Table**. Genes in recombination hotspots in the six major disease-associated STs.

| **ST** | **Hotspot** | **Gene (named according to reference genome used for the ST and the Paris genome)** | **No. recombination events (predicted by Gubbins)** | **Product/function** |
| --- | --- | --- | --- | --- |
| ST1 | 1 | *lpp0019* | 4 | hypothetical protein. Similar to Legionella zinc metalloproteinase precursor |
|  |  | *lpp0020* | 4 | hypothetical protein. Putative integral membrane protein |
|  |  | *lpp0021* | 4 | hypothetical protein. Similar to conserved hypothetical protein |
|  |  | *lpp0022* | 5 | hypothetical protein. Similar to conserved hypothetical protein |
|  |  | *lpp0023* | 4 | hypothetical protein. Putative membrane protein |
|  |  | *lpp0024* | 4 | hemin binding protein |
|  | 2 | *lpp0356* | 4 | hypothetical protein. Protein with ankyrin motif |
|  | 3 | *lpp0819* | 4 | N-acylglucosamine 2-epimerase |
|  |  | *lpp0820* | 4 | hypothetical protein. Similar to acetyl transferase |
|  |  | *lpp0821* | 4 | hypothetical protein. Similar to polysaccharide biosynthesis protein |
|  |  | *lpp0822* | 4 | dTDP-4-dehydrorhamnose 3,5-epimerase |
|  |  | *lpp0823* | 4 | dTDP-4-keto-L-rhamnose reductase |
|  |  | *lpp0824* | 4 | dTDP-D-glucose 4,6-dehydratase |
|  |  | *lpp0825* | 5 | glucose-6-phosphate isomerase |
|  |  | *lpp0826* | 5 | glucose-1-phosphate thymidylyltransferase |
|  |  | *lpp0827* | 5 | hypothetical protein. Similar to NAD dependent epimerase/dehydratase family protein |
|  |  | *lpp0828* | 7 | alpha-N-acetylglucosaminyltransferase |
|  |  | *lpp0829a* | 7 | hypothetical protein |
|  |  | *lpp0829b* | 7 | hypothetical protein |
|  |  | *lpp0829c* | 7 | hypothetical protein |
|  |  | *lpp0830* | 6 | hypothetical protein |
|  | 4 | *lpp0961* | 4 | hypothetical protein. Similar to conserved hypothetical protein |
|  |  | *lpp0962* | 4 | hypothetical protein |
|  |  | *lpp0963* | 4 | hypothetical protein |
|  | 5 | *lpp1640* | 4 | hypothetical protein |
|  |  | *lpp1641* | 4 | hypothetical protein, alpha-amylase |
|  |  | *lpp1642* | 3 | hypothetical protein |
|  |  | *lpp1643* | 4 | hypothetical protein, alpha-amylase |
|  |  | *lpp1644* | 4 | Phosphoribosylglycinamide formyltransferase |
|  |  | *lpp1645* | 4 | Phosphoribosylamine-glycine ligase |
|  | 6 | *lpp1761* | 7 | hypothetical protein |
|  |  | *lpp1762* | 8 | hypothetical protein |
|  |  | *lpp1763* | 13 | alanyl-tRNA synthetase |
|  |  | *lpp1764* | 12 | Regulatory protein RecX |
|  |  | *lpp1765* | 13 | RecA protein |
|  |  | *lpp1766* | 14 | hypothetical protein |
|  |  | *lpp1767* | 15 | hypothetical protein |
|  |  | *lpp1768* | 17 | DNA mismatch repair protein MutS |
|  |  | *lpp1769* | 18 | hypothetical protein |
|  |  | *lpp1770* | 25 | hypothetical protein |
|  |  | *lpp1771* | 27 | hypothetical protein. Similar to delta-aminolevulinic acid dehydratases (porphobilinogen synthase) |
|  |  | *lpp1772* | 25 | hypothetical protein |
|  |  | *lpp1773* | 25 | hypothetical protein. Similar to long-chain fatty acid transport protein |
|  |  | *lpp1774* | 24 | hypothetical protein. Similar to diaminopimelate decarboxylase, aspartate kinase (fusion of lysA and lysC) |
|  |  | *lpp1775* | 19 | hypothetical protein. Similar to UvrD/REP helicase family protein |
|  |  | *lpp1776* | 15 | hypothetical protein. Similar to unknown protein |
|  |  | *lpp1777* | 14 | hypothetical protein. Similar to conserved hypothetical protein |
|  |  | *lpp1778* | 14 | Hydrogen peroxide-inducible genes activator |
|  |  | *lpp1779* | 13 | hypothetical protein. Similar to major facilitator family transporter |
|  |  | *lpp1780* | 13 | hypothetical protein |
|  |  | *lpp1781* | 9 | hypothetical protein. Similar to tetraacyldisaccharide 4'-kinase |
|  |  | *lpp1782* | 9 | lipid A export ATP-binding/permease protein MsbA |
|  |  | *lpp1783* | 8 | hypothetical protein |
|  |  | *lpp1784* | 9 | dihydroorotate dehydrogenase |
|  |  | *lpp1785* | 8 | hypothetical protein. Predicted transmembrane protein |
|  |  | *lpp1786* | 7 | hypothetical protein. Similar to conserved hypothetical protein |
|  |  | *lpp1787* | 6 | hypothetical protein. Similar to acyl-CoA dehydrogenase |
|  |  | *lpp1788* | 5 | hypothetical protein. Similar to acetyl-CoA acetyltransferase |
|  |  | *lpp1789* | 5 | hypothetical protein. |
|  |  | *lpp1790* | 5 | hypothetical protein. Similar to Acetyl/propionyl-CoA carboxylase, beta subunit |
|  |  | *lpp1791* | 5 | hypothetical protein. Similar to enoyl-CoA hydratase/isomerase |
|  |  | *lpp1792* | 5 | hypothetical protein. Similar to Acetyl/propionyl-CoA carboxylase, alpha subunit |
|  |  | *lpp1793* | 5 | hypothetical protein. Similar to hydroxymethylglutaryl-CoA lyase |
|  |  | *lpp1794* | 4 | hypothetical protein. Similar to acetyl-coenzyme A synthetase |
|  | 7 | *lpp2198* | 4 | hypothetical protein |
|  | 8 | *lpp2543* | 4 | hypothetical protein. Similar to glycosyl transferase |
|  |  | *lpp2544* | 5 | hypothetical protein. Similar to conserved hypothetical protein |
|  |  | *lpp2545* | 4 | hypothetical protein. Integral membrane protein, similar to metabolite efflux pump |
|  |  | *lpp2546* | 4 | SdbB protein (putative substrate of the Dot/Icm system). |
|  |  | *lpp2547* | 4 | hypothetical protein. Similar to hypothetical protein |
|  |  | *lpp2548* | 4 | hypothetical protein. Similar to conserved hypothetical protein |
|  |  | *lpp2549* | 4 | hypothetical protein. Protein with TPR motifs (protein-protein interaction motif) |
|  |  | *lpp2550* | 4 | phosphomannomutase |
|  | 9 | *lpp2595* | 5 | phospho-2-dehydro-3-deoxyheptonate aldolase |
|  |  | *lpp2596* | 5 | hypothetical protein. Similar to chorismate mutase (N-terminal part) |
|  |  | *lpp2597* | 5 | hypothetical protein. Similar to chorismate mutase (C-terminal part) |
|  |  | *lpp2598* | 5 | hypothetical protein. Similar to aspartate aminotransferase |
|  |  | *lpp2599* | 6 | hypothetical protein. Similar to tellurite resistance protein TehB |
|  |  | *lpp2600* | 5 | hypothetical protein |
|  |  | *lpp2601* | 5 | hypothetical protein. Similar to hemoglobin (protozoan/cyanobacterial globin family) |
|  |  | *lpp2602* | 5 | hypothetical protein. Similar to xylene monooxygenase |
|  |  | *lpp2603* | 5 | hypothetical protein. Similar to conserved hypothetical protein |
|  |  | *lpp2604* | 5 | hypothetical protein |
|  | 10 | *lpp2977* | 4 | hypothetical protein. Highly similar to peptide methionine sulfoxide reductase |
|  |  | *lpp2978* | 4 | hypothetical protein. Similar to hypothetical protein |
|  |  | *lpp2979* | 4 | hypothetical protein. Similar to copper amine oxidase |
| ST23 | 1 | *ST23_00399/*  *lpp0453* | 2 | protease HtpX homolog, heat shock protein HtpX, putative Zn-dependent protease, contains TPR repeats, peptidase family M48. |
|  |  | *ST23_00400/*  *lpp0454* | 2 | inner membrane transport permease yadH, daunorubicin resistance, ABC transporter membrane protein |
|  |  | *ST23_00401/*  *lpp0455* | 2 | daunorubicin/doxorubicin resistance ATP-binding protein DrrA, nodulation ABC transporter NodI, daunorubicin resistance ABC transporter |
|  |  | *ST23_00402/*  *lpp0456* | 2 | hypothetical protein |
|  |  | *ST23_00403/*  *lpp0457* | 2 | predicted proline hydroxylase |
|  |  | *ST23_00404/*  *lpp0458* | 2 | protein of unknown function DUF45 |
|  |  | *ST23_00405/*  *lpp0459* | 2 | hypothetical protein |
|  |  | *ST23_00406/*  *lpp0460* | 2 | hypothetical protein |
|  |  | *ST23_00407/*  *lpp0461* | 2 | Methylated-DNA--protein-cysteine methyltransferase |
|  |  | *ST23_00408/*  *lpp0462* | 2 | 50S ribosomal protein L19 |
|  |  | *ST23_00409/*  *lpp0463* | 2 | tRNA (guanine-N(1)-)-methyltransferase |
|  |  | *ST23_00410/*  *lpp0464* | 2 | 21K,16S rRNA-processing protein RimM |
|  |  | *ST23_00411/*  *lpp0465* | 2 | 30S ribosomal protein S16 |
|  |  | *ST23_00412/*  *lpp0466* | 2 | p48, signal recognition particle protein |
|  |  | *ST23_00413/*  *lpp0467* | 2 | hypothetical protein |
|  |  | *ST23_00414/*  *lpp0468* | 2 | hypothetical protein |
|  |  | *ST23_00415/*  *lpp0469* | 2 | ribulose-5-phosphate 4-epimerase and related epimerases and aldolases, ankyrin repeats (3 copies) |
|  |  | *ST23_00416/*  *lpp0470* | 2 | glutamate/gamma-aminobutyrate antiporter |
|  |  | *ST23_00417/*  *lpp0471* | 2 | hypothetical protein |
|  | 2 | *ST23_00625/*  *lpp0668* | 3 | Carboxylate-amine ligase YbdK |
|  |  | *ST23_00626/*  *lpp0669* | 3 | acetyl coenzyme A synthetase (ADP forming) |
|  | 3 | *ST23_00647/*  *lpp0690* | 2 | thymidine kinase |
|  |  | *ST23_00648/*  *lpp0691* | 2 | D-glucarate permease, regulatory protein UhpC, major facilitator superfamily |
|  | 4 | *ST23_00703/*  *lpp0748* | 2 | hypothetical protein |
|  |  | *ST23_00704/*  *lpp0749* | 2 | Proline--tRNA ligase |
|  |  | *ST23_00705/*  *lpp0750* | 2 | ribulose-5-phosphate 4-epimerase and related epimerases and aldolases, transient-receptor-potential calcium channel protein, ankyrin repeats (3 copies) |
|  |  | *ST23_00706/*  *lpp0751* | 2 | hypothetical protein |
|  |  | *ST23_00707/*  *lpp0752* | 2 | carbonic anhydrase, sulfate transporter family |
|  |  | *ST23_00708/*  *lpp0753* | 2 | tRNA 2-thiocytidine biosynthesis protein TtcA, predicted ATPase of the PP-loop superfamily implicated in cell cycle control |
|  |  | *ST23_00709/*  *lpp0754* | 2 | outer membrane protein tolC precursor, outer membrane efflux protein. |
|  |  | *ST23_00710/*  *lpp0755* | 2 | protein-L-isoaspartate O-methyltransferase |
|  |  | *ST23_00711/*  *lpp0756* | 2 | 2-amino-3-ketobutyrate coenzyme A ligase |
|  |  | *ST23_00712/*  *lpp0757* | 2 | L-threonine 3-dehydrogenase |
|  |  | *ST23_00713/*  *lpp0758* | 2 | uncharacterized ABC transporter, ChvD family |
|  | 5 | *ST23_01779/*  *lpp1768* | 2 | DNA mismatch repair protein mutS |
|  |  | *ST23_01780/*  *lpp1769* | 2 | outer membrane protein assembly factor YaeT |
|  |  | *ST23_01781/*  *lpp1770* | 3 | hypothetical protein (DUF490) |
|  | 6 | *ST23_01931/*  *lpp1925* | 2 | patatin-like phospholipase |
|  |  | *ST23_01932/*  *NA* | 2 | uncharacterized protein conserved in bacteria |
|  |  | *ST23_01933/*  *NA* | 2 | low-affinity cAMP phosphodiesterase |
|  |  | *ST23_01934/*  *NA* | 2 | hypothetical protein |
|  |  | *ST23_01935/*  *NA* | 2 | hypothetical protein |
|  |  | *ST23_01936/*  *lpp1930* | 2 | hypothetical protein |
|  |  | *ST23_01937/*  *lpp1931* | 2 | hypothetical protein |
|  |  | *ST23_01938/*  *lpp1932* | 2 | Sec7 domain-containing protein, RalF |
|  |  | *ST23_01939/*  *lpp1933* | 2 | putative lipid kinase BmrU |
|  |  | *ST23_01940/*  *lpp1934* | 2 | cyclic 3',5'-adenosine monophosphate phosphodiesterase |
|  |  | *ST23_01941/*  *lpp1935* | 2 | hypothetical protein |
|  |  | *ST23_01942/*  *lpp1936* | 2 | hypothetical protein |
|  |  | *ST23_01943/*  *lpp1937* | 2 | H+/gluconate symporter and related permeases |
|  |  | *ST23_01944/*  *lpp1938* | 2 | L-Ala-D/L-Glu epimerase |
|  |  | *ST23_01945/*  *lpp1939* | 2 | hypothetical protein |
|  |  | *ST23_01946/*  *lpp1941* | 2 | hypothetical protein |
|  |  | *ST23_01947/*  *lpp1942* | 2 | hypothetical protein |
|  | 7 | *ST23_01990/*  *lpp1977* | 2 | Tfp pilus assembly protein PilW |
|  | 8 | *ST23_02606/*  *lpp2517* | 2 | ankyrin repeats (3 copies) |
|  |  | *ST23_02607/*  *lpp2518* | 2 | hypothetical protein |
|  |  | *ST23_02608/*  *lpp2519* | 2 | hypothetical protein |
|  |  | *ST23_02609/*  *lpp2520* | 2 | putative acyltransferase, GNAT family |
|  |  | *ST23_02610/*  *lpp2521* | 2 | hypothetical protein |
|  |  | *ST23_02611/*  *lpp2522* | 2 | hypothetical protein |
|  |  | *ST23_02612/*  *lpp2523* | 2 | Response regulator rcp1 |
|  |  | *ST23_02613/*  *lpp2524* | 2 | phytochrome-like protein cph1, sensory histidine kinase AtoS, predicted periplasmic ligand-binding sensor domain, phosphate regulon sensor kinase PhoR, histidine kinase-, DNA gyrase B-, and HSP90-like ATPase. |
|  |  | *ST23_02614/*  *lpp2525* | 2 | heme NO binding |
|  |  | *ST23_02615/*  *lpp2526* | 2 | hypothetical protein |
|  |  | *ST23_02616/*  *lpp2527* | 2 | hypothetical protein |
|  |  | *ST23_02617/*  *lpp2528* | 2 | methyltransferase domain |
|  | 9 | *ST23_03044/*  *lpp2944* | 2 | hypothetical protein. |
|  |  | *ST23_03045/*  *lpp2945* | 2 | 7-cyano-7-deazaguanine synthase, queuosine biosynthesis protein QueC, asparagine synthase (glutamine-hydrolyzing) |
|  |  | *ST23_03046/*  *lpp2946* | 2 | alginate biosynthesis protein AlgA, mannose-1-phosphate guanyltransferase |
| ST37 | 1 | *ST37_01205/*  *lpp1189* | 2 | recombination-associated protein rdgC |
|  |  | *ST37_01206/*  *lpp1190* | 2 | potassium transport protein Kup |
| ST42 | 1 | *ST42_02559/*  *lpp2687* | 2 | cytosol aminopeptidase, multifunctional aminopeptidase A |
|  |  | *ST42_02560/*  *lpp2688* | 3 | hypothetical protein, integral membrane protein MviN |
|  |  | *ST42_02561/*  *lpp2689* | 3 | 30S ribosomal protein S20 |
|  |  | *ST42_02562/*  *lpp2690* | 3 | hypothetical protein |
|  |  | *ST42_02563/*  *lpp2691* | 3 | hypothetical protein |
|  |  | *ST42_02564/*  *lpp2692* | 3 | hypothetical protein, contains Sel1 repeat (EnhC) |
|  |  | *ST42_02565/*  *lpp2693* | 4 | hypothetical protein |
|  |  | *ST42_02566/*  *lpp2694* | 3 | hypothetical protein, L,D-transpeptidase catalytic domain |
|  |  | *ST42_02567/*  *lpp2695* | 3 | Cyclic di-GMP phosphodiesterase Gmr, RNase II stability modulator, MHYT domain (predicted integral membrane sensor domain) |
| ST62 | 1 | *ST62_00255/*  *lpp0262* | 2 | protein of unknown function (DUF2878). |
|  |  | *ST62_00256/*  *lpp0263* | 4 | hypothetical protein, EDD domain protein, DegV family |
|  |  | *ST62_00257/*  *lpp0264* | 4 | 2-(S)-hydroxypropyl-CoM dehydrogenase, 3-ketoacyl-(acyl-carrier-protein) reductase |
|  |  | *ST62_00258/*  *lpp0265* | 4 | deoxyribodipyrimidine photo-lyase-related protein |
|  |  | *ST62_00259/*  *lpp0266* | 2 | predicted membrane protein (DUF2177) |
|  |  | *ST62_00260/*  *lpp0267* | 2 | serine/threonine-protein kinase PrkC |
|  |  | *ST62_00261/*  *lpp0268* | 2 | hypothetical protein |
|  |  | *ST62_00262/*  *lpp0269* | 2 | hypothetical protein |
|  |  | *ST62_00263/*  *lpp0270* | 2 | TspO/MBR family |
|  |  | *ST62_00264/*  *lpp0271* | 2 | deoxyribodipyrimidine photo-lyase |
|  |  | *ST62_00265/*  *lpp0272* | 2 | inner membrane protein yohK, cytidylyltransferase, LrgB-like family |
|  |  | *ST62_00266/*  *lpp0273* | 2 | antiholin-like protein LrgA |
|  |  | *ST62_00267/*  *lpp0274* | 2 | Cyn operon transcriptional activator, DNA-binding transcriptional regulator CynR |
|  | 2 | *ST62_00277/*  *lpp0285* | 2 | outer membrane efflux protein |
|  | 3 | *ST62_00287/*  *lpp0305* | 2 | glutathione-dependent formaldehyde-activating enzyme |
|  |  | *ST62_00288/*  *lpp0306* | 2 | hypothetical protein |
|  |  | *ST62_00289/*  *lpp0307* | 2 | putative non-heme bromoperoxidase BpoC, acetoin dehydrogenase E2 subunit dihydrolipoyllysine-residue acetyltransferase, esterase/lipase,3-oxoadipate enol-lactonase, alpha/beta hydrolase family |
|  |  | *ST62_00290/*  *lpp0308* | 2 | betaine aldehyde dehydrogenase |
|  |  | *ST62_00291/*  *lpp0309* | 2 | 4-aminobutyrate aminotransferase GabT |
|  |  | *ST62_00292/*  *lpp0310* | 2 | hypothetical protein |
|  | 4 | *ST62_00754/*  *lpp0756* | 2 | 2-amino-3-ketobutyrate coenzyme A ligase |
|  |  | *ST62_00755/*  *lpp0757* | 2 | L-threonine 3-dehydrogenase |
|  |  | *ST62_00756/*  *lpp0758* | 2 | Uncharacterized ABC transporter, ChvD family |
|  |  | *ST62_00757/*  *lpp0759* | 2 | hypothetical protein, L,D-transpeptidase catalytic domain |
|  |  | *ST62_00758/*  *lpp0760* | 2 | predicted transporter component, YeeE/YedE family (DUF395) |
|  |  | *ST62_00759/*  *lpp0761* | 2 | predicted transporter component, YeeE/YedE family (DUF395) |
|  |  | *ST62_00760/*  *lpp0762* | 2 | outer membrane protein transport protein (OMPP1/FadL/TodX) |
|  |  | *ST62_00761/*  *lpp0763* | 2 | macrophage killing protein with similarity to conjugation protein |
|  |  | *ST62_00762/*  *lpp0764* | 2 | formimidoylglutamase |
|  |  | *ST62_00763/*  *lpp0765* | 2 | benzil reductase, short chain dehydrogenase |
|  |  | *ST62_00764/*  *lpp0766* | 2 | Imidazolonepropionase,imidazolonepropionase, cytosine deaminase and related metal-dependent hydrolases |
|  | 5 | *ST62_00817/*  *lpp0829* | 3 | hypothetical protein |
|  | 6 | *ST62_00823/*  *lpp0835* | 2 | hypothetical protein |
|  | 7 | *ST62_01733/*  *lpp1667* | 2 | hypothetical protein |
|  |  | *ST62_01734/*  *lpp1668* | 2 | DNA polymerase V subunit UmuC, nucleotidyltransferase/DNA polymerase involved in DNA repair |
|  |  | *ST62_01735/*  *lpp1669* | 2 | DNA polymerase V subunit UmuD, repressor LexA, peptidase S24-like. |
|  |  | *ST62_01736/*  *lpp1670* | 2 | carboxypeptidase G2 precursor, ArgE/DapE family, peptidase family M20/M25/M40. |
| ST578 | 1 | *lpa_01248/*  *lpp0880* | 2 | ATP binding protease component |
|  |  | *lpa_01249/*  *lpp0883* | 2 | lipopolysaccharide biosynthesis glycosyltransferase |
|  |  | *lpa_01251/*  *lpp0884* | 2 | O-antigen biosynthesis protein |
|  |  | *lpa_01252/*  *lpp0885* | 2 | hypothetical protein |
|  |  | *lpa_01253/*  *lpp0886* | 2 | romboid family protein |
|  |  | *lpa_01254/*  *lpp0887* | 2 | peptidase, M23/M37 family |
|  |  | *lpa_01255/*  *lpp0888* | 2 | exodeoxyribonuclease VII large subunit. |
|  |  | *lpa_01256/*  *lpp0889* | 2 | agglutination protein |
|  |  | *lpa_01258/*  *lpp0890* | 2 | predicted periplasmic protein |
|  |  | *lpa_01261/*  *lpp0891* | 2 | two component histidine kinase |
|  |  | *lpa_01262/*  *lpp0892* | 2 | hypothetical protein |
|  |  | *lpa_01264/*  *lpp0893* | 2 | flavin containing monooxygenase |
|  |  | *lpa_01265/*  *lpp0894* | 2 | short-chain dehydrogenase of various substrate specificities |
|  |  | *lpa_01266/*  *lpp0895* | 2 | indole-3-glycerol phosphate synthase |
|  |  | *lpa_01267/*  *lpp0896* | 2 | anthranilate phosphoribosyltransferase |
|  |  | *lpa_01268/*  *lpp0897* | 2 | anthranilate synthase component II |
|  |  | *lpa_01269/*  *lpp0898* | 2 | ABC-type transport system protein involved in lipoprotein release |
|  |  | *lpa_01270/*  *lpp0899* | 2 | putative protein conserved in bacteria |
|  |  | *lpa_01271/*  *lpp0900* | 2 | putative protein conserved in bacteria |
|  |  | *lpa_01272/*  *lpp0901* | 2 | hydrolase, HAD superfamily, low specificity phosphatase |
|  |  | *lpa_01273/*  *lpp0902* | 2 | polysialic acid capsule expression protein, predicted sugar phosphate isomerase involved in capsule formation |
|  | 2 | *lpa_01289/*  *lpp0914* | 2 | putative conserved protein |
|  | 3 | *lpa_02154/*  *lpp1435* | 2 | potassium efflux system protein KefA |
|  | 4 | *lpa_04035/*  *lpp2815* | 2 | glucose/sorbosone dehydrogenase |
|  |  | *lpa_04036/*  *lpp2816* | 2 | polyribonucleotide nucleotidyltransferase |
|  |  | *lpa_04037/*  *lpp2817* | 2 | small subunit ribosomal protein S15 |
|  |  | *lpa_04038/*  *lpp2818* | 2 | tRNA pseudouridine synthase B |
|  |  | *lpa_04039/*  *lpp2819* | 2 | ribosome-binding factor A |
|  |  | *lpa_04041/*  *lpp2820* | 2 | translation initiation factor 2 (GTPase) |
|  |  | *lpa_04042/*  *lpp2821* | 2 | N utilization substance protein A |
|  |  | *lpa_04043/*  *lpp2822* | 2 | putative protein conserved in bacteria |
|  |  | *lpa_04044/*  *lpp2823* | 2 | NADH dehydrogenase I chain N |
|  |  | *lpa_04046/*  *lpp2824* | 2 | NADH dehydrogenase I chain M |
|  |  | *lpa_04047/*  *lpp2825* | 2 | NADH dehydrogenase I chain L |
|  |  | *lpa_04048/*  *lpp2826* | 2 | NADH dehydrogenase I chain K |
|  |  | *lpa_04049/*  *lpp2827* | 2 | NADH dehydrogenase I chain J |
|  |  | *lpa_04050/*  *lpp2828* | 2 | NADH dehydrogenase I chain I |
|  |  | *lpa_04051/*  *lpp2829* | 2 | NADH dehydrogenase I chain H |
|  |  | *lpa_04052/*  *lpp2830* | 2 | NADH dehydrogenase I chain G |
|  |  | *lpa_04053/*  *lpp2831* | 2 | NADH dehydrogenase I chain F |
|  |  | *lpa_04055/*  *lpp2832* | 2 | NADH dehydrogenase I chain E |
|  |  | *lpa_04056/*  *lpp2833* | 2 | NADH dehydrogenase I chain D |
|  |  | *lpa_04057/*  *lpp2834* | 2 | NADH dehydrogenase I chain C |
|  |  | *lpa_04058/*  *lpp2835* | 2 | NADH dehydrogenase I chain B |
|  |  | *lpa_04060/*  *lpp2836* | 2 | NADH dehydrogenase I chain A |
|  |  | *lpa_04061/*  *lpp2837* | 2 | preprotein translocase SecG subunit |
|  |  | *lpa_04062/*  *lpp2838* | 2 | triosephosphate isomerase (TIM) |
|  |  | *lpa_04063/*  *lpp2839* | 2 | interaptin |
