# Supplementary material for: Dynamics and impact of homologous recombination on the evolution of Legionella pneumophila
Source: PLoS Genet. 2017 Jun 26;13(6):e1006855. doi: 10.1371/journal.pgen.1006855 (PMC5507463; doi:10.1371/journal.pgen.1006855)
Supplement: S4 Table — The number of events that have affected all 536 isolates used in the study are shown, as well as the numbers that have affected isolates belonging to the 6 STs of interest only. An extra column (ST1_blocks) was included with the number of recombinations obtained by post-processing the fastGEAR output by detecting recombinations using the phylogeny (see Methods), corresponding to the coloured blocks different from the background in S3B Fig, to make the results comparable with Gubbins. The script used to get these recombination counts can be found in https://users.ics.aalto.fi/~pemartti/fastGEAR/. (DOCX) [file pgen.1006855.s004.docx]

**S4 Table**. The number of “recent” recombination events predicted by fastGEAR in each of the genes from the prominent ST1 hotspot (*lpp1761*-*1794*), as well as 10 genes upstream and downstream of this region. The number of events that have affected all 536 isolates used in the study are shown, as well as the numbers that have affected isolates belonging to the 6 STs of interest only. An extra column (ST1 blocks) was included with the number of recombinations obtained by post-processing the fastGEAR output by detecting recombinations using the phylogeny (see Methods), corresponding to the coloured blocks different from the background in **S3B Fig**, to make the results comparable with Gubbins. The script used to get these recombination counts can be found in <https://users.ics.aalto.fi/~pemartti/fastGEAR/>.

| **Gene** | **No. of “recent” recombination events predicted by fastGEAR** | | | | | | | |
| --- | --- | --- | --- | --- | --- | --- | --- | --- |
|  | **Total (536 isolates)** | **ST1** | **ST1 blocks** | **ST23** | **ST37** | **ST42** | **ST62** | **ST578** |
| *lpp1751* | 1 | 0 | 0 | 0 | 0 | 0 | 0 | 0 |
| *lpp1752* | 0 | 0 | 0 | 0 | 0 | 0 | 0 | 0 |
| *lpp1753* | 0 | 0 | 0 | 0 | 0 | 0 | 0 | 0 |
| *lpp1754* | 1 | 0 | 0 | 0 | 0 | 0 | 0 | 0 |
| *lpp1755* | 0 | 0 | 2 | 0 | 0 | 0 | 0 | 0 |
| *lpp1756* | 2 | 0 | 2 | 0 | 0 | 0 | 0 | 0 |
| *lpp1757* | 0 | 0 | 0 | 0 | 0 | 0 | 0 | 0 |
| *lpp1758* | 2 | 0 | 0 | 0 | 0 | 0 | 0 | 0 |
| *lpp1759* | 3 | 0 | 0 | 0 | 0 | 0 | 0 | 0 |
| *lpp1760* | 1 | 0 | 0 | 0 | 0 | 0 | 0 | 0 |
| *lpp1761* | 7 | 3 | 8 | 0 | 1 | 1 | 0 | 1 |
| *lpp1762* | 0 | 0 | 0 | 0 | 0 | 0 | 0 | 0 |
| *lpp1763* | 1 | 0 | 2 | 0 | 0 | 0 | 0 | 0 |
| *lpp1764* | 2 | 0 | 2 | 0 | 0 | 0 | 0 | 0 |
| *lpp1765* | 2 | 0 | 8 | 0 | 0 | 0 | 0 | 0 |
| *lpp1766* | 7 | 0 | 3 | 0 | 0 | 0 | 0 | 0 |
| *lpp1767* | 4 | 1 | 3 | 3 | 0 | 0 | 1 | 0 |
| *lpp1768* | 3 | 3 | 9 | 0 | 0 | 0 | 0 | 0 |
| *lpp1769* | 6 | 0 | 5 | 5 | 0 | 0 | 0 | 0 |
| *lpp1770* | 15 | 7 | 28 | 1 | 0 | 0 | 1 | 0 |
| *lpp1771* | 12 | 6 | 24 | 0 | 0 | 0 | 0 | 2 |
| *lpp1772* | 0 | 0 | 5 | 0 | 0 | 0 | 0 | 0 |
| *lpp1773* | 7 | 0 | 7 | 0 | 0 | 0 | 0 | 0 |
| *lpp1774* | 7 | 5 | 10 | 0 | 0 | 0 | 0 | 0 |
| *lpp1775* | 16 | 1 | 4 | 0 | 0 | 0 | 0 | 0 |
| *lpp1776* | 1 | 0 | 4 | 0 | 0 | 0 | 0 | 0 |
| *lpp1777* | 1 | 0 | 1 | 0 | 0 | 0 | 0 | 0 |
| *lpp1778* | 2 | 0 | 1 | 0 | 0 | 0 | 0 | 0 |
| *lpp1779* | 1 | 0 | 1 | 0 | 0 | 0 | 0 | 0 |
| *lpp1780* | 1 | 0 | 1 | 0 | 0 | 0 | 0 | 0 |
| *lpp1781* | 1 | 0 | 0 | 0 | 0 | 0 | 0 | 0 |
| *lpp1782* | 1 | 0 | 3 | 0 | 0 | 0 | 0 | 0 |
| *lpp1783* | 4 | 0 | 2 | 0 | 0 | 0 | 0 | 0 |
| *lpp1784* | 2 | 1 | 3 | 0 | 0 | 0 | 0 | 0 |
| *lpp1785* | 1 | 0 | 1 | 0 | 0 | 0 | 0 | 0 |
| *lpp1786* | 2 | 0 | 0 | 0 | 0 | 0 | 0 | 0 |
| *lpp1787* | 0 | 0 | 1 | 0 | 0 | 0 | 0 | 0 |
| *lpp1788* | 2 | 0 | 1 | 0 | 0 | 0 | 0 | 0 |
| *lpp1789* | 1 | 0 | 1 | 0 | 0 | 0 | 0 | 0 |
| *lpp1790* | 1 | 0 | 0 | 0 | 0 | 0 | 0 | 0 |
| *lpp1791* | 1 | 2 | 2 | 0 | 0 | 1 | 0 | 0 |
| *lpp1792* | 2 | 0 | 1 | 0 | 0 | 0 | 0 | 0 |
| *lpp1793* | 1 | 0 | 1 | 0 | 0 | 0 | 0 | 0 |
| *lpp1794* | 3 | 0 | 1 | 0 | 0 | 0 | 0 | 0 |
| *lpp1795* | 0 | 0 | 0 | 0 | 0 | 0 | 0 | 0 |
| *lpp1796* | 3 | 0 | 0 | 0 | 0 | 0 | 0 | 0 |
| *lpp1797* | 1 | 3 | 4 | 0 | 0 | 0 | 0 | 0 |
| *lpp1798* | 1 | 0 | 0 | 0 | 0 | 0 | 0 | 0 |
| *lpp1799* | 5 | 0 | 1 | 0 | 0 | 0 | 0 | 0 |
| *lpp1800* | 0 | 0 | 0 | 0 | 0 | 0 | 0 | 0 |
| *lpp1801* | 5 | 0 | 4 | 0 | 0 | 0 | 0 | 0 |
| *lpp1802* | 1 | 0 | 0 | 0 | 0 | 0 | 0 | 0 |
| *lpp1803* | 2 | 1 | 1 | 0 | 0 | 0 | 0 | 0 |
| *lpp1804* | 0 | 0 | 0 | 0 | 0 | 0 | 0 | 0 |
| ***Total in hotspot (lpp1761-1794)*** | **117** | **29** | **143** | **9** | **1** | **2** | **2** | **3** |
