# Supplementary material for: Dynamics and impact of homologous recombination on the evolution of Legionella pneumophila
Source: PLoS Genet. 2017 Jun 26;13(6):e1006855. doi: 10.1371/journal.pgen.1006855 (PMC5507463; doi:10.1371/journal.pgen.1006855)
Supplement: S5 Table — ST: sequence type; Sg: serogroup; U/k: unknown; TA: travel-associated; NA: not applicable. (DOCX) [file pgen.1006855.s005.docx]

**S5 Table**. Additional *L. pneumophila* isolates (*n*=245) used in the inference of the recombination donors. ST: sequence type; Sg: serogroup; U/k: unknown; TA: travel-associated; NA: not applicable.

| **Isolate name** | **ST** | **Sg** | **Source** | **Country** | **Year** | **Known epidemiologic relatedness** | **Reference/**  **accession number** |
| --- | --- | --- | --- | --- | --- | --- | --- |
| Corby | 51 | 1 | clin | UK | 1982 | None | Gloeckner *et al.*  (2007) |
| Lorraine/  ST47_1 | 47 | 1 | clin | France | 2004 | None | Gomez-Valero *et al.* (2011) |
| Philadelphia-1 (ATCC 33152) | 36 | 1 | clin | USA | 1981 | None | Chien *et al.* (2004) |
| LC6774 | 154 | 1 | env | UK | 2003 | None | Underwood *et al.* (2013) |
| H093380153 | 179 | 1 | clin | UK | 2009 | None | Underwood *et al.* (2013) |
| H044500045 | 186 | 1 | clin | UK | 2004 | None | Underwood *et al.* (2013) |
| H075160080 | 188 | 1 | env | UK | 2007 | None | Underwood *et al.* (2013) |
| Lansing-3 | 336 | 15 | clin | USA | 1981 | None | Underwood *et al.* (2013) |
| RR08000517 | 337 | 4 | env | UK | 2007 | None | Underwood *et al.* (2013) |
| RR08000134 | 34 | 1 | env | UK | 2005 | None | Underwood *et al.* (2013) |
| RR08000760 | 376 | 4 | env | UK | 2006 | None | Underwood *et al.* (2013) |
| H100260089 | 44 | 1 | clin | UK | 2010 | None | Underwood *et al.* (2013) |
| H091960011 | 454 | 1 | env | UK | 2009 | None | Underwood *et al.* (2013) |
| H093620212 | 46 | 1 | clin | UK | 2009 | None | Underwood *et al.* (2013) |
| H065000139 | 54 | 1 | clin | UK | 2006 | None | Underwood *et al.* (2013) |
| H070840415 | 59 | 1 | clin | UK | 2007 | None | Underwood *et al.* (2013) |
| H090500162 | 611 | 1 | env | UK | 2009 | None | Underwood *et al.* (2013) |
| H074360710 | 68 | 6 | env | UK | 2007 | None | Underwood *et al.* (2013) |
| H091960009 | 707 | 4 | env | UK | 2009 | None | Underwood *et al.* (2013) |
| LC6451 | 78 | 1 | clin | UK | 2002 | None | Underwood *et al.* (2013) |
| H071260094 | 87 | 3 | clin | Spain | 2007 | None | Underwood *et al.* (2013) |
| H053260229 | 74 | 1 | clin | UK | 2005 | None | Underwood *et al.* (2013) |
| H043940028 | 84 | 1 | clin | UK | 2004 | None | Underwood *et al.* (2013) |
| Lens | 15 | 1 | clin | France | 2003 | None | Cazalet *et al.* (2004) |
| EUL 169 | 47 | 1 | clin | UK | 2006 | Related to ST47_5 and ST47_99 | Underwood *et al.* (2013) |
| H034700617 | 47 | 1 | clin | UK | 2003 | None | Reuter *et al.* (2013) |
| HL01313013 | 47 | 1 | clin | France | 2001 | None | David *et al.* (2016) |
| H064160534 | 47 | 1 | env | UK | 2006 | Related to ST47_2 and ST47_99 | David *et al.* (2016) |
| H043580159 | 47 | 1 | clin | UK | 2004 | None | David *et al.* (2016) |
| H043580160 | 47 | 1 | clin | UK | 2004 | None | David *et al.* (2016) |
| H043660021 | 47 | 1 | clin | UK | 2004 | None | David *et al.* (2016) |
| H043680663 | 47 | 1 | clin | UK | 2004 | None | David *et al.* (2016) |
| H043700021 | 47 | 1 | clin | UK | 2004 | None | David *et al.* (2016) |
| H043790008 | 47 | 1 | clin | UK | 2004 | None | David *et al.* (2016) |
| H052920051 | 47 | 1 | clin | UK | 2005 | None | David *et al.* (2016) |
| H053540106 | 47 | 1 | clin | UK | 2005 | None | David *et al.* (2016) |
| H063660005 | 47 | 1 | clin | UK | 2006 | Related to ST47_15 and ST47_21 | David *et al.* (2016) |
| H063660006 | 47 | 1 | clin | UK | 2006 | Related to ST47_14 and ST47_21 | David *et al.* (2016) |
| H063660009 | 47 | 1 | clin | UK | 2006 | None | David *et al.* (2016) |
| H063680006 | 47 | 1 | clin | UK | 2006 | Related to ST47_18 | David *et al.* (2016) |
| H063680007 | 47 | 1 | clin | UK | 2006 | Related to ST47_17 | David *et al.* (2016) |
| H063740003 | 47 | 1 | clin | UK | 2006 | None | David *et al.* (2016) |
| H063740018 | 47 | 1 | clin | UK | 2006 | None | David *et al.* (2016) |
| H063760006 | 47 | 1 | clin | UK | 2006 | Related to ST47_14 and ST47_15 | David *et al.* (2016) |
| H063780007 | 47 | 1 | clin | UK | 2006 | Related to ST47_23 | David *et al.* (2016) |
| H063780008 | 47 | 1 | clin | UK | 2006 | Related to ST47_22 | David *et al.* (2016) |
| H063860003 | 47 | 1 | clin | UK | 2006 | None | David *et al.* (2016) |
| H063960001 | 47 | 1 | clin | UK | 2006 | None | David *et al.* (2016) |
| LC5759 | 47 | 1 | clin | U/k (TA) | 2000 | None | David *et al.* (2016) |
| H070420013 | 47 | 1 | clin | UK | 2007 | None | David *et al.* (2016) |
| LC5822 | 47 | 1 | clin | UK | 2001 | None | David *et al.* (2016) |
| H040260015 | 47 | 1 | clin | UK | 2004 | None | David *et al.* (2016) |
| H055140095 | 47 | 1 | clin | UK | 2006 | None | David *et al.* (2016) |
| H060780053 | 47 | 1 | clin | UK | 2006 | None | David *et al.* (2016) |
| H061120064 | 47 | 1 | clin | UK | 2006 | None | David *et al.* (2016) |
| H062840608 | 47 | 1 | clin | UK | 2006 | None | David *et al.* (2016) |
| H062940111 | 47 | 1 | clin | UK | 2006 | None | David *et al.* (2016) |
| H064320006 | 47 | 1 | clin | UK | 2006 | None | David *et al.* (2016) |
| H064280005 | 47 | 1 | clin | UK | 2006 | None | David *et al.* (2016) |
| H064380002 | 47 | 1 | clin | UK | 2006 | None | David *et al.* (2016) |
| H064380001 | 47 | 1 | clin | UK | 2006 | None | David *et al.* (2016) |
| H064560527 | 47 | 1 | clin | UK | 2006 | None | David *et al.* (2016) |
| H064660638 | 47 | 1 | clin | UK | 2006 | None | David *et al.* (2016) |
| H070160015 | 47 | 1 | clin | UK | 2007 | None | David *et al.* (2016) |
| H071120010 | 47 | 1 | clin | UK | 2007 | None | David *et al.* (2016) |
| H071360036 | 47 | 1 | clin | UK | 2007 | None | David *et al.* (2016) |
| H072740002 | 47 | 1 | clin | UK | 2007 | None | David *et al.* (2016) |
| H073000045 | 47 | 1 | clin | UK | 2007 | None | David *et al.* (2016) |
| H073380007 | 47 | 1 | clin | UK | 2007 | None | David *et al.* (2016) |
| H073600182 | 47 | 1 | clin | UK | 2007 | None | David *et al.* (2016) |
| H073640185 | 47 | 1 | clin | UK | 2007 | None | David *et al.* (2016) |
| H074960018 | 47 | 1 | clin | UK | 2008 | None | David *et al.* (2016) |
| H080780059 | 47 | 1 | clin | UK | 2008 | None | David *et al.* (2016) |
| H053840008 | 47 | 1 | clin | UK | 2004 | None | David *et al.* (2016) |
| H072520002 | 47 | 1 | clin | UK | 2007 | None | David *et al.* (2016) |
| H081340222 | 47 | 1 | clin | UK | 2007 | None | David *et al.* (2016) |
| H082520613 | 47 | 1 | clin | UK | 2008 | None | David *et al.* (2016) |
| H083120262 | 47 | 1 | clin | UK | 2008 | None | David *et al.* (2016) |
| H083620580 | 47 | 1 | clin | UK | 2008 | None | David *et al.* (2016) |
| H083960064 | 47 | 1 | clin | UK | 2008 | None | David *et al.* (2016) |
| H084620118 | 47 | 1 | clin | UK | 2008 | None | David *et al.* (2016) |
| H090140214 | 47 | 1 | clin | UK | 2009 | None | David *et al.* (2016) |
| H090440226 | 47 | 1 | clin | UK | 2009 | None | David *et al.* (2016) |
| H040960441 | 47 | 1 | clin | UK | 2004 | None | David *et al.* (2016) |
| H041120007 | 47 | 1 | clin | UK | 2004 | None | David *et al.* (2016) |
| H093480403 | 47 | 1 | clin | U/k (TA) | 2009 | None | David *et al.* (2016) |
| H094340202 | 47 | 1 | clin | UK | 2009 | None | David *et al.* (2016) |
| H095060125 | 47 | 1 | clin | UK | 2009 | None | David *et al.* (2016) |
| H100140151 | 47 | 1 | clin | UK | 2010 | None | David *et al.* (2016) |
| H100660110 | 47 | 1 | clin | UK | 2010 | None | David *et al.* (2016) |
| H100700025 | 47 | 1 | clin | UK | 2010 | None | David *et al.* (2016) |
| H103140121 | 47 | 1 | clin | UK | 2010 | None | David *et al.* (2016) |
| H103620160 | 47 | 1 | clin | UK | 2010 | None | David *et al.* (2016) |
| H103660126 | 47 | 1 | clin | UK | 2010 | None | David *et al.* (2016) |
| H103660121 | 47 | 1 | clin | UK | 2010 | None | David *et al.* (2016) |
| H104420240 | 47 | 1 | clin | UK | 2010 | None | David *et al.* (2016) |
| H110480273 | 47 | 1 | clin | UK | 2011 | None | David et al. (2016) |
| H112320437 | 47 | 1 | clin | UK | 2011 | None | David *et al.* (2016) |
| H112080616 | 47 | 1 | clin | UK | 2011 | None | David *et al.* (2016) |
| H112380374 | 47 | 1 | clin | UK | 2011 | None | David *et al.* (2016) |
| H120160499 | 47 | 1 | clin | UK | 2012 | None | David *et al.* (2016) |
| H120200371 | 47 | 1 | clin | UK | 2012 | None | David *et al.* (2016) |
| H105140391 | 47 | 1 | clin | UK | 2010 | None | David *et al.* (2016) |
| H121040204 | 47 | 1 | clin | UK | 2012 | None | David *et al.* (2016) |
| H121420445 | 47 | 1 | clin | UK | 2012 | None | David *et al.* (2016) |
| H102240357 | 47 | 1 | clin | UK | 2010 | None | David *et al.* (2016) |
| H122500497 | 47 | 1 | clin | UK | 2012 | None | David *et al.* (2016) |
| H122820408 | 47 | 1 | clin | U/k (TA) | 2012 | None | David *et al.* (2016) |
| H123620597 | 47 | 1 | clin | UK | 2012 | None | David *et al.* (2016) |
| H123840629 | 47 | 1 | clin | UK | 2012 | None | David *et al.* (2016) |
| H123940534 | 47 | 1 | clin | UK | 2012 | None | David *et al.* (2016) |
| H124920387 | 47 | 1 | clin | UK | 2012 | None | David *et al.* (2016) |
| H131340777 | 47 | 1 | clin | UK | 2013 | Related to ST47_92, ST47_93 and ST47_94 | David *et al.* (2016) |
| H131460248 | 47 | 1 | clin | UK | 2013 | None | David *et al.* (2016) |
| H131480353 | 47 | 1 | env | UK | 2013 | Related to ST47_90, ST47_93 and ST47_94 | David *et al.* (2016) |
| H131480354 | 47 | 1 | env | UK | 2013 | Related to ST47_90, ST47_92 and ST47_94 | David *et al.* (2016) |
| H131840211 | 47 | 1 | env | UK | 2013 | Related to ST47_90, ST47_92 and ST47_93 | David *et al.* (2016) |
| H132140863 | 47 | 1 | clin | UK | 2013 | None | David *et al.* (2016) |
| EUL 31 | 47 | 1 | clin | France | 1994 | None | David *et al.* (2016) |
| EUL 70 | 47 | 1 | clin | UK | 1996 | None | David *et al.* (2016) |
| EUL 168 | 47 | 1 | clin | UK | 2005 | None | David *et al.* (2016) |
| EUL 170 | 47 | 1 | env | UK | 2006 | Related to ST47_2 and ST47_5 | David *et al.* (2016) |
| LG12084002 | 47 | 1 | clin | France | 2012 | None | David *et al.* (2016) |
| LG12034018 | 47 | 1 | clin | France | 2012 | None | David *et al.* (2016) |
| LG11463009 | 47 | 1 | clin | France | 2011 | None | David *et al.* (2016) |
| LG11415002 | 47 | 1 | clin | France | 2011 | None | David *et al.* (2016) |
| LG11403003 | 47 | 1 | clin | France | 2011 | None | David *et al.* (2016) |
| LG10425016 | 47 | 1 | clin | France | 2010 | None | David *et al.* (2016) |
| LG10397001 | 47 | 1 | clin | France | 2010 | None | David *et al.* (2016) |
| LG09534017 | 47 | 1 | clin | France | 2009 | None | David *et al.* (2016) |
| LG09471012 | 47 | 1 | clin | France | 2009 | None | David *et al.* (2016) |
| LG08394013 | 47 | 1 | clin | France | 2008 | None | David *et al.* (2016) |
| LG08251002 | 47 | 1 | clin | France | 2008 | None | David *et al.* (2016) |
| HL07512016 | 47 | 1 | clin | France | 2007 | None | David *et al.* (2016) |
| HL07055011 | 47 | 1 | clin | France | 2007 | None | David *et al.* (2016) |
| HL06353025 | 47 | 1 | clin | France | 2006 | None | David *et al.* (2016) |
| HL05383032 | 47 | 1 | clin | France | 2005 | None | David *et al.* (2016) |
| HL05375017 | 47 | 1 | clin | France | 2005 | None | David *et al.* (2016) |
| HL04411050 | 47 | 1 | env | France | 2004 | None | David *et al.* (2016) |
| HL04284070 | 47 | 1 | clin | France | 2004 | None | David *et al.* (2016) |
| HL04075055 | 47 | 1 | clin | France | 2004 | None | David *et al.* (2016) |
| HL03503011 | 47 | 1 | clin | France | 2003 | None | David *et al.* (2016) |
| HL03443027 | 47 | 1 | clin | France | 2003 | None | David *et al.* (2016) |
| HL02392002 | 47 | 1 | clin | France | 2002 | None | David *et al.* (2016) |
| HL02274033 | 47 | 1 | clin | France | 2002 | None | David *et al.* (2016) |
| ATCC 43290 | 187 | 12 | clin | USA | U/k | None | Amaro *et al.* (2012) |
| HL06041035 | 734 | 1 | env | France | 2006 | None | Gomez-Valero *et al.* (2011) |
| Thunderbay | 187 | 6 | clin | Canada | U/k | None | Khan *et al.* (2013) |
| EUL 2 | 2 | 1 | clin | Switzerland | 1989 | None | David *et al.* (2016) |
| EUL 5 | 114 | 6 | clin | Switzerland | U/k | None | David *et al.* (2016) |
| EUL 7 | 18 | 1 | clin | Switzerland | 1992 | None | David *et al.* (2016) |
| EUL 18 | 26 | 1 | clin | Scotland | 1994 | None | David *et al.* (2016) |
| EUL 19 | 9 | 1 | clin | Scotland | 1994 | Related to EUL 22, 23 and 24 | David *et al.* (2016) |
| EUL 20 | 28 | 1 | clin | Scotland | 1995 | None | David *et al.* (2016) |
| EUL 22 | 9 | 1 | clin | Scotland | 1994 | Related to EUL 19, 23 and 24 | David *et al.* (2016) |
| EUL 23 | 9 | 1 | clin | Scotland | 1994 | Related to EUL 19, 22 and 24 | David *et al.* (2016) |
| EUL 24 | 9 | 1 | env | Scotland | 1994 | Related to EUL 19, 22 and 23 | David *et al.* (2016) |
| EUL 25 | 44 | 1 | clin | France | 1994 | None | David *et al.* (2016) |
| EUL 26 | 22 | 1 | clin | France | U/k | None | David *et al.* (2016) |
| EUL 30 | 38 | 1 | clin | France | U/k | None | David *et al.* (2016) |
| EUL 32 | 16 | 1 | clin | France | 1994 | None | David *et al.* (2016) |
| EUL 33 | 40 | 1 | clin | France | U/k | Related to EUL 34 and 35 | David *et al.* (2016) |
| EUL 34 | 40 | 1 | env | France | U/k | Related to EUL 33 and 35 | David *et al.* (2016) |
| EUL 35 | 40 | 1 | env | France | 1996 | Related to EUL 33 and 34 | David *et al.* (2016) |
| EUL 36 | 21 | 1 | clin | Italy | 1999 | None | David *et al.* (2016) |
| EUL 48 | 48 | 1 | clin | Spain | 1996 | Related to EUL 56 | David *et al.* (2016) |
| EUL 56 | 48 | 1 | clin | Spain | 1996 | Related to EUL 48 | David *et al.* (2016) |
| EUL 61 | 77 | 1 | env | Greece | 1989 | None | David *et al.* (2016) |
| EUL 64 | 77 | 1 | env | Greece | 1986 | None | David *et al.* (2016) |
| EUL 68 | 46 | 1 | clin | UK | 1995 | None | David *et al.* (2016) |
| EUL 72 | 4 | 1 | clin | UK | 1996 | None | David *et al.* (2016) |
| EUL 74 | 29 | 1 | clin | UK | 1995 | None | David *et al.* (2016) |
| EUL 81 | 53 | 1 | env | Denmark | 1994 | Related to EUL 96 | David *et al.* (2016) |
| EUL 83 | 50 | 1 | clin | Denmark | 1995 | None | David *et al.* (2016) |
| EUL 86 | 46 | 1 | clin | Denmark | 1995 | None | David *et al.* (2016) |
| EUL 91 | 63 | 1 | clin | Denmark | 1995 | None | David *et al.* (2016) |
| EUL 92 | 53 | 1 | clin | Denmark | 1991 | None | David *et al.* (2016) |
| EUL 96 | 53 | 1 | clin | Denmark | 1994 | Related to EUL 81 | David *et al.* (2016) |
| EUL 97 | 9 | 1 | clin | Sweden | 1994 | Related to EUL 107 | David *et al.* (2016) |
| EUL 98 | 9 | 1 | clin | Sweden | 1996 | None | David *et al.* (2016) |
| EUL 99 | 34 | 1 | clin | Sweden | 1995 | None | David *et al.* (2016) |
| EUL 100 | 59 | 1 | clin | Sweden | 1995 | None | David *et al.* (2016) |
| EUL 101 | 60 | 1 | clin | Sweden | 1994 | None | David *et al.* (2016) |
| EUL 102 | 59 | 1 | clin | Sweden | 1993 | None | David *et al.* (2016) |
| EUL 103 | 45 | 1 | clin | Sweden | 1993 | None | David *et al.* (2016) |
| EUL 107 | 9 | 1 | env | Sweden | 1994 | Related to EUL 97 | David *et al.* (2016) |
| EUL 111 | 25 | 1 | clin | Germany | 1981 | None | David *et al.* (2016) |
| EUL 118 | 36 | 1 | clin | Germany | 1989 | None | David *et al.* (2016) |
| EUL 126 | 27 | 1 | clin | UK | 1985 | Related to EUL 127 and 128 | ERR376691 |
| EUL 127 | 27 | 1 | clin | UK | 1985 | Related to EUL 126 and 128 | ERR376761 |
| EUL 128 | 27 | 1 | clin | UK | 1985 | Related to EUL 126 and 127 | ERR376699 |
| EUL 144 | 48 | 1 | env | UK | 2002 | None | ERR376768 |
| EUL 145 | 78 | 1 | env | UK | 2002 | Barrow outbreak | David *et al.* (2016) |
| EUL 148 | 1321 | 8 | env | Australia | 2003 | None | David *et al.* (2016) |
| EUL 149 | 83 | 1 | clin | UK | 2004 | None | David *et al.* (2016) |
| EUL 150 | 79 | 1 | clin | UK | 2003 | None | David *et al.* (2016) |
| EUL 152 | 80 | 5 | env | UK | 2004 | None | ERR352157 |
| EUL 153 | 68 | 6 | clin | UK | 1986 | Related to EUL 158 | David *et al.* (2016) |
| EUL 154 | 1326 | 8 | clin | UK | 1988 | Related to EUL 155 | David *et al.* (2016) |
| EUL 155 | 1326 | 8 | env | UK | 1988 | Related to EUL 154 | David *et al.* (2016) |
| EUL 158 | 68 | 6 | env | UK | 1986 | Related to EUL 153 | David *et al.* (2016) |
| EUL 161 | 75 | 1 | clin | UK | U/k | None | David *et al.* (2016) |
| EUL 162 | 85 | 1 | clin | UK | U/k | None | David *et al.* (2016) |
| EUL 163 | 73 | U/k | clin | Austria | U/k | None | David *et al.* (2016) |
| EUL 167 | 82 | 1 | clin | UK | U/k | None | David *et al.* (2016) |
| H073240536 | 1327 | 5 | clin | NA (cruise ship) | 2007 | Related to H073280012, H073340034  and H073340594 | David *et al.* (2016) |
| H073280012 | 1327 | 5 | env | NA (cruise ship) | 2007 | Related to H073240536,  H073340034  and H073340594 | David *et al.* (2016) |
| H073340034 | 1327 | 5 | clin | NA (cruise ship) | 2007 | Related to H073240536, H073280012  and H073340594 | David *et al.* (2016) |
| H073340594 | 1327 | 5 | clin | NA (cruise ship) | 2007 | Related to H073240536, H073280012 and H073340034 | David *et al.* (2016) |
| H092380261 | 109 | U/k | clin | UK | 2009 | Related to H092400768 | David *et al.* (2016) |
| H092400768 | 109 | U/k | env | UK | 2009 | Related to H092380261 | David *et al.* (2016) |
| H123640643 | 71 | 11 | clin | U/k | U/k | None | David *et al.* (2016) |
| LC6376 | 78 | 1 | env | UK | 2002 | Barrow outbreak | David *et al.* (2016) |
| LC6382 | 78 | 1 | env | UK | 2002 | Barrow outbreak | David *et al.* (2016) |
| LC6385 | 78 | 1 | env | UK | 2002 | Barrow outbreak | David *et al.* (2016) |
| LC6388 | 78 | 1 | env | UK | 2002 | Barrow outbreak | David *et al.* (2016) |
| LC6391 | 78 | 1 | env | UK | 2002 | Barrow outbreak | David *et al.* (2016) |
| LC6394 | 78 | 1 | env | UK | 2002 | Barrow outbreak | David *et al.* (2016) |
| LC6397 | 78 | 1 | clin | UK | 2002 | Barrow outbreak | David *et al.* (2016) |
| LC6406 | 78 | 1 | clin | UK | 2002 | Barrow outbreak | David *et al.* (2016) |
| LC6407 | 78 | 1 | clin | UK | 2002 | Barrow outbreak | David *et al.* (2016) |
| LC6408 | 78 | 1 | clin | UK | 2002 | Barrow outbreak | David *et al.* (2016) |
| LC6409 | 78 | 1 | clin | UK | 2002 | Barrow outbreak | David *et al.* (2016) |
| LC6410 | 78 | 1 | clin | UK | 2002 | Barrow outbreak | David *et al.* (2016) |
| LC6411 | 78 | 1 | clin | UK | 2002 | Barrow outbreak | David *et al.* (2016) |
| LC6412 | 78 | 1 | clin | UK | 2002 | Barrow outbreak | David *et al.* (2016) |
| LC6413 | 78 | 1 | clin | UK | 2002 | Barrow outbreak | David *et al.* (2016) |
| LC6416 | 78 | 1 | clin | UK | 2002 | Barrow outbreak | David *et al.* (2016) |
| LC6417 | 78 | 1 | clin | UK | 2002 | Barrow outbreak | David *et al.* (2016) |
| LC6418 | 78 | 1 | clin | UK | 2002 | Barrow outbreak | David *et al.* (2016) |
| ID_1885 | 1037 | U/k | env | Spain | 2004 | U/k | Sanchez-Buso *et al.* (2014) |
| ID_2423 | 1037 | U/k | env | Spain | 1999 | U/k | Sanchez-Buso *et al.* (2014) |
| ID_496053 | 1106 | U/k | clin | Spain | 2011 | U/k | Sanchez-Buso *et al.* (2014) |
| ID_918 | 1236 | U/k | U/k | Spain | U/k | U/k | Sanchez-Buso *et al.* (2014) |
| ID_3019 | 15 | U/k | clin | Spain | 2000 | U/k | Sanchez-Buso *et al.* (2014) |
| ID_479 | 171 | U/k | env | Spain | 2001 | U/k | Sanchez-Buso *et al.* (2014) |
| ID_2949 | 328 | U/k | env | Spain | 2000 | U/k | Sanchez-Buso *et al.* (2014) |
| ID_3164 | 51 | U/k | env | Spain | 2000 | U/k | Sanchez-Buso *et al.* (2014) |
| ID_3201 | 637 | U/k | env | Spain | 2000 | U/k | Sanchez-Buso *et al.* (2014) |
| ID_3215 | 637 | U/k | clin | Spain | 2000 | U/k | Sanchez-Buso *et al.* (2014) |
| ID_3216 | 637 | U/k | clin | Spain | 2000 | U/k | Sanchez-Buso *et al.* (2014) |
| ID_3238 | 637 | U/k | clin | Spain | 2000 | U/k | Sanchez-Buso *et al.* (2014) |
| ID_3334 | 637 | U/k | clin | Spain | 2000 | U/k | Sanchez-Buso *et al.* (2014) |
| ID_505237 | 637 | U/k | clin | Spain | 2011 | U/k | Sanchez-Buso *et al.* (2014) |
| ID_482 | 804 | U/k | env | Spain | 2001 | U/k | Sanchez-Buso *et al.* (2014) |
