# Supplementary figures and images for: Dynamics and impact of homologous recombination on the evolution of Legionella pneumophila
Source: PLoS Genet. 2017 Jun 26;13(6):e1006855. doi: 10.1371/journal.pgen.1006855 (PMC5507463; doi:10.1371/journal.pgen.1006855)

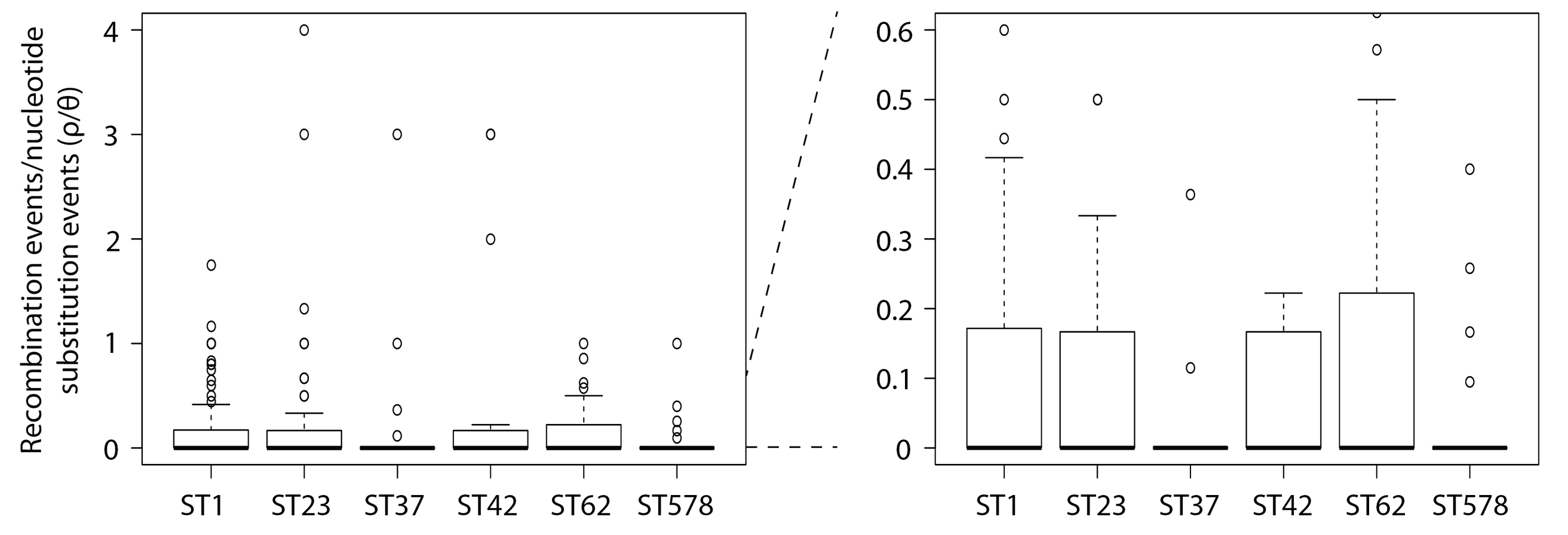

Supplement: S1 Fig — (TIF) [file pgen.1006855.s006.tif]

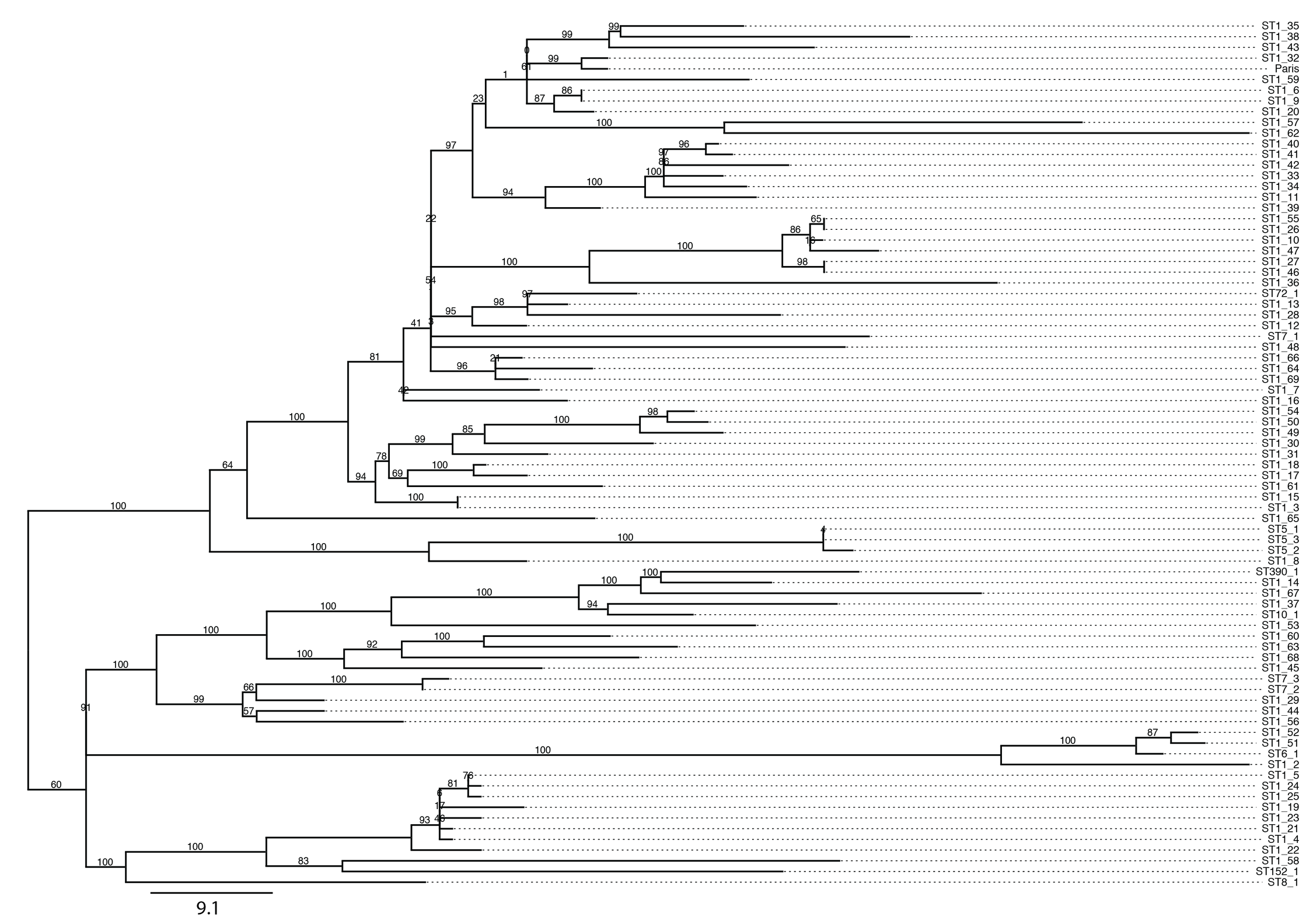

Supplement: S2 Fig — The scale shows the number of SNPs. (TIF) [file pgen.1006855.s007.tif]

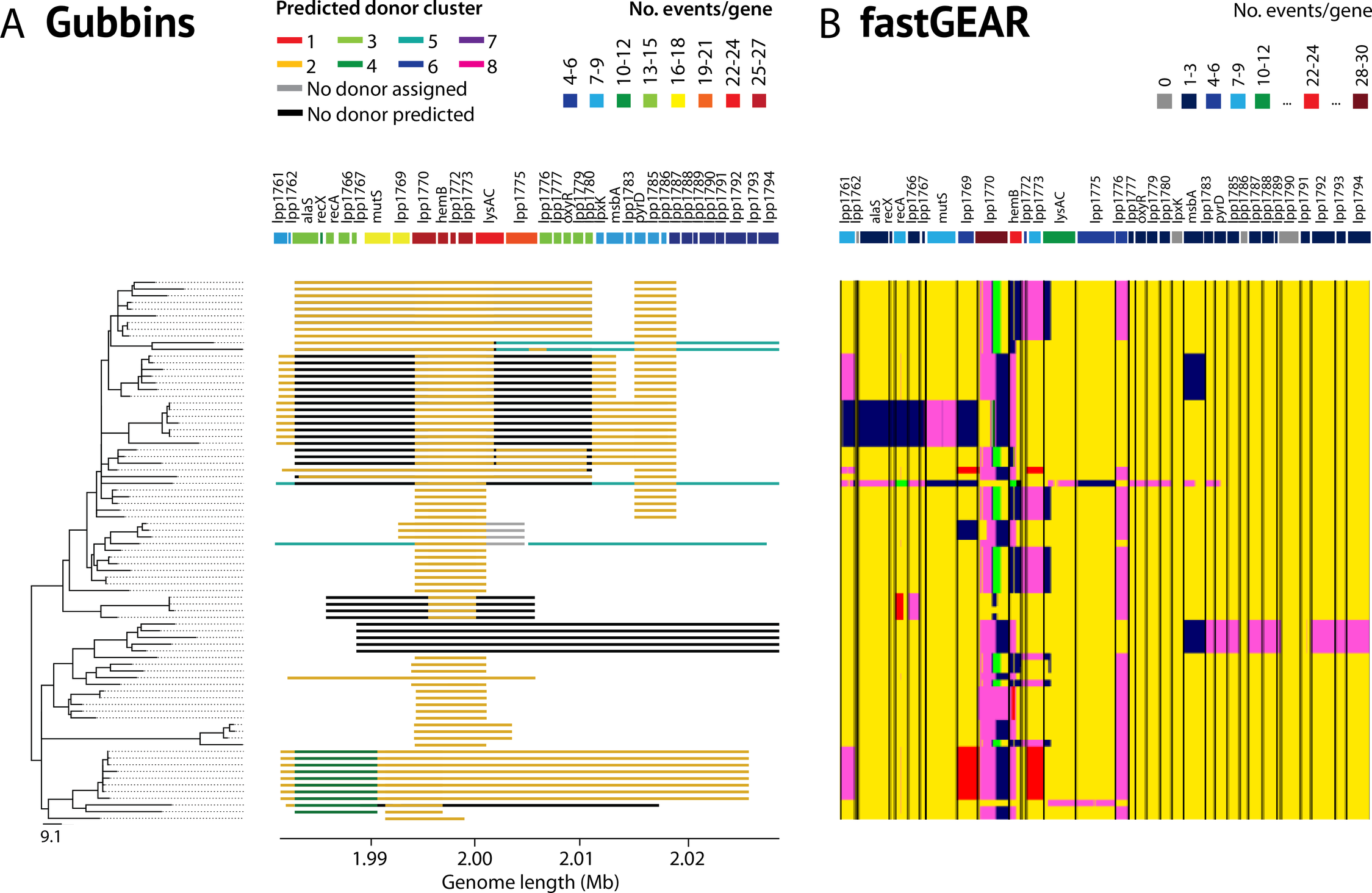

Supplement: S3 Fig — Recombination events predicted in the prominent ST1 hotspot as inferred by Gubbins (A) and fastGEAR (B). (A) was extracted from Fig 2 and shows the zoomed-in illustration of hotspot 6 in the ST1 lineage (lpp1761-lpp1794). The homologous recombination events are displayed as blocks and coloured according to the BAPS cluster from which they are predicted to be derived. The genes shown at the top of the figure are coloured by the number of times that they have been affected by a homologous recombination event, as predicted by Gubbins (see key at the top right). (B) shows regions of shared ancestry for this hotspot in the ST1 lineage, as predicted by fastGEAR. The genes at the top are coloured by the number of recombinations, corresponding to blocks of segments differing from the yellow background detected in this subset (see key at the top right). (TIF) [file pgen.1006855.s008.tif]

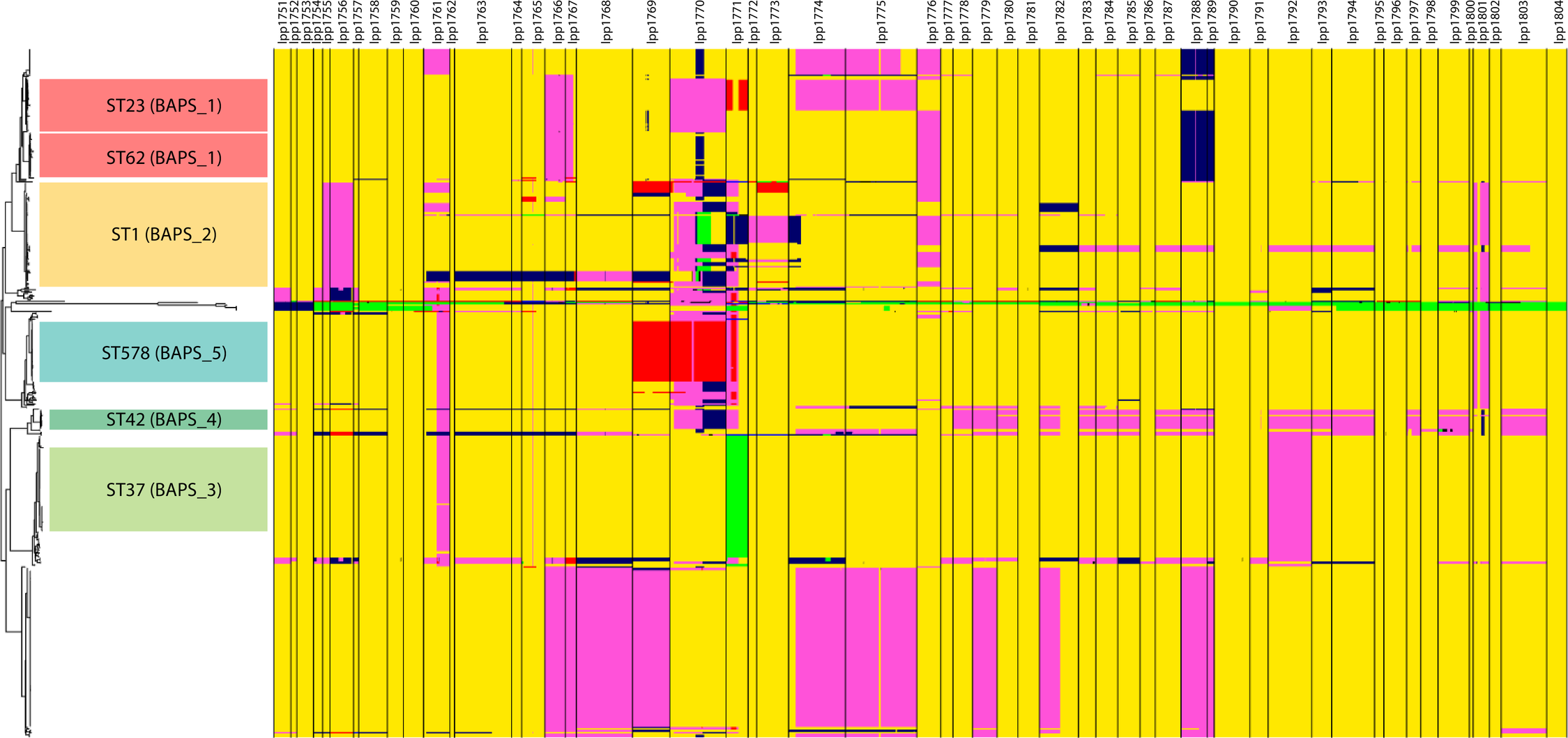

Supplement: S4 Fig — The left panel shows the maximum likelihood tree of the core genome alignment of the 536 L. pneumophila genomes included in the study. The main 6 STs are highlighted in the tree with the background colour representing their BAPS cluster (see Fig 3). FastGEAR output is shown per gene, with colours representing the donor lineages of both “recent” and “ancestral” recombination events. Lineage colours were reordered at different genes to optimize visualization as in [34]. (TIF) [file pgen.1006855.s009.tif]

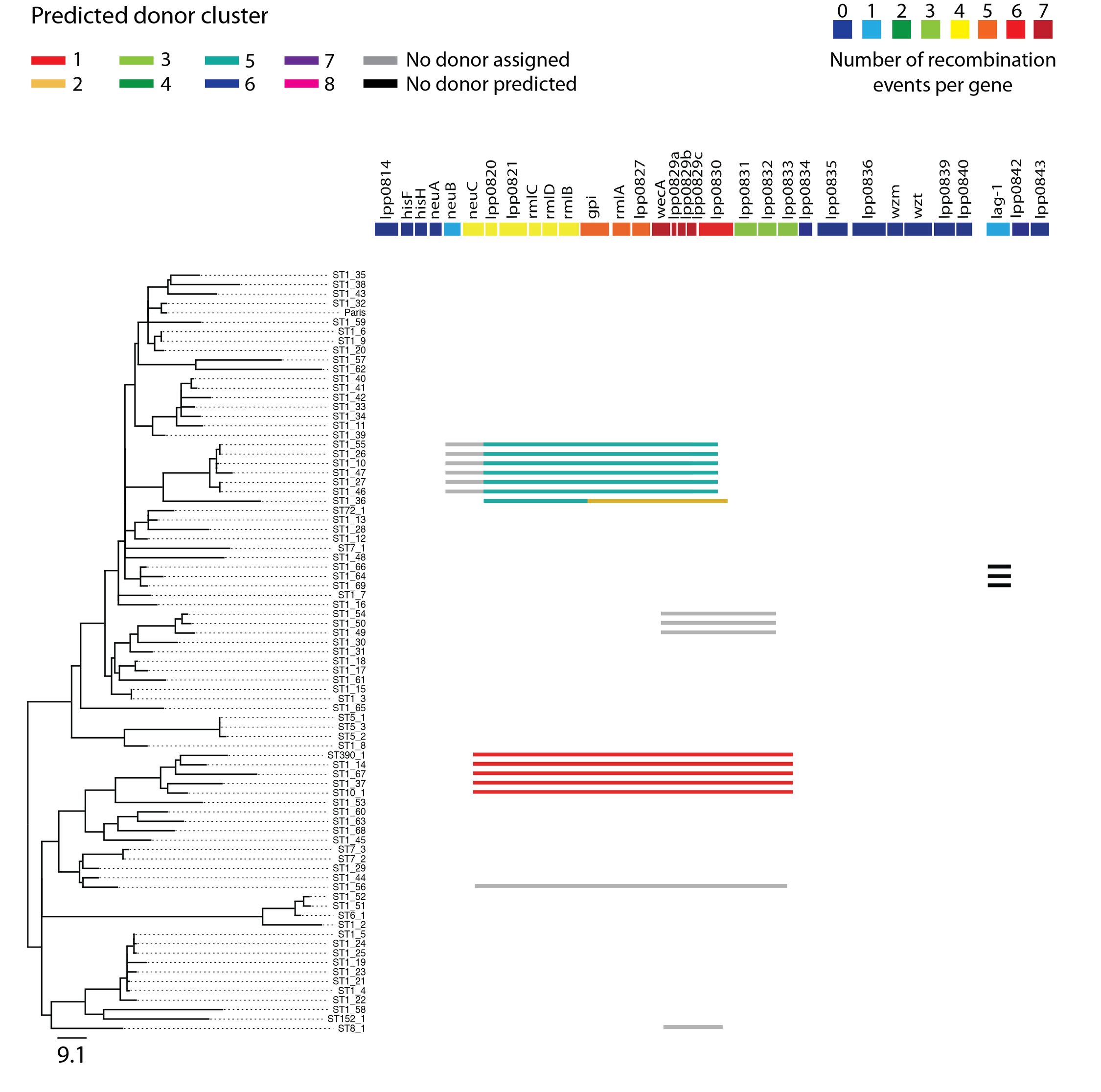

Supplement: S5 Fig — The recombination events are displayed as blocks, coloured according to the BAPS cluster from which they are predicted to be derived. The genes are shown at the top of the figure and coloured by the number of overlapping recombination regions. A maximum likelihood tree, constructed using only vertically inherited SNPs, is also shown on the left and the scale indicates the number of SNPs. (TIF) [file pgen.1006855.s010.tif]

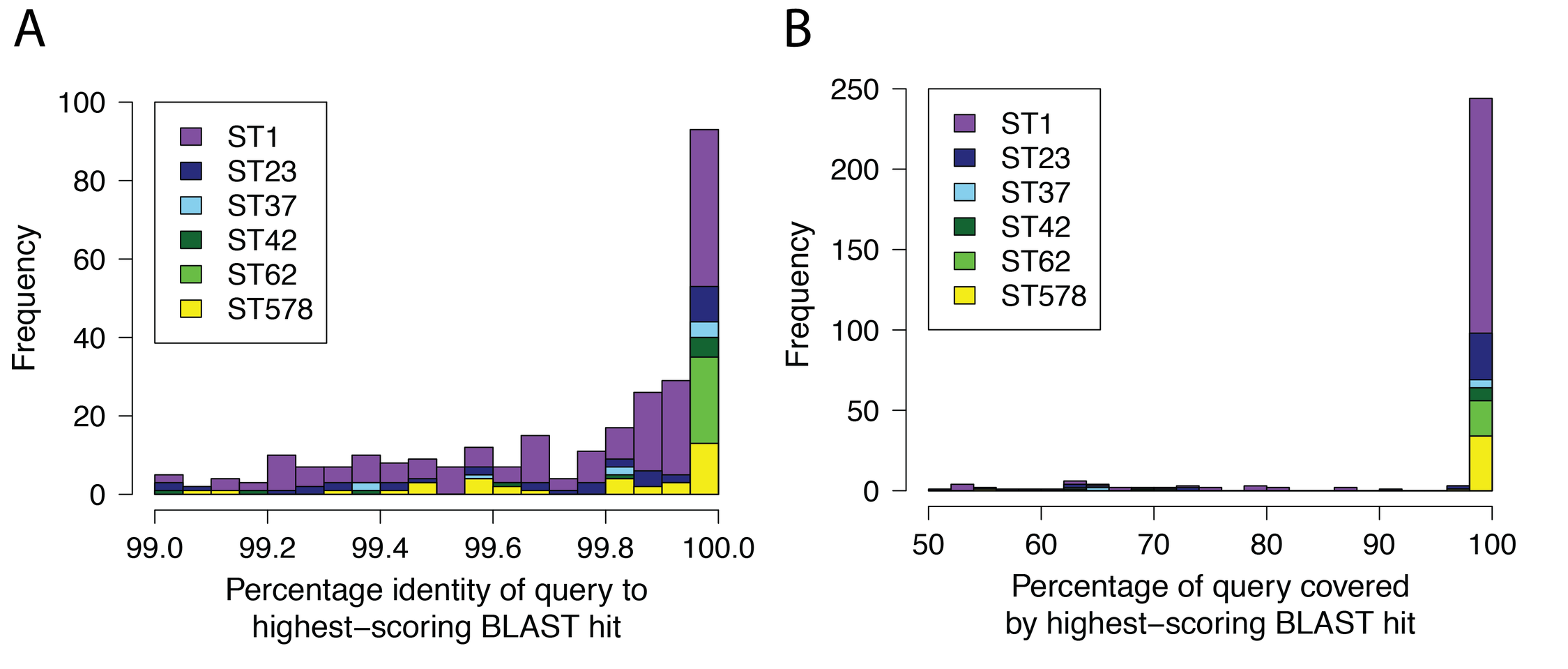

Supplement: S6 Fig — The percentage nucleotide identity of the recombination fragments to the highest-scoring BLAST hit (A) and the percentage length of the recombination fragment covered by the highest-scoring BLAST hit (B). (TIF) [file pgen.1006855.s011.tif]

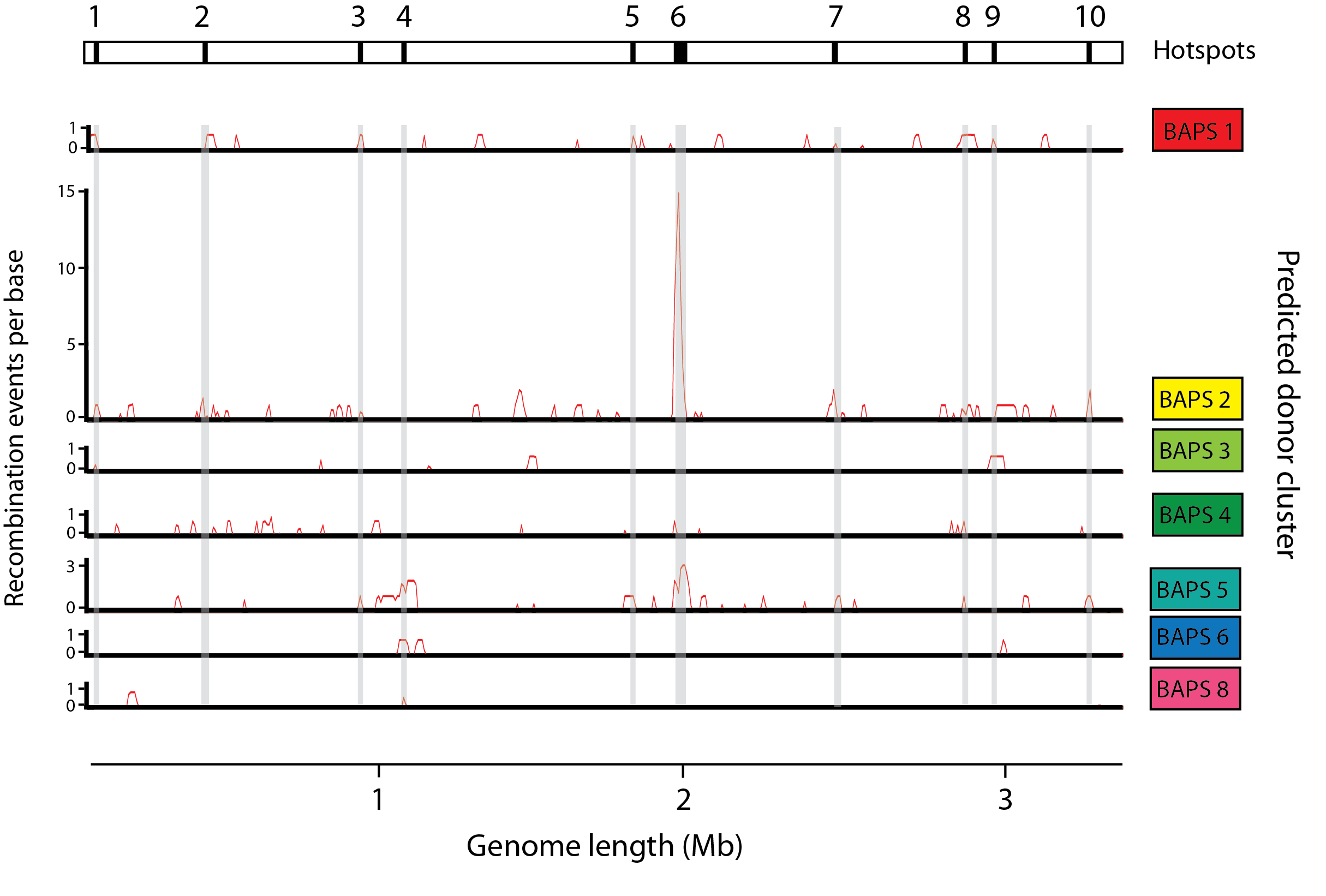

Supplement: S7 Fig — The vertical grey bars correspond to the recombination hotspots. (TIF) [file pgen.1006855.s012.tif]

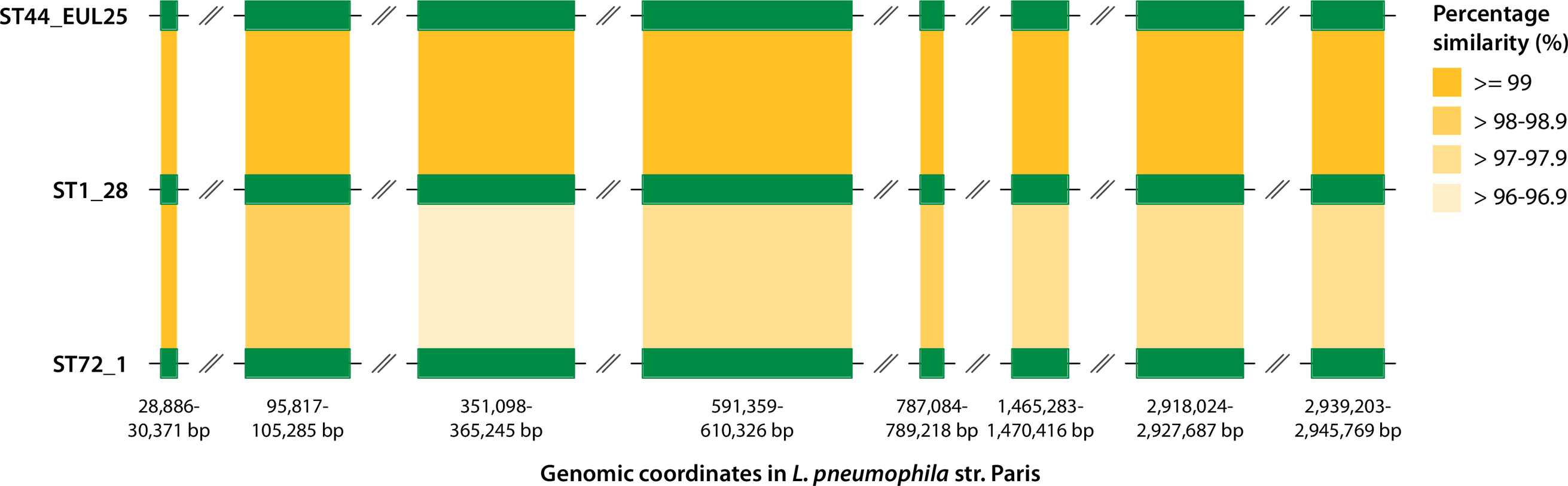

Supplement: S8 Fig — (TIF) [file pgen.1006855.s013.tif]
